# Epigenome association study for DNA methylation biomarkers in buccal and monocyte cells for female rheumatoid arthritis

**DOI:** 10.1038/s41598-021-03170-6

**Published:** 2021-12-10

**Authors:** Gary Craig, Howard Kenney, Eric E. Nilsson, Ingrid Sadler-Riggleman, Daniel Beck, Michael K. Skinner

**Affiliations:** 1 Arthritis Northwest, 105 W 8th Ave #6080, Spokane, WA 99204 USA; 2 Center for Reproductive Biology, School of Biological Sciences, Washington State University, Pullman, WA 99164-4236 USA

**Keywords:** Rheumatoid arthritis, Autoimmunity, Predictive markers

## Abstract

Genetics (i.e., mutations) has been assumed to be the major factor in rheumatoid arthritis (RA) etiology, but accounts for a minority of the variance in disease risk for RA. In contrast to genetics, the environment can have dramatic impacts on epigenetics that associate with disease etiology. The current study used buccal cells and purified blood monocytes from two different clinical cohorts involving Caucasian or African American female populations with or without arthritis. The differential DNA methylation regions (DMRs) between the control and RA populations were identified with an epigenome-wide association study. The DMRs (i.e., epimutations) identified in the buccal cells and monocytes were found to be distinct. The DMR associated genes were identified and many have previously been shown to be associated with arthritis. Observations demonstrate DNA methylation epimutation RA biomarkers are cell type specific and similar findings were observed with the two racial background populations. Rheumatoid arthritis susceptibility epigenetic diagnosis appears feasible and may improve the clinical management of RA and allowpreventative medicine considerations.

## Introduction

Rheumatoid arthritis (RA) is a chronic autoimmune inflammatory disease predominant in females with a worldwide prevalence of 0.5–1.0%^1^. The frequency of RA has increased in the past few decades, and is proportionally higher in North American populations^1,2^. RA is characterized by synovial hyperplasia and joint destruction^3^. RA impacts both African American and Caucasian populations, but does have higher comorbidity^4^ prevalence in African American populations. Coronary heart disease is more common in rheumatoid arthritis populations^4^, as in other chronic inflammatory diseases. Inflammatory lung disease is a common extra-articular manifestation^5^, along with rheumatoid nodules, secondary Sjogrens from endocrine gland inflammation, and more rarely rheumatoid vasculitis in this multisystem autoimmune disease. Correlations between RA and neurodegeneration have also been observed^6^.

Environment and lifestyle have been shown to influence the risk of RA^7^. Smoking and nutrition are two of the major factors linked to RA risk^8,9^. Nutrition and dieting habits also have a role in RA risk and progression^10^. Environmental toxicant exposures have been shown to be involved in the etiology of rheumatoid arthritis^11,12^. In addition to environmental toxicants, alcohol consumption is a risk factor for the incidence of RA^13^. Similar observations have been shown in various worldwide populations and ethnic backgrounds^14–16^. Therefore, environmental factors and lifestyle have a significant impact on the etiology and progression of rheumatoid arthritis.

Genetics has been assumed to be a major factor in rheumatoid arthritis etiology. Initially, gene associations were identified that involved a number of cellular pathways and immune related processes, such as the major histocompatibility complex (MHC), in particular the HLA-DRB1 and closely related genes^17^. These types of genetic mutation gene associations have been estimated to explain a minority of the variance in disease risk for RA^18,19^. A number of genome-wide association studies (GWAS) have been performed and identified hundreds of single-nucleotide polymorphisms (SNPs) that are associated with RA and speculated to impact a large number of biological processes^20^. Potential secondary gene associations suggest genetics can potentially explain 30% of familial disease cases^18^. Although these GWAS and similar gene impact studies have helped to better understand the molecular basis of RA, an alternate molecular process involving epigenetics is now assumed to be equally important and a significant factor in the etiology of RA^21^. Since environmental factors generally cannot directly change DNA sequence to alter genetic processes, the environmental impacts on RA etiology observed involve epigenetics. Epigenetics provides the molecular process for environmental factors such as nutrition and toxicants to impact genetics^22^. Therefore, an integration of environment, epigenetics and genetics is now thought to be involved in the etiology and progression of rheumatoid arthritis^21,23,24^.

Epigenetics is defined as “molecular factors and processes around DNA that regulate genome activity independent of DNA sequence, and are mitotically stable”^22^. Epigenetic factors include DNA methylation, histone modifications, non-coding RNA (ncRNA), chromatin structure and RNA methylation^22^. Although all these processes will be involved in RA, DNA methylation at 5-methylcytosine has been the primary epigenetic process investigated in rheumatoid arthritis^21,25,26^. Epigenome-wide association studies (EWAS) have been used to identify specific immune gene associations^21,27^, as have genome-wide investigations^21^. Some studies have investigated blood^28^, which contains over 20 different cell types. In contrast to genetics, where the DNA sequence is the same between different cell types, each individual cell type has a unique epigenome to give the cell type its specificity. Therefore, mixed cell type (e.g., blood or mixed T cell lymphocytes) analysis can be misleading and reflect changes in cell populations instead of epigenetic change. A number of RA studies have investigated purified cell populations to provide insights into rheumatoid arthritis etiology including B lymphocytes^29^, monocytes^30^, and synoviocytes^31–34^, which have distinct roles in RA etiology. Therefore, epigenetic analysis has provided insight into the pathology of rheumatoid arthritis^21,35^.

The potential role of inheritance of RA has been demonstrated through familial clusters and parental transmission of arthritis susceptibility^36–38^. A non-genetic form of inheritance has been previously described that involves epigenetic alterations in the germline (sperm and egg) and the inheritance to subsequent generations, termed epigenetic transgenerational inheritance^22,39^. When the germ cell transmits the altered epigenetics to the developing embryo stem cells, all subsequent somatic cells developed will have altered epigenomes and transcriptomes. Those cell types sensitive to the shift in epigenetics will have a susceptibility to develop disease later in life^22^. Therefore, various somatic cell populations could be used as surrogate biomarker cell types to identify disease susceptibility and disease conditions. The role of epigenetic biomarkers in autoimmune disease and rheumatoid arthritis has previously been discussed^40,41^. A cell type population previously shown to have epigenetic alterations that is functionally related to disease activity in RA is the monocyte^30,42^. For the development of a biomarker cell type for disease susceptibility, the buccal cells are easily obtained from a cheek swab and have a high level of purity. Therefore, the current study used purified monocytes and buccal cells from a Caucasian population to develop a potential biomarker for rheumatoid arthritis. In addition, a distinct African American population cohort was also used with collection of buccal cells. This is one of the first observations that human buccal cells can be used as a surrogate marker cell for the detection of disease associated epimutations. Both Caucasian and African American population clinical cohorts were used to compare non-arthritis individuals versus arthritis individuals to identify RA associated DNA methylation alterations, termed epimutations. A combined analysis of Caucasian and African American populations provided an epigenetic biomarker in buccal cells for RA that was more efficient for diagnosis of RA susceptibility than either population alone. These epimutations can potentially be used as a biomarker for RA to improve the detection and clinical management^43,44^ of the disease. The current study provides the proof of concept that buccal cell RA epigenetic biomarkers may exist, however, future larger clinical trial studies are required to optimize the epigenetic biomarker.

## Results

Females of similar age and race were collected for comparison of individuals with and without rheumatoid arthritis, Table 1. The white Caucasian non-Hispanic female samples were obtained by the Arthritis Northwest (ANW) Clinic in Spokane, Washington. The African American (AA) female samples were obtained by Dx Biosamples, LLC in San Diego, California, with sample collections in the Los Angeles California area. Institutional review board (IRB) approvals were obtained for the study from both sources. The individual sample information for age, race, and clinical information is presented in Table 1. Upon collection of buccal cell swabs, the samples were frozen at − 20 °C then shipped on dry ice and stored at − 80 °C until use. The procedure and sample processing for buccal cells is presented in the Supplemental Methods. For Caucasian ANW clinic patients, a blood sample was also obtained and shipped immediately on ice for isolation of monocytes, as described in the Methods. Monocytes were purified with an antibody bead procedure, as previously described^45^. After isolation, the monocytes were stored at- − 80 °C prior to use. The Caucasian (CC) buccal cell control without RA (n = 13) and with RA (n = 13) were compared. The Caucasian monocyte cell control without RA (n = 13) and with RA (n = 13) were compared. The African American (AA) buccal cell control without RA (n = 9) and with RA (n = 13) were compared. A combination of Caucasian and African American samples without (n = 23) and with RA (n = 26) were compared. The individual sample information is presented in Table 1 and provides clinical information and RA diagnostic information. The Caucasian samples had RA diagnostic assays of Rheumatoid Factor autoantibody (RF) and Citrullinated Peptide autoantibody (CCP), and more qualitative RA activity assays of Clinical Disease Activity Index (CDAI) with DAS28 and RAPID3 supportive analysis, Table 1.

**Table 1:** Buccal and monocyte samples and clinical information.

Subject #	Group	Age	Race	Gender	RA Diagnostic Information
**(A) Arthritis Northwest Caucasian Population**					
ANWC01	Control	74	Caucasian	F	Not Applicable
ANWC02	Control	56	Caucasian	F	Not Applicable
ANWC03	Control	39	Not specified	F	Not Applicable
ANWC04	Control	56	Caucasian	F	Not Applicable
ANWC05	Control	59	Caucasian	F	Not Applicable
ANWC06	Control	68	Caucasian	F	Not Applicable
ANWC07	Control	55	Caucasian	F	Not Applicable
ANWC08	Control	70	Caucasian	F	Not Applicable
ANWC09	Control	49	Caucasian	F	Not Applicable
ANWC10	Control	62	Caucasian	F	Not Applicable
ANWC11	Control	30	Caucasian	F	Not Applicable
ANWC12	Control	51	Caucasian	F	Not Applicable
ANWC13	Control	45	Caucasian	F	Not Applicable
	Mean age ± SEM	54.9 ± 3.4			
ANWT01	Rheumatoid arthritis	73	Caucasian	F	High RA activity, RF + , CCP + , CDAI +
ANWT03	Rheumatoid arthritis	61	Caucasian	F	High RA activity, RF + , CCP + , CDAI +
ANWT04	Rheumatoid arthritis	41	Caucasian	F	High RA activity, RF + , CCP + , CDAI +
ANWT05	Rheumatoid arthritis	56	Caucasian	F	High RA activity, RF + , CCP + , CDAI +
ANWT06	Rheumatoid arthritis	57	Caucasian	F	High RA activity, RF + , CCP + , CDAI +
ANWT09	Rheumatoid arthritis	66	Caucasian	F	High RA activity, RF + , CCP + , CDAI +
ANWT10	Rheumatoid arthritis	50	Caucasian	F	Moderate RA activity, RF + , CDAI +
ANWT12	Rheumatoid arthritis	74	Caucasian	F	Moderate RA, activity RF + , CDAI +
ANWT13	Rheumatoid arthritis	54	Caucasian	F	High RA activity, RF + , CCP + , CDAI +
ANWT14	Rheumatoid arthritis	68	Caucasian	F	High RA activity, RF + , CCP + , CDAI +
ANWT15	Rheumatoid arthritis	26	Caucasian	F	High RA activity, RF + , CCP + , CDAI +
ANWT16	Rheumatoid arthritis	48	Caucasian	F	High RA activity, RF + , CCP + , CDAI +
ANWT17	Rheumatoid arthritis	48	Native American	F	High RA activity, RF + , CCP + , CDAI +
	Mean age ± SEM	55.5 ± 3.7			
**(B) DxBiosamples African American Population**					
AH_10	Control	55	African American	F	Not Applicable
AH_12	Control	52	African American	F	Not Applicable
AH_13	Control	55	African American	F	Not Applicable
AH_14	Control	57	African American	F	Not Applicable
AH_15	Control	52	African American	F	Not Applicable
AH_16	Control	58	African American	F	Not Applicable
AH_17	Control	65	African American	F	Not Applicable
AH_18	Control	61	African American	F	Not Applicable
AH_19	Control	57	African American	F	Not Applicable
	Mean age ± SEM	56.8 ± 1.3			
AH_20	Rheumatoid arthritis	55	African American	F	High RA activity, CDAI +
AH_22	Rheumatoid arthritis	51	African American	F	High RA activity, CDAI +
AH_23	Rheumatoid arthritis	53	African American	F	High RA activity, CDAI +
AH_24	Rheumatoid arthritis	56	African American	F	Moderate RA activity, CDAI +
AH_25	Rheumatoid arthritis	58	African American	F	High RA activity, CDAI +
AH_26	Rheumatoid arthritis	52	African American	F	High RA activity, CDAI +
AH_27	Rheumatoid arthritis	53	African American	F	Moderate RA activity, CDAI +
AH_28	Rheumatoid arthritis	50	African American	F	High RA activity, CDAI +
AH_29	Rheumatoid arthritis	51	African American	F	High RA activity, CDAI +
AH_30	Rheumatoid arthritis	50	African American	F	High RA activity, CDAI +
AH_31	Rheumatoid arthritis	52	African American	F	High RA activity, CDAI +
AH_32	Rheumatoid arthritis	52	African American	F	High RA activity, CDAI +
AH_33	Rheumatoid arthritis	63	African American	F	High RA activity, CDAI +
	Mean age ± SEM	53.5 ± 1.0			

The samples were provided by ANW Clinic, Spokane, WA and Dx Biosamples, San Diego, CA, that collected and obtained the clinical information of age, sex, clinical diagnosis RA and site collection. The mean ± SD for case and control samples are provided for age. A student t-test was used to assess any statistical differences between RA and control groups, and none were found. *RF* + Rheumatoid Factor autoantibody positive, *CCP* + Cyclic citrullinated peptide autoantibody positive, *CDAI* Clinical Disease Activity Index positive with high RA activity > 22 and moderate activity 10–22. CDAI supportive assays at DAS28 and RAPID3 positive.

**Figure 1:**
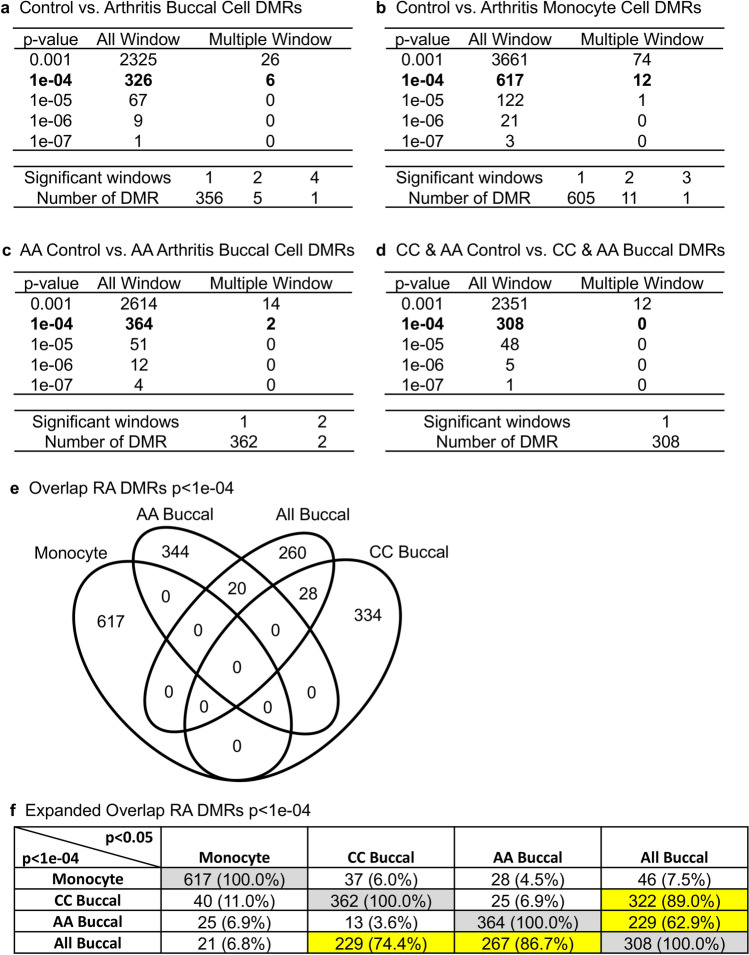
Rheumatoid arthritis (RA) DMR identifications. **(a)** Caucasian control versus RA buccal cell DMR analysis. **(b)** Caucasian control versus RA monocyte cell DMR analysis. **(c)** African American (AA) control versus RA buccal cell DMR analysis. **(d)** Combined Caucasian (CC) and African American (AA) control versus RA buccal cell DMR analysis. The number of DMRs found using different *p*-value cutoff thresholds. The all window column shows all DMRs. The multiple window column shows the number of DMRs containing at least two adjacent significant windows and the number of DMRs with each specific number of significant windows at a *p*-value threshold of *p* < 1e−04. **(e)** Venn diagram overlap of the RA DMRs at *p* < 1e−04 for the Caucasian monocyte, buccal and AA buccal, and combined CC and AA (All) buccal. **(f)** Extended overlap with a comparison of RA DMRs in the different comparison at *p* < 1e−04 versus horizontal *p* < 0.05 for the different comparisons. The overlapping DMR numbers and percent (%) of the total is presented. The highlighted overlaps for All buccal and overlaps indicated.

The DNA was extracted from each sample as described in the Supplemental Methods. The DNA was then sonicated to 150–300 bp fragments and used for a methylated DNA immunoprecipitation (MeDIP) protocol, as described in the Supplemental Methods. This involved an antibody to 5-methylcytosine and a magnetic bead procedure^46^. This immunoprecipitated methylated DNA was then used to generate a sequencing library for an MeDIP-Seq analysis, as described in the Supplemental Methods. MeDIP-Seq allows for greater than 90% of the genome to be examined for this EWAS analysis. Each sample for the MeDIP-Seq analysis had approximately 25 million reads, and the quality control details are presented in the Supplemental Methods. The EdgeR statistical analysis was used to identify differential DNA methylation regions (DMRs). Various *p*-value thresholds are presented for each of the comparisons, and *p* < 1e−04 was selected for subsequent analysis, Fig. 1. The majority of DMRs had one significant 1 kb window, but some had multiple windows. The Caucasian (CC) control versus RA buccal had 362 DMRs (Fig. 1a), CC control versus RA monocytes 617 DMRs (Fig. 1b), AA control versus AA RA buccal had 364 DMRs (Fig. 1c), and combination of CC and AA RA all buccal had 308 DMRs (Fig. 1d). A venn diagram of the different comparison DMRs at *p* < 1e−04 had no overlap, except for the all buccal combined CC and AA that had approximately a 10% overlap, Fig. 1e. An extended overlap of the *p* < 1e−04 DMR comparisons with the others at a reduced statistical threshold of *p* < 0.05 was used to determine if a DMR overlap is present at a reduced threshold, Fig. 1f. From the horizontal row, the same cell type has 100% overlap, as anticipated. The same row allows potential overlaps at a reduced statistical threshold to be identified. The overlap was 11% between the Caucasian monocyte and buccal cell. The other overlaps were between 3 and 7%, Fig. 1f. Interestingly, the combined CC and AA (all buccal) analysis had a 74% overlap with the CC buccal and 87% with the AA buccal (Fig. 1f highlight), suggesting the combined analysis DMR set is optimal for an RA biomarker. Therefore, the majority of the DMRs for each specific distinct comparison were cell and race specific, while the combined CC and AA analysis had good overlap, Fig. 1f.

**Figure 2:**
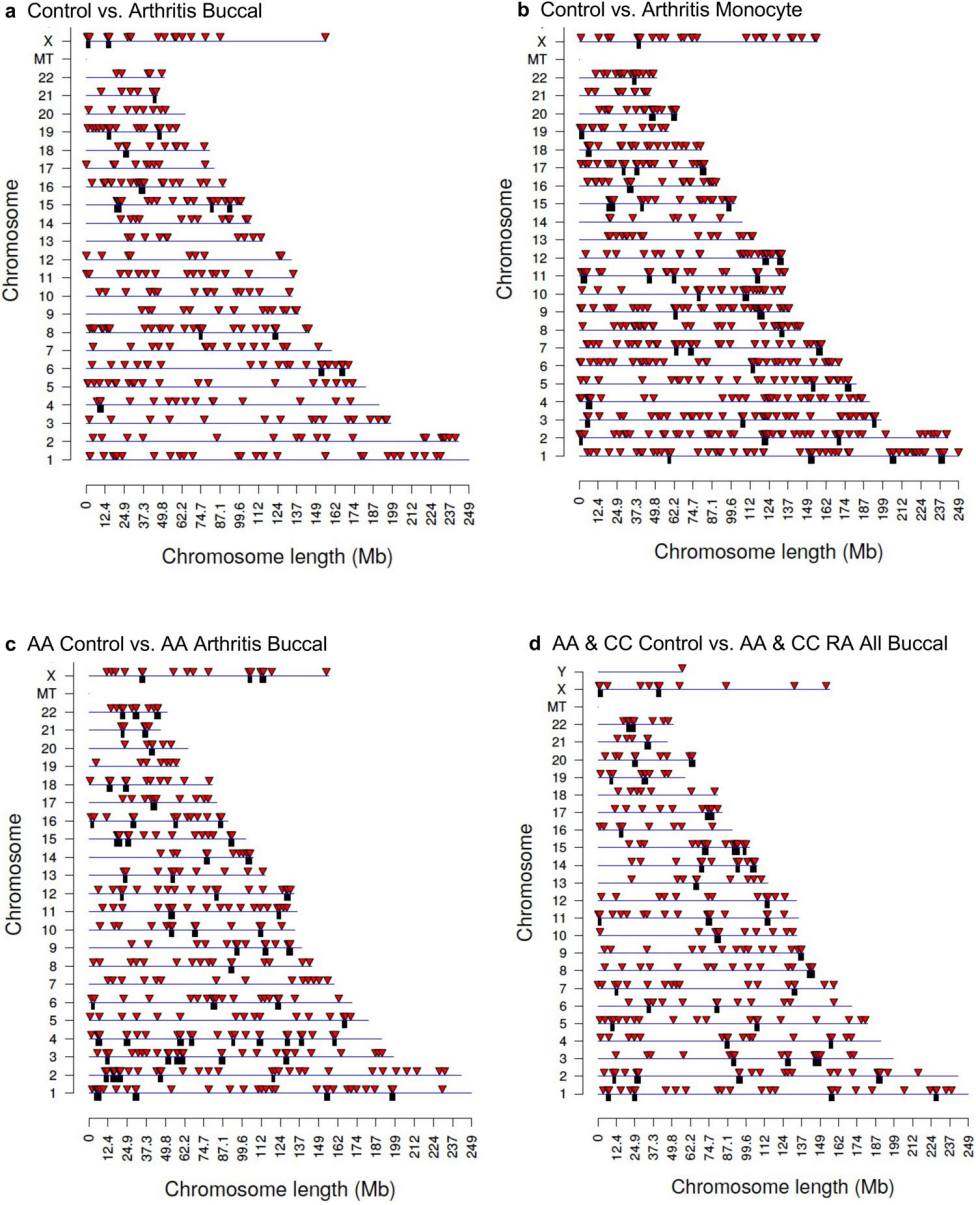
RA DMR chromosomal locations and principal component analysis (PCA). The DMR locations on the individual chromosomes are identified. All DMRs at a *p*-value threshold of *p* < 1e−04 are shown with the red arrowheads and clusters of DMRs with the black boxes. **(a)** Caucasian control versus RA buccal DMRs. **(b)** Caucasian control versus RA monocyte DMRs. **(c)** AA control versus RA buccal DMRs. **(d)** Combined CC and AA for All buccal control versus RA buccal DMRs. All DMRs at a *p*-value threshold of *p* < 1e−04.

**Figure 3:**
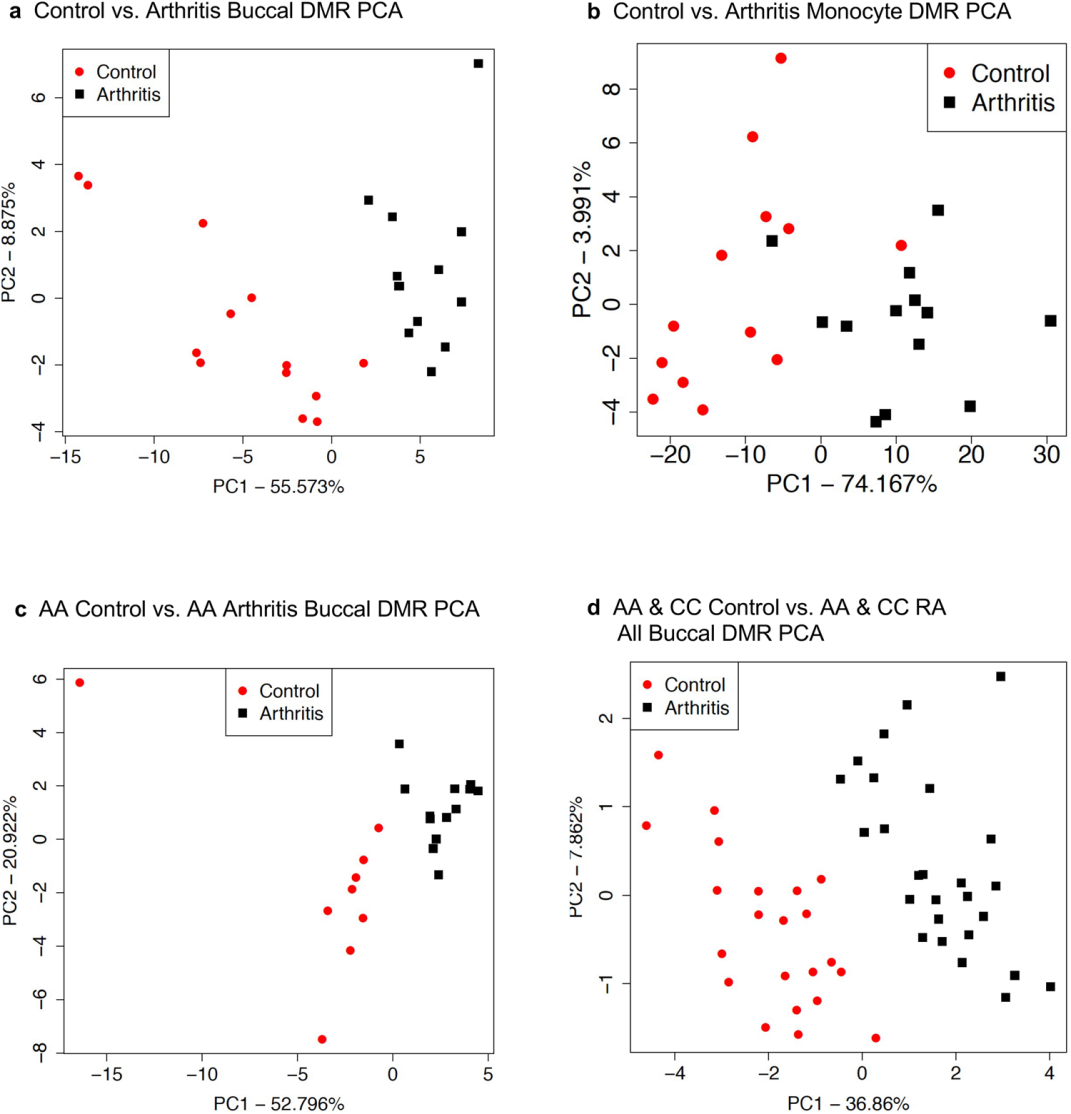
Control versus RA DMR principal component analysis (PCA). PCA analysis for DMRs at *p* < 1e−04. **(a)** Caucasian control versus RA. **(b)** Caucasian monocyte DMR PCA. **(c)** AA control versus AA RA buccal DMR PCA. **(d)** CC and AA all buccal control versus all RA buccal DMR PCA.

The information for each comparison set of DMRs is presented in Supplemental Tables S1, S2, S3 and S4. The DMR name, chromosomal location, start and stop nucleotide sites, length bp, number of 1 kb significant windows, EdgeR *p*-value, maximum log-fold change (maxLFC) (i.e., positive values being an increase in DNA methylation, and negative values a decrease in DNA methylation), CpG number and density, and DMR associated gene and gene category, are presented in Supplemental Tables S1–S4. The chromosomal locations of the DMRs are presented in Fig. 2a–d with chromosome number and location (megabase) indicated for each with a red arrowhead. The black boxes indicate a cluster of DMRs. All the chromosomes contain DMRs with this genome-wide analysis, Fig. 2a–d. Therefore, the RA DMRs (e.g., epimutations) are genome-wide. The DMR characteristics demonstrate a low CpG density, termed a CpG desert, of 1–3 CpG/100 bp, Supplemental Figure S1, for all the comparison DMRs. The sizes of the DMRs are predominantly 1 kb in size with some being 2–4 kb in size, Supplemental Figure S1, for all the comparisons. A comparison of the principal components in a Principal Component Analysis (PCA) demonstrates that the DMR components for the control versus RA cluster separately for each of the comparison groups, Fig. 3a–d. The only exception is with the Caucasian control versus RA monocytes, Fig. 3b, that has one control and one RA each that overlap with the PCA cluster. The overlapping RA group DMR at PC1 = − 8 and PC2 = 2.2 was 74 year age. The other individuals at > 70 year age were the DMRs at PC1 = − 4 and PCR = − 18, PC1 = 31 and PC2 = − 0.5, and PC1 = − 4 and PC2 = 9.2, Fig. 3b. Higher variability was observed with the > 70 year individuals. Therefore, the PCA indicates principal components of the DMRs are predominantly distinct between the control and arthritis groups. In addition, the Native American and individual not specified for race (Table 1) were not outliers with the PCA analysis (Fig. 3), so we included in the analysis. The Caucasian sample sets had a few 70 year and 30 year outliers compared to the mean of approximately 55 year samples, Table 1a. Reanalysis of the comparison sample sets deleting these samples did not impact significantly the DMR number nor statistics. Future studies are needed with larger sample sizes to more accurately determine the impact of age on the RA epigenetic biomarkers. The genome-wide statistical analysis with EdgeR demonstrated the monocyte analysis provided a false discovery rate (FDR) of < 0.1, but the buccal analysis gave an FDR of approximately 0.2. Therefore, the buccal cell DMR analysis was more variable than the monocyte analysis. The significance of each DMR is indicated with minimum *p*-value and minimum FDR in the Supplemental Tables S1–S4. Future studies require increased sample size when using buccal cells as a marker cell. The potential DMR biomarkers for rheumatoid arthritis identified demonstrated predominantly unique chromosomal locations for each comparison (Fig. 2), but similar genomic features, Supplemental Tables S1–S4. The RA DMR biomarkers appear cell type specific, and are distinct between the Caucasian and African American populations when analyzed separately. However, a combined CC and AA all buccal cell analysis did show strong overlap among the groups, Fig. 1f.

**Figure 4:**
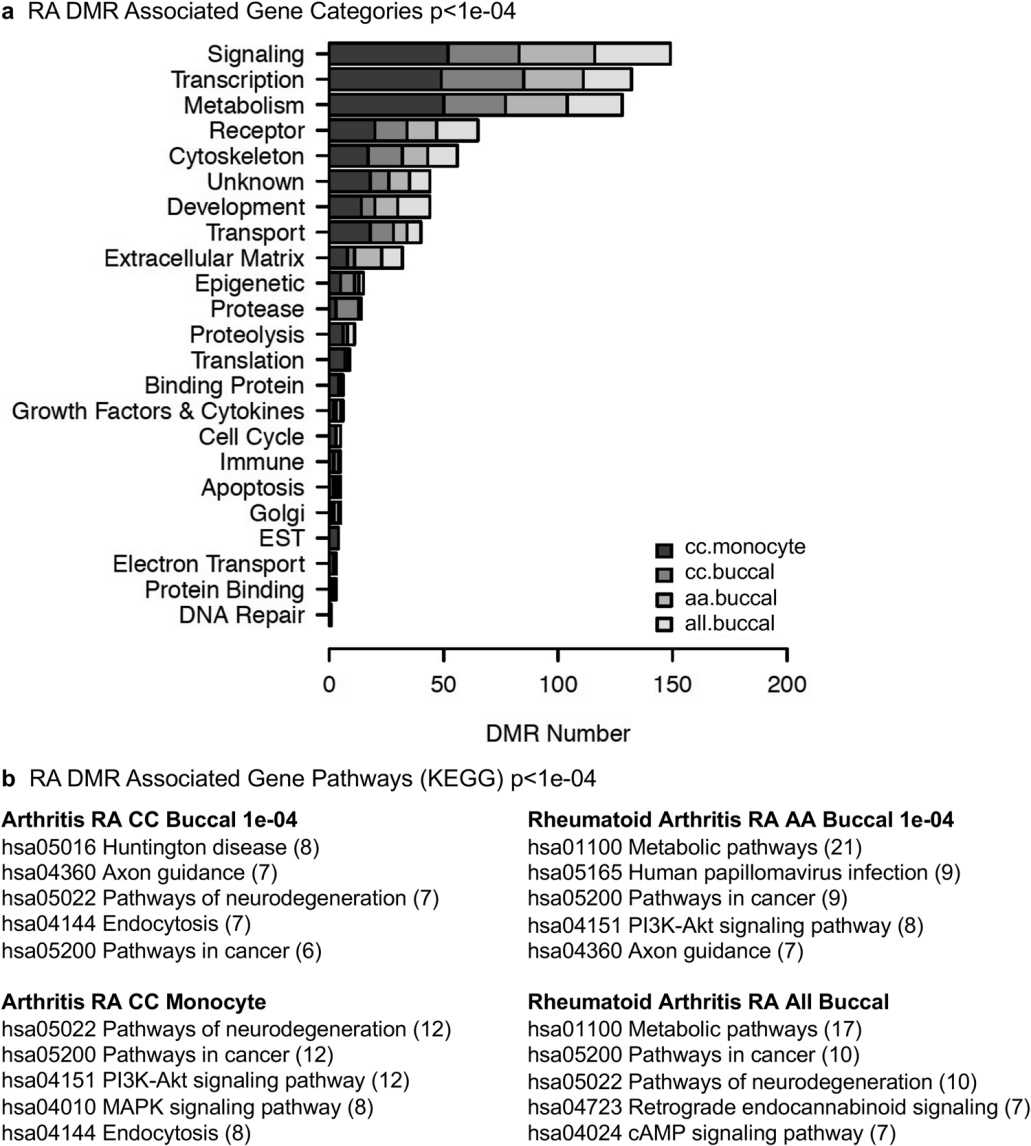
RA DMR associated gene categories and pathways. **(a)** DMR associated gene categories. DMR numbers at a *p*-value threshold *p* < 1e−04 are shown. The comparison DMR key is inset. **(b)** DMR associated gene pathways. The pathways common for two or more comparisons are presented. Number in bracket is number of DMR associated genes in pathway.

Less than half of the RA DMRs identified with each comparison had DMR associated genes within 10 kb of the DMR, Supplemental Tables S1–S4. The 10 kb distance is used to include the proximal and distal promoter regions of the genes. The RA DMR associated gene categories were identified and are presented in Fig. 4a. The DMR numbers for each associated gene functional category are presented for each comparison. The signaling, transcription, metabolism and receptor are predominant for each of the DMR comparisons. This does reflect the major gene categories within the human genome with metabolism, transcription and signaling being the largest gene categories. This gene category analysis was for an individual DMR gene association analysis and not to reflect group combined function. In contrast, a gene pathway analysis (KEGG) was performed, and those pathways in two or more different comparison DMR associated genes with similar function were identified in Fig. 4b. The metabolic pathway was excluded as it is present in all comparisons and over-represented in pathway analysis. The common signaling pathways include pathways in cancer, pathways in neurodegenerative disease, and specific pathways such as P13K-Akt, Fig. 4b.

**Figure 5:**
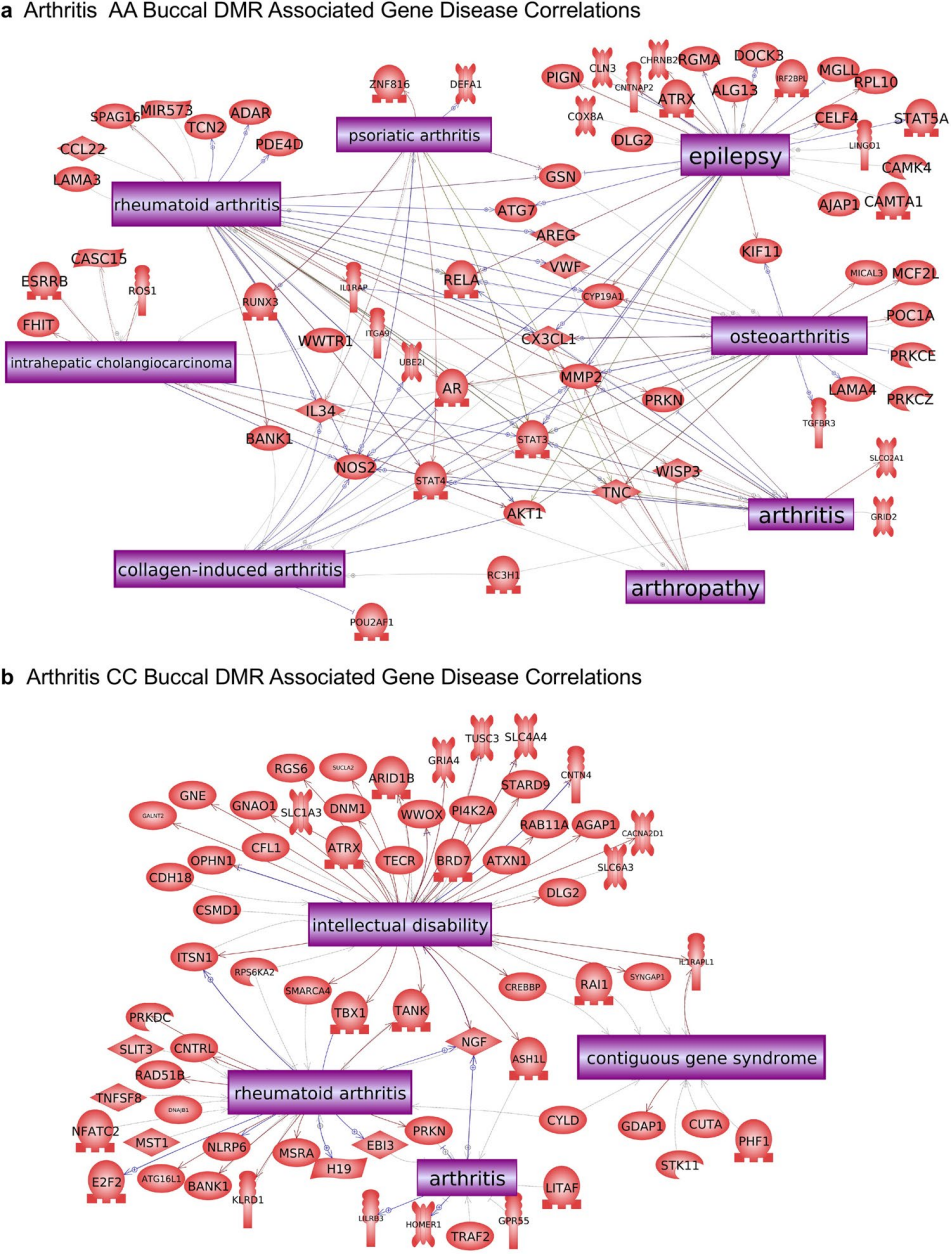
DMR associated genes from the current study were compared to genes associated with arthritis in the published literature using Pathway Studio software (Elsevier, Inc.). Those that were in common are depicted. **(a)** African American (AA) buccal cell RA DMR associated gene disease correlations. **(b)** Caucasian buccal DMR associated gene disease correlations.

**Figure 6:**
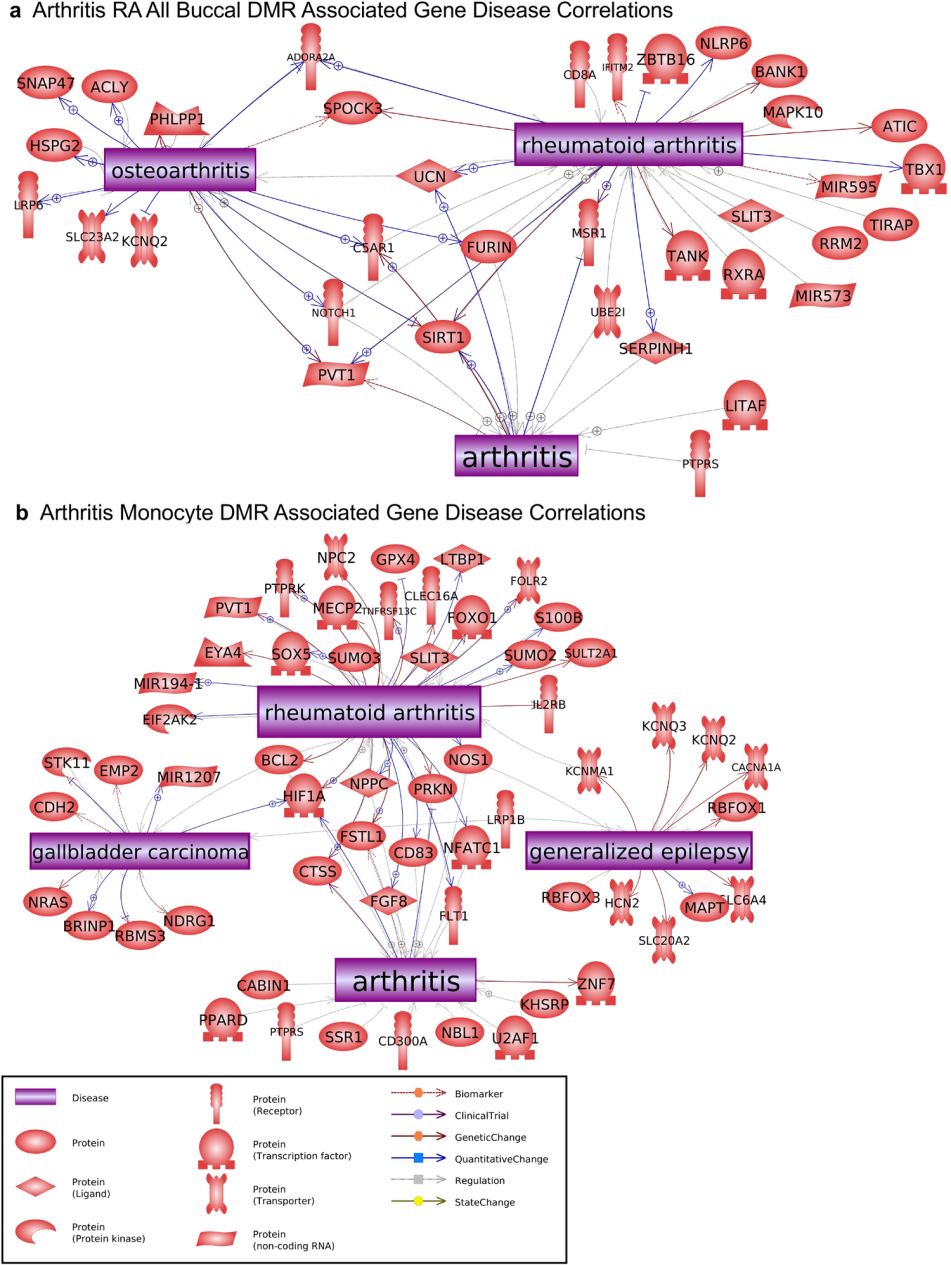
DMR associated genes from the current study were compared to genes associated with arthritis in the published literature using Pathway Studio software (Elsevier, Inc.). Those that were in common are depicted. **(a)** Combined CC and AA all buccal RA DMR associated gene disease correlations. **(b)** Caucasian monocyte RA DMR associated gene disease correlations. The gene function symbol index inset.

**Figure 7:**
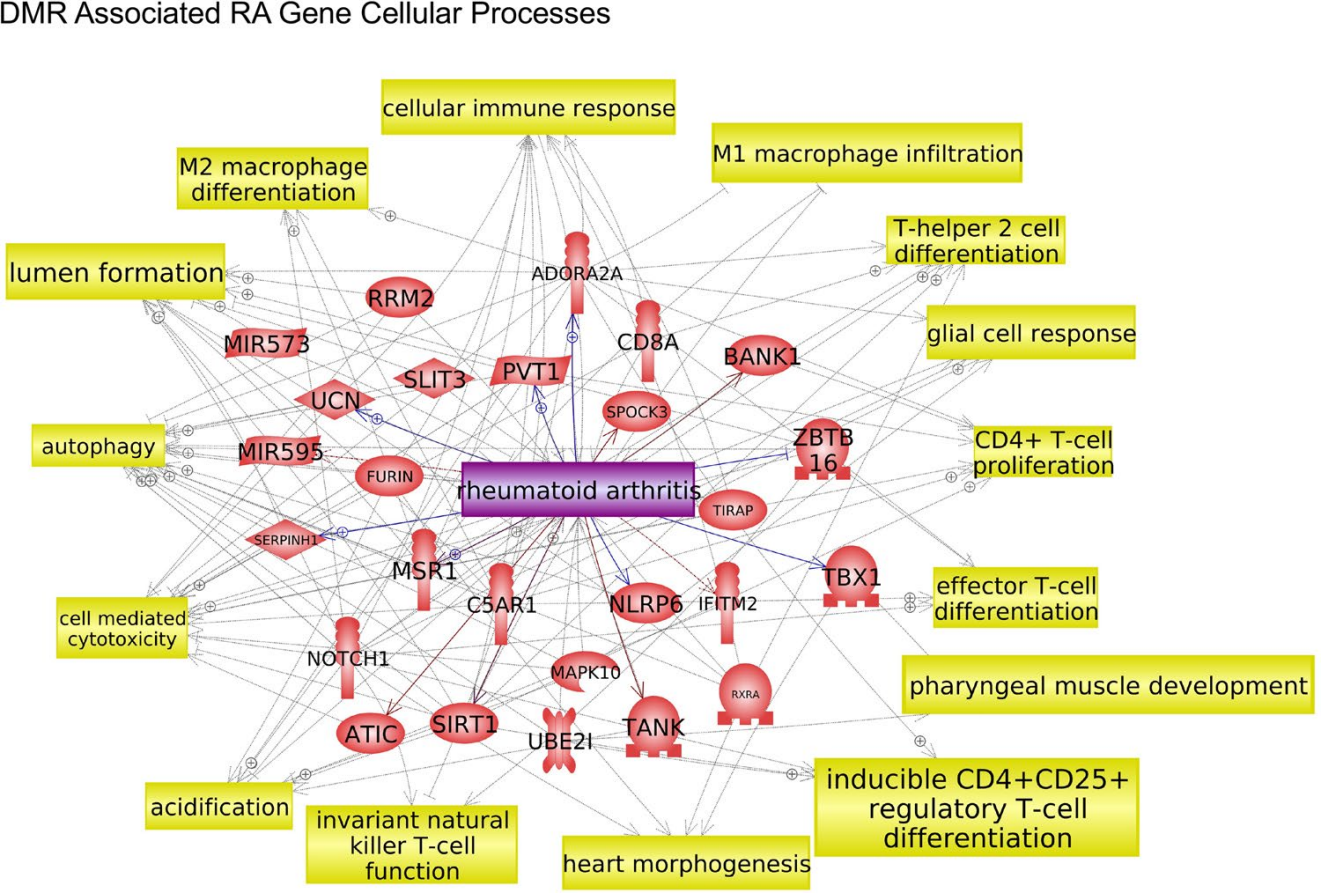
DMR associated RA genes were correlated with known RA cell processes in the published literature using Pathway Studio software (Elsevier, Inc.). The DMR associated genes from all comparisons were linked to the RA cell processes.

The final analysis correlated the RA DMR associated genes with pathologies and processes. Pathway Studio was used to identify the associated pathologies, as described in the Supplemental Methods. The African American RA buccal cell DMR associated gene correlations to diseases are presented in Fig. 5a. Six of the major disease correlations were arthritis related. The others are cancer and neurodegenerative related. Therefore, the predominant African American RA DMRs in buccal cells are associated with arthritis pathologies, Fig. 5a. The Caucasian buccal cell RA DMR associated gene correlations with rheumatoid arthritis and arthritis pathologies are shown, Fig. 5b, with contiguous gene syndrome and intellectual disability as additional pathologies significantly correlated. The combination CC and AA analysis also provided DMR associated genes and arthritis gene associations, Fig. 6a. The Caucasian monocyte DMRs also predominantly associated with arthritis and RA correlated genes, with additional epilepsy and carcinoma associated genes, Fig. 6b. Observations demonstrate the RA DMRs identified with all the comparisons showed significant connections to genes correlated to arthritis pathologies, including rheumatoid arthritis. An additional analysis used the DMR associated genes for RA from each of the comparisons to identify known RA cellular process correlations, Fig. 7. Previous rodent and human studies have identified known gene correlations with RA associated cell processes, and these same genes and correlations were observed in the current study, Fig. 7 and Supplemental Table S5. Over 25 different RA gene cell processes were identified with a statistical significance *p* < 1e−06, with the four most significant being protein regulators of immune response, inflammatory response, innate immune response, and cellular immune response, Supplemental Table S5. These DMR associated rheumatoid arthritis gene cell process correlations are shown in Fig. 7 and Supplemental Table S5. Therefore, the RA DMR associated genes identified in the current study correlated to previously identified RA associated genes and cellular processes, as well as identified potential new RA associated genes.

## Discussion

Rheumatoid arthritis impacts approximately one percent of the worldwide population, and some areas such as North America have a higher incidence of RA among the population^21^. RA is generally diagnosed today after the onset of the disease when clinical characteristics develop. Therefore, most diagnosis is based on symptomatic parameters. Although several RA biomarkers and diagnostics have been developed^47,48^, none are focused on early-stage biomarkers that can be used prior to the onset of clinical symptoms. The advantage of epimutations such as DNA methylation biomarkers is that they have been shown to develop early in life, or through epigenetic inheritance ancestrally, such that the biomarkers have the potential to act as diagnostics for preventative medicine treatments^41,49^. Such an early-stage RA biomarker or diagnostic has not been developed and is the focus of the current study. The clinical management and treatment of the disease has been shown to be more effective as a preventative treatment prior to disease onset. An example is the use of the chemotherapy tamoxifen, which has a low efficiency in treating breast cancer once the cancer has developed. However, tamoxifen can be effective as a preventative treatment for a female in her 30 s prior to disease development^50^. The possibility that current RA therapeutics may be more efficient as preventative treatments prior to the onset of disease remains to be investigated. The key factor in allowing these types of preventative therapeutics to be considered is the availability of an RA biomarker that can be used prior to the onset of disease or symptoms. The current study is designed to develop an epigenetic biomarker for RA that, due to the nature of environmental impacts on developmental programming of epigenetics, can potentially also act as an RA susceptibility diagnostic to facilitate a preventative medicine approach for the disease.

Genome-wide association studies (GWAS) that are used to identify genetic mutations have generally found that less than 1% of the population with a specific disease has correlated genetic mutations. This has become the major factor inhibiting effective genetic diagnostics for disease^51^. In contrast, epigenome-wide association studies (EWAS) often identify epimutations that are present in the majority of the population with a specific disease^52^. This allows EWAS studies to use smaller sample sizes compared to GWAS studies. The current study found RA DNA methylation DMR sites were present in the populations with RA when compared to those without, Fig. 1a–c. As previously demonstrated, epigenetic biomarkers are anticipated to be more efficient as a tool for disease and disease susceptibility analysis^53,54^. In the current study, two distinct control and RA clinical cohort populations were obtained for analysis. The Caucasian and African American populations were obtained at different locations under separate IRBs. Similar observations were made with both these clinical cohorts. Although the current study has the limitation of not having a blinded test set or distinct expanded clinical cohort, the current study does use two distinct clinical populations from different racial backgrounds, locations, and clinical sites for comparison. Interestingly, the Caucasian and African American population buccal cells had distinct RA DMRs with only a 3.6% overlap of 13 DMRs when analyzed separately, Fig. 1e. Observations suggest that ethnic background and race need to be considered in the development of epigenetic DNA methylation diagnostics for disease. One variable in this analysis is that the Caucasian samples were collected in Spokane, Washington, while the African American samples were collected in Los Angeles, California. Since epigenetics is environmentally responsive and inherited^39^, the demographics and collection regions will need to be considered. Spokane Washington is a moderate size city with less environmental contaminants versus Los Angeles, which is a large urban city with higher levels of pollutant exposure such as air pollution. The generational backgrounds will also be distinct between the populations, and ethnic and racial impacts on epigenetics has been observed^55^. However, when the CC and AA populations were combined, a DMR set with strong overlap with both the individual comparisons was identified, Fig. 1f. Therefore, expanded future studies should incorporate diverse race and ethnic background in the development of epigenetic DMR rheumatoid arthritis diagnostics. Observations from the current study demonstrate similar levels of epigenetic change (i.e., numbers of RA DMRs observed), but the majority of DMRs were distinct between the Caucasian and African American populations. This is one of the first observations for potential race and ethnic background impacts on the development of epigenetic disease biomarkers. Interestingly, the combination of the populations within the analysis generated a more efficient epigenetic biomarker for female RA susceptibility. Although the current study only provides the proof of concept potential biomarkers for RA may be developed, expanded clinical trials with larger sample size are now required. Diverse populations need to be considered in future studies and potential commercialization of such disease biomarkers.

The current study in the Caucasian population compared two different cell populations for the analysis of potential RA biomarkers. The monocytes are an immune related cell that has been shown to develop molecular alterations in RA patients relevant to the disease pathology^56^. As previously described, the alterations in epigenetics observed in the current study are relevant to RA, Figs. 6 and 7. In contrast, the buccal cells from a cheek swab have no distinct relationship to the RA disease pathology or etiology, but functions as a surrogate marker cell for epigenetic alterations. Buccal cells are one of the easiest purified cell populations to collect, which is required for epigenetics, and they are the least invasive for the patient. The blood collection, followed by monocyte cell purification, would not be an efficient biomarker cell to develop for RA compared to the buccal cell. Since the majority of disease has an early developmental exposure or impact that promotes the developmental origins of health and disease (DOHaD), all cell types in the body can be impacted by these early life events. Epigenetics is the primary mechanism involved in these developmental origins of disease, since genetic mutations are a minor component of disease correlations. Therefore, ancestral and early life exposure can impact the epigenetics of all cell types in the body such that marker cells like the buccal cells can be used to reflect epigenetic alterations associated with later life disease susceptibility and diagnosis. Although the buccal cells are simply a marker surrogate cell for epigenetic alterations, the current study demonstrates that a number of the RA DMRs identified do have associations with RA and generational arthritis, Figs. 5, 6 and 7. One of the major observations of the current study is that easily collected buccal cells may act as an effective surrogate marker cell for the epigenetic diagnosis of disease susceptibility.

Many of the DMR associated genes correlated with previously identified RA disease associated genes, Figs. 5, 6 and 7. The DMR associated genes were within 10 kb of the gene to consider the proximal and distal promoters of the genes. Although some DMRs are only within the promoter region, the majority of DMRs overlapped with the gene. The RA DMR associated genes suggest a potential regulatory role, but future studies are needed to demonstrate a potential to regulate gene expression. Approximately 50% of the DMRs have an increase in DNA methylation and the rest a decrease in DNA methylation, Supplemental Tables S1–S4 (log fold change, max LFC value). Interestingly, the DMR associated genes for the Caucasian buccal cell and monocyte cells both had RA associated genes, but were distinct, Figs. 5, 6 and 7. Some of the RA associated genes common among the different comparisons included Sox5, Fox01, FGF8, TNFSF8, TANK and H19. Two genes not in the DMR associated genes at *p* < 1e−04 that have been shown to be correlated with RA are HLA-DRB1^57^ and 14-3-3eta^58^. The HLA-DRB1 is within the immune complex gene group and is associated with RA therapeutics^59^. The 14-3-3 eta gene has been shown to be a useful RA biomarker^60^. Further analysis at a reduced statistical threshold demonstrated DMRs within 10 kb of these genes to include the proximal and distal promoter region for HLA-DRB1 in the African American buccal (*p* < 0.02), and the 14-3-3 eta in the Caucasian monocyte (*p* < 0.0003) and buccal (*p* < 0.03), and African American buccal (*p* < 0.04). Therefore, the DMRs at reduced statistical thresholds will also be important to consider in the RA disease etiology. Previous studies have validated the functional and clinical roles of the DMR associated genes in RA disease etiology, Fig. 7 and Supplemental Table S5. The RA DMRs identified in the different comparisons provides insight into potentially important epimutations (e.g., DMRs) that provide a molecular mechanism for how environmental exposures and lifestyle impact the onset of RA.

The current study identifies potential rheumatoid arthritis (RA) biomarkers with an EWAS for epimutations in control versus RA patients. An interesting observation was that the Caucasian and African American populations had distinct epimutations with increased DMR overlaps in the buccal cells. Interestingly, a combination of the Caucasian and African American samples analysis identified a DMR set that had strong overlap for both and provides a more efficient and optimal potential RA biomarker. Monocytes had DMR epimutations that were distinct from the buccal cells, but both cells had DMR associated genes previously shown to be involved in RA. Observations indicate that buccal cells can have RA DMR associated genes and provide a potential biomarker cell for RA disease susceptibility. Larger clinical trials with a greater number of RA patients are now needed to confirm and validate the observations and DMRs identified in the current study. These expanded trials also need to identify RA susceptibility in patients without the onset of RA to explore the ability to initiate preventative therapeutic experiments to delay or prevent the onset of RA later in life. The availability of such RA susceptibility biomarkers will allow preventative medicine approaches to be considered. The current study provides the proof of concept that such RA epigenetic biomarkers may potentially be developed.

## Methods summary

### Clinical sample collection

Two independent single center prospective and open clinical studies were performed. The Arthritis Northwest Clinic (ANW) in Spokane, Washington, USA and Dx Biosamples, LLC in San Diego, California, USA provided samples for the current study. The participant approval and informed consent was obtained from all participants prior to the clinical sample collection. The study protocols were approved by the Quorum Review Institutional Review Board (IRB) for the ANW clinic with code # AE010831, and the Dx Biosamples company with code IORG # 0006584, and IRB # 00007904. All research was performed in accordance with relevant guidelines/regulations. The study was not designed for, nor did the IRB involve, the ability of reporting patient clinical information to be correlated. The Caucasian and African American buccal cells and Caucasian monocytes were analyzed as described in the Supplemental Methods. The buccal samples were frozen at − 20 °C, shipped on dry ice and then stored at − 80 °C prior to analysis. The monocytes were shipped immediately on ice for antibody bead isolation, as described in the Supplemental Methods, then stored at − 80 °C prior to analysis, as described in the Supplemental Methods.

### Epigenetic analysis, statistics and bioinformatics

Buccal cell and monocyte DNA were isolated, as previously described^61^. Methylated DNA immunoprecipitation (MeDIP), followed by next generation sequencing (MeDIP-Seq) was performed. MeDIP-Seq, sequencing libraries, next generation sequencing, and bioinformatics analysis were performed as described^61^, and are found in the Supplemental Methods. The statistical analysis and validation protocols were performed as previously described^61^, and are found in the Supplemental Methods. All molecular data has been deposited into the public database at NCBI (GEO # GSE186179), and R code computational tools are available at GitHub (https://github.com/skinnerlab/MeDIP-seq) and www.skinner.wsu.edu. Lists of DMR associated genes were analyzed for functional relationships using the KEGG database (https://www.genome.jp/kegg/pathway.html) and Pathway Studio software (version 12.2.1.2: Database of functional relationships and pathways of mammalian proteins; Elsevier).

### Ethics approval

The participant approval and informed consent was obtained from all participants prior to the clinical sample collection. The IRB study was approved by the Quorum Review IRB Committee for the Arthritis Northwest (ANW) Clinic, Spokane, WA, USA, with code IRB # AE010831 and Dx Biosamples, LLC, San Diego, CA, USA with code IORG # 0006584, and IRB # 00007904. All research was performed in accordance with relevant guidelines/regulations.

## Supplementary Information

Supplementary Information 1.

Supplementary Figure S1.

Supplementary Table S1.

Supplementary Table S2.

Supplementary Table S3.

Supplementary Table S4.

Supplementary Table S5.

## Data Availability

All molecular data has been deposited into the public database at NCBI (GEO # GSE186179), and R code computational tools are available at GitHub (https://github.com/skinnerlab/MeDIP-seq) and www.skinner.wsu.edu.

## Abbreviations

RA: Rheumatoid arthritis
EWAS: Epigenome-wide association study
DMRs: Differential DNA methylation regions
GWAS: Genome-wide association study
MeDIP: Methylated DNA immunoprecipitation
MeDIP-Seq: Methylated DNA immunoprecipitation followed by next generation sequencing
LFC: Log-fold-change
PCA: Principal component analysis
