# Supplementary material for: Epigenome association study for DNA methylation biomarkers in buccal and monocyte cells for female rheumatoid arthritis
Source: Sci Rep. 2021 Dec 10;11:23789. doi: 10.1038/s41598-021-03170-6 (PMC8664902; doi:10.1038/s41598-021-03170-6)
Supplement: Supplementary file 1 — Supplementary Information 1. [file 41598_2021_3170_MOESM1_ESM.pdf]

Epigenome Association Study for DNA Methylation Biomarkers in Buccal and Monocyte Cells for Female Rheumatoid Arthritis

Gary Craig[1], Howard Kenney[1], Eric E. Nilsson[2], Ingrid Sadler-Riggleman[2], Daniel Beck[2], and Michael K. Skinner[2] *

SUPPLEMENTAL MATERIAL

SUPPLEMENTAL METHODS

Clinical sample collection and analysis

Two independent single centers (Arthritis Northwest, ANW, Spokane, WA, USA and Dx Biosamples, LLC in San Diego, California, USA) performed the prospective and open clinical study. The participant approval and informed consent was obtained from all participants prior to the clinical sample collection. The study was approved by the Quorum Review Institutional Review Board (IRB) for the ANW Clinic, Spokane, WA with code # AE010831, and Investigational Review Board (www.dxirb.com) of Dx Biosamples, LLC, San Diego, CA, USA, with code IORG # 0006584, and IRB # 00007904. Reporting of information to the participants was not allowed within the IRBs. All research was performed in accordance with relevant guidelines/regulations. The arthritis severity was assessed for the Caucasian population with diagnostic rheumatoid factor autoantibody (RF) positive assay and cyclic citrullinated peptide autoantibody (CCP) positive assay. The arthritis severity was also assessed for both the Caucasian and African American populations with a more qualitative Clinical Disease Activity Index (CDAI) analysis with a high activity score of >22.1-76.0 and moderate activity score of 10.1-22.0. The CDAI was also supported with DAS28 and RAPID3 assays for the Caucasian population. We included three group comparisons (buccal from female with RA arthritis (case) or without (control)) for both Caucasian and African American populations. The females included Caucasians and African Americans with a mean group age of 54 years. Buccal samples were frozen and stored (-20 °C) for the subsequent epigenetic analysis. Blood was collected into BD Vacutainer CPT tubes (Becton, Dickinson and Company, Franklin Lakes, NJ) by ANW Spokane, WA and

shipped overnight on ice for immediate processing. Monocytes were isolated using antibody-linked magnetic beads (Dynabeads CD14, Life Technologies # 11149D).

Cell Isolation and DNA Preparation –

Human buccal samples were kept frozen and thawed for analysis. Blood samples were kept refrigerated before monocyte isolation. For monocyte isolation the blood was diluted 1:2 in Isolation Buffer (1X PBS, 0.1% BSA, 2mM EDTA) then centrifuged at 600 x *g* for 10 min at room temperature in a swinging bucket rotor. The plasma upper layer was discarded, and the blood resuspended to the original volume in Isolation Buffer. Magnetic beads (Dynabeads CD14, Life Technologies # 11149D) were added to the blood samples (25 µL of pre-washed beads for 1 ml of blood sample). The mixture (beads-cells) was incubated for 20 min between 2 °C and 8 °C with gentle tilting and rotation. The mixture was then placed on a magnet for 2 minutes and the supernatant carefully removed. One ml of Isolation Buffer was added to the beads-cells mixture then homogenized and incubated 2 minutes on the magnet. This step was repeated at least 3 times. The bead-bound cells were resuspended in 100 µl 1X PBS pH 7.4 and genomic DNA isolation was performed.

Genomic DNA from buccal swabs or monocytes was prepared as follows: The buccal swabs were incubated with 750 µl of cell lysis solution (100 mM NaCl, 10 mM Tris, 25 mM EDTA, 0.5% SDS) and 3.5 µl of Proteinase K (20 mg/ml) for 3 hours in a heating block at 55 °C. The lysis solution was transferred to a new tube and spun for 2 min at full speed. For the monocytes, 820 µl of DNA extraction buffer (50 mM Tris pH 8, 10 mM EDTA pH 8, 0.5% SDS) and 80 µl Proteinase K (20 mg/ml) were added to the

bead-bound monocytes and the sample incubated at 55 °C for 2 hours under constant rotation. The samples were placed on a magnet where the magnetic beads were discarded and the supernatants containing the DNA kept. Then for both the buccal swabs and the monocytes, 300 µl of protein precipitation solution (Promega Genomic DNA Purification Kit, A795A, Madison, WI) was added, the sample was mixed and incubated on ice for 15 min, then spun at 4 °C at 13,500 x *g* for 20 min. The supernatant was transferred to a fresh tube, then precipitated over night at -20 °C with 1 mL of cold 100% isopropanol and 2 µl glycoblue. The sample was then centrifuged at 4 °C, 13,500 x *g* for 20 min. The supernatant was discarded and the pellet washed with 70% cold ethanol then returned to freezer for 20-30 min. The sample was then centrifuged at 4 °C, 13,500 x *g* for 10 min. The supernatant was discarded, and the pellet air-dried for 5 min then resuspended in 100 µl nuclease free water. DNA concentration was measured using the Nanodrop (Thermo Fisher, Waltham, MA).

Methylated DNA Immunoprecipitation (MeDIP) –

Methylated DNA Immunoprecipitation (MeDIP) with genomic DNA was performed as follows: individual genomic DNA samples (2-4 ug of total DNA) were diluted to 130 µl with 1X Tris-EDTA (TE, 10 mM Tris, 1 mM EDTA) and sonicated with the Covaris M220 using the 300 bp setting. Fragment size was verified on a 2% E-gel agarose gel. The sonicated DNA was transferred from the Covaris tube to a 1.7 ml microfuge tube, and the volume was measured. The sonicated DNA was then diluted with TE buffer (10 mM Tris HCl, pH 7.5; 1 mM EDTA) to 400 µl, heat-denatured for 10 min at 95 °C, then immediately cooled on ice for 10 min. Then 100 µl of 5X IP buffer and 5 µg of antibody

(monoclonal mouse anti 5-methyl cytidine; Diagenode #C15200006) were added to the denatured sonicated DNA. The DNA-antibody mixture was incubated overnight on a rotator at 4 °C. The following day magnetic beads (Dynabeads M-280 Sheep anti-Mouse IgG; 11201D) were pre-washed as follows: The beads were resuspended in the vial, then the appropriate volume (50 µl per sample) was transferred to a microfuge tube. The same volume of Washing Buffer (at least 1 ml 1X PBS with 0.1% BSA and 2 mM EDTA) was added and the bead sample was resuspended. The tube was then placed into a magnetic rack for 1-2 min and the supernatant was discarded. The tube was removed from the magnetic rack and the beads were washed once. The washed beads were resuspended in the same volume of 1X IP buffer (50 mM sodium phosphate pH 7.0, 700 mM NaCl, 0.25% Triton X-100) as the initial volume of beads. Beads (50 µl) were added to the 500 µl of DNA-antibody mixture from the overnight incubation, then incubated for 2 hours on a rotator at 4 °C. After the incubation, the bead-antibody-DNA complex was washed three times with 1X IP buffer as follows: The tube was placed into a magnetic rack for 1-2 min and the supernatant was discarded, then the magnetic bead antibody pellet was washed with 1X IP buffer 3 times. The washed bead antibody DNA pellet was then resuspended in 250 µl digestion buffer with 3.5 µl Proteinase K (20 mg/ml). The sample was incubated for 2-3 hours on a rotator at 55 °C, then 250 µl of buffered Phenol-Chloroform-Isoamylalcohol solution was added to the sample, and the tube was vortexed for 30 sec and then centrifuged at 14,000 x *g* for 5 min at room temperature. The aqueous supernatant was carefully removed and transferred to a fresh microfuge tube. Then 250 µl chloroform were added to the supernatant from the previous step, vortexed for 30 sec and centrifuged at 13,500 x *g* for 5 min at room

temperature. The aqueous supernatant was removed and transferred to a fresh microfuge tube. To the supernatant 2 µl of glycoblue (20 mg/ml), 20 µl of 5M NaCl and 500 µl ethanol were added and mixed well, then precipitated in -20 °C freezer for 1 hour to overnight. The precipitate was centrifuged at 13,500 x *g* for 20 min at 4 °C and the supernatant was removed, while not disturbing the pellet. The pellet was washed with 500 µl cold 70% ethanol in -20 °C freezer for 15 min then centrifuged again at 13,500 x *g* for 5 min at 4 °C and the supernatant was discarded. The tube was spun again briefly to collect residual ethanol to the bottom of the tube and as much liquid as possible was removed with gel loading tip. The pellet was air-dried at RT until it looked dry (about 5 min) then resuspended in 20 µl H_2O or TE. DNA concentration was measured in Qubit (Life Technologies) with ssDNA kit (Molecular Probes Q10212).

MeDIP-Seq Analysis –

The MeDIP DNA samples (50 ng of each) were used to create libraries for next generation sequencing (NGS) using the NEBNext Ultra RNA Library Prep Kit for Illumina (San Diego, CA) starting at step 1.4 of the manufacturer's protocol to generate double stranded DNA. After this step the manufacturer's protocol was followed. Each sample received a separate index primer. NGS was performed at WSU Spokane Genomics Core using the Illumina HiSeq 2500 with a PE50 application, with a read size of approximately 50 bp and approximately 25 (10-50 range) million reads per sample, and 6-7 sample libraries each were run in one lane.

Molecular Bioinformatics and Statistics –

Basic read quality was verified using information produced by the FastQC program [1]. Reads were filtered and trimmed to remove low quality base pairs using Trimmomatic [2]. The reads for each sample were mapped to the GRCh38 human genome using Bowtie2 [3] with default parameter options. The mapped read files were then converted to sorted BAM files using SAMtools [4]. To identify DMR, the reference genome was broken into 1000 bp windows. The MEDIPS R package [5] was used to calculate differential coverage between control and exposure sample groups. The EdgeR p-value [6] was used to determine the relative difference between the two groups for each genomic window. Windows with an EdgeR p-value less than 10^{-4} were considered DMRs. The DMR edges were extended until no genomic window with an EdgeR p-value less than 0.1 remained within 1000 bp of the DMR. CpG density and other information was then calculated for the DMR based on the reference genome. DMR were annotated using the biomaRt R package [7] to access the Ensembl database [8]. The genes that overlapped with DMR were then input into the KEGG pathway search [9,10] to identify associated pathways. The DMR associated genes were then sorted into functional groups using information provided by the DAVID [11] and Panther [12] databases incorporated into an internal curated database (www.skinner.wsu.edu under genomic data). All MeDIP-Seq genomic data obtained in the current study have been deposited in the NCBI public GEO database (GEO #: GSE186179).

SUPPLEMENTAL REFERENCES

1 Andrews, S. *FastQC: a quality control tool for high throughput sequence data.* , <https://www.bioinformatics.babraham.ac.uk/projects/fastqc/> (2010).

2 Bolger, A. M., Lohse, M. & Usadel, B. Trimmomatic: a flexible trimmer for Illumina sequence data. *Bioinformatics* **30**, 2114-2120, doi:10.1093/bioinformatics/btu170 (2014).

3 Langmead, B. & Salzberg, S. L. Fast gapped-read alignment with Bowtie 2. *Nature methods* **9**, 357-359, doi:10.1038/nmeth.1923 (2012).

4 Li, H. *et al.* The Sequence Alignment/Map format and SAMtools. *Bioinformatics* **25**, 2078-2079, doi:10.1093/bioinformatics/btp352 (2009).

5 Lienhard, M., Grimm, C., Morkel, M., Herwig, R. & Chavez, L. MEDIPS: genome-wide differential coverage analysis of sequencing data derived from DNA enrichment experiments. *Bioinformatics* **30**, 284-286, doi:10.1093/bioinformatics/btt650 (2014).

6 Robinson, M. D., McCarthy, D. J. & Smyth, G. K. edgeR: a Bioconductor package for differential expression analysis of digital gene expression data. *Bioinformatics* **26**, 139-140, doi:10.1093/bioinformatics/btp616 (2010).

7 Durinck, S., Spellman, P. T., Birney, E. & Huber, W. Mapping identifiers for the integration of genomic datasets with the R/Bioconductor package biomaRt. *Nature protocols* **4**, 1184-1191, doi:10.1038/nprot.2009.97 (2009).

8 Cunningham, F. *et al.* Ensembl 2015. *Nucleic acids research* **43**, D662-669, doi:10.1093/nar/gku1010 (2015).

9 Kanehisa, M. & Goto, S. KEGG: kyoto encyclopedia of genes and genomes. *Nucleic acids research* **28**, 27-30, doi:gkd027 [pii] (2000).

10 Kanehisa, M. *et al.* Data, information, knowledge and principle: back to metabolism in KEGG. *Nucleic acids research* **42**, D199-205, doi:10.1093/nar/gkt1076 (2014).

11 Huang da, W., Sherman, B. T. & Lempicki, R. A. Systematic and integrative analysis of large gene lists using DAVID bioinformatics resources. *Nature protocols* **4**, 44-57, doi:10.1038/nprot.2008.211 (2009).

12 Mi, H., Muruganujan, A., Casagrande, J. T. & Thomas, P. D. Large-scale gene function analysis with the PANTHER classification system. *Nature protocols* **8**, 1551-1566, doi:10.1038/nprot.2013.092 (2013).

Supplemental Figure and Table Legends

Supplemental Figure S1. DMR CpG density for CpG / 100 bp and length (bp) are presented for **(A)** Caucasian control versus RA buccal DMRs. **(B)** Caucasian control versus RA buccal DMRs. **(C)** Caucasian control versus RA monocyte DMRs. **(D)** Caucasian control versus RA monocyte DMRs. **(E)** AA control versus AA RA buccal DMRs. **(F)** A control versus AA RA monocyte DMRs. **(G)** AA and CC buccal combined control and AA and CC buccal combined RA DMRs. **(H)** AA and CC all buccal combined controls versus AA and CC combined RA buccal DMRs.

Supplemental Table S1. DMR lists at p<1e-04 Caucasian control versus RA buccal, with presentation of name, chromosomal location, DMR start and stop nucleotide number for chromosome, length (bp), number of 1 kb significant windows, minimum p-value, CpG number and density, DMR maximum, log-fold change (maxLFC) (+ increase DNA methylation and (-) decrease DNA methylation), and gene association within 10 kb and gene functional categories. DMR statistics for EdgeR minimum p-value and FDR minimum p-value presented.

Supplemental Table S2. DMR lists at p<1e-04 Caucasian control versus RA monocyte, with presentation of name, chromosomal location, DMR start and stop nucleotide number for chromosome, length (bp), number of 1 kb significant windows, minimum p-value, CpG number and density, DMR maximum, log-fold change (maxLFC) (+ increase

DNA methylation and (-) decrease DNA methylation), and gene association within 10 kb and gene functional categories. DMR statistics for EdgeR minimum p-value and FDR minimum p-value presented.

Supplemental Table S3. DMR lists at p<1e-04 African American control versus AA RA, with presentation of name, chromosomal location, DMR start and stop nucleotide number for chromosome, length (bp), number of 1 kb significant windows, minimum p-value, CpG number and density, DMR maximum, log-fold change (maxLFC) (+ increase DNA methylation and (-) decrease DNA methylation), and gene association within 10 kb and gene functional categories. DMR statistics for EdgeR minimum p-value and FDR minimum p-value presented.

Supplemental Table S4. Combination African American and Caucasian control versus combined African American and Caucasian (all buccal), with presentation of name, chromosomal location, DMR start and stop nucleotide number for chromosome, length (bp), number of 1 kb significant windows, minimum p-value, CpG number and density, DMR maximum, log-fold change (maxLFC) (+ increase DNA methylation and (-) decrease DNA methylation), and gene association within 10 kb and gene functional categories. DMR statistics for EdgeR minimum p-value and FDR minimum p-value presented.

Supplemental Table S5. DMR associated RA gene cell processes. The cell process name, total number neighbors, gene set seed, overlapping DMR associated genes, percent overlap, total list overlapping genes, Jaccard similarity, and p-value statistic for the overlapping gene set are presented.
