# Supplementary material for: Epigenome association study for DNA methylation biomarkers in buccal and monocyte cells for female rheumatoid arthritis
Source: Sci Rep. 2021 Dec 10;11:23789. doi: 10.1038/s41598-021-03170-6 (PMC8664902; doi:10.1038/s41598-021-03170-6)

Supplemental Figure S1

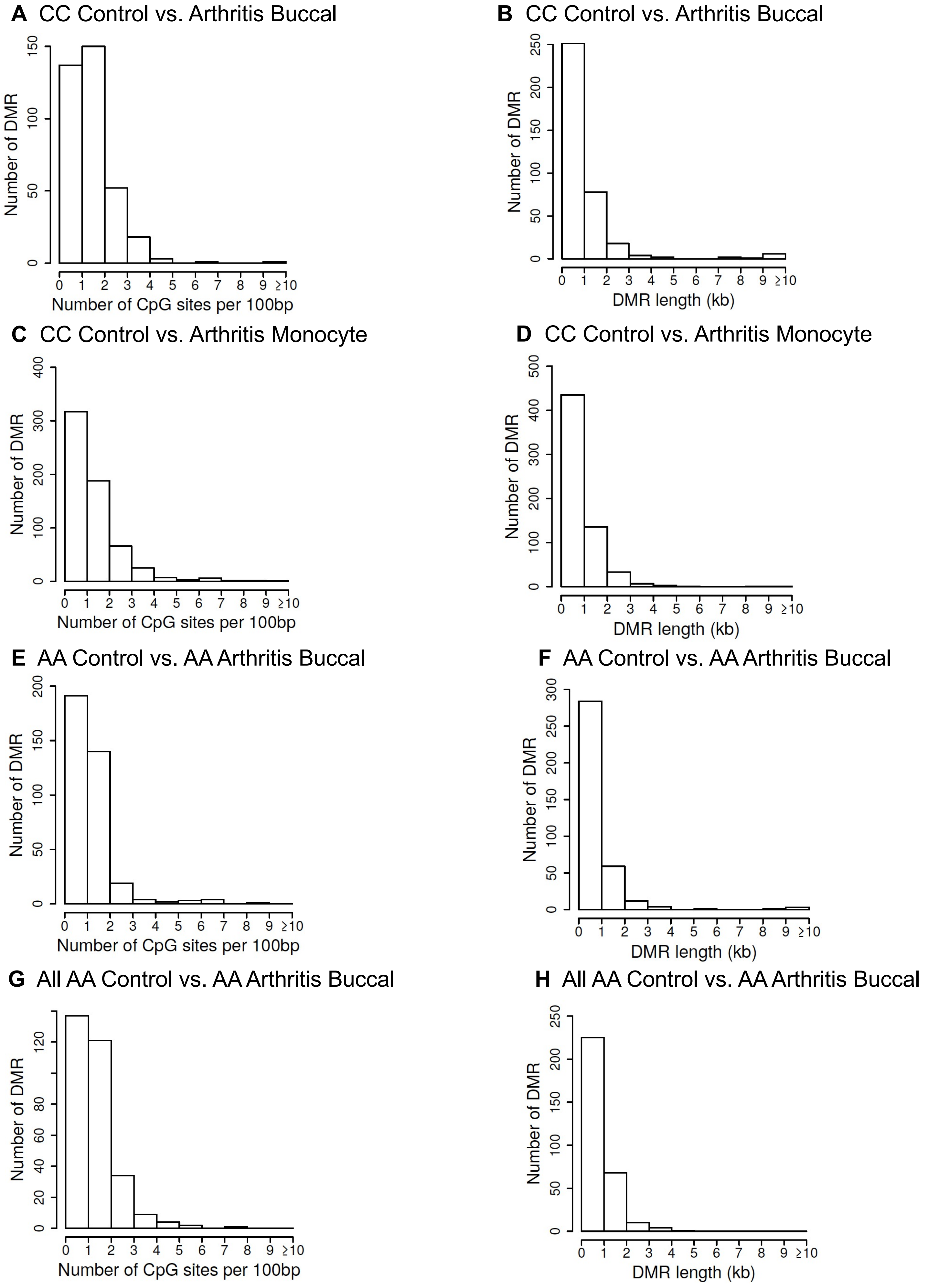

Supplement: Supplementary file 2 — Supplementary Figure S1. [file 41598_2021_3170_MOESM2_ESM.pdf]
