# Supplementary material for: Epigenome association study for DNA methylation biomarkers in buccal and monocyte cells for female rheumatoid arthritis
Source: Sci Rep. 2021 Dec 10;11:23789. doi: 10.1038/s41598-021-03170-6 (PMC8664902; doi:10.1038/s41598-021-03170-6)
Supplement: Supplementary file 3 — Supplementary Table S1. [file 41598_2021_3170_MOESM3_ESM.pdf]

Supplemental Table S1
DMR Table RA CC Buccal 1e-04

DMR Name	Chr	Start	Stop	Length	# Sig Win	minP	Min FDR	maxLFC	CpG #	CpG Density	Gene Annotation	Gene Category
DMR1:2392001	1	2392001	2395000	3000	1	5.07E-05	0.268	-0.7519786	109	3.633	MORN1;RER1;PEX10	Signaling;Unknown
DMR1:2683001	1	2683001	2700000	17000	1	6.28E-07	0.102	-0.937781	405	2.382	TTC34	
DMR1:11520001	1	11520001	11523000	3000	1	4.64E-05	0.257	-0.5187497	54	1.8	DISP3	
DMR1:17685001	1	17685001	17686000	1000	1	2.46E-05	0.212	-0.7350106	13	1.3	ARHGEF10L	Protease
DMR1:18981001	1	18981001	18983000	2000	1	7.71E-05	0.283	0.720297	4	0.2		
DMR1:20302001	1	20302001	20304000	2000	1	8.21E-05	0.288	-0.6493041	20	1	VWA5B1;AL020998.1	Development
DMR1:23506001	1	23506001	23508000	2000	1	4.17E-05	0.249	-1.1948979	31	1.55	E2F2	Transcription
DMR1:44211001	1	44211001	44212000	1000	1	4.60E-05	0.257	-0.8424165	4	0.4	DMAP1;ERI3	Epigenetic;Transcription
DMR1:54819001	1	54819001	54821000	2000	1	6.44E-05	0.276	0.8247328	14	0.7	LEXM	
DMR1:61025001	1	61025001	61026000	1000	1	5.43E-05	0.268	0.5177758	8	0.8	NFIA;NFIA-AS2	Transcription
DMR1:63574001	1	63574001	63575000	1000	1	3.24E-05	0.233	-0.7291712	13	1.3	ITGB3BP;EFCAB7	Receptor;Metabolism
DMR1:69306001	1	69306001	69307000	1000	1	4.00E-05	0.247	-0.6315142	13	1.3		
DMR1:98933001	1	98933001	98934000	1000	1	1.76E-05	0.198	0.6184726	15	1.5	PLPPR5	
DMR1:100630001	1	100630001	100631000	1000	1	7.87E-05	0.284	0.782343	8	0.8	LINC01349;AC099670.3	
DMR1:110491001	1	110491001	110492000	1000	1	6.80E-05	0.276	-0.6528995	15	1.5	AL358215.3;CYMP;CYMP-AS1	
DMR1:115286001	1	115286001	115288000	2000	1	8.09E-07	0.102	-0.8762112	53	2.65	AL049825.1;NGF-AS1;NGF	Signaling
DMR1:124288001	1	124288001	124289000	1000	1	8.18E-05	0.288	1.1508355	22	2.2		
DMR1:155529001	1	155529001	155531000	2000	1	3.77E-05	0.241	0.7005294	20	1	ASH1L	Transcription
DMR1:178712001	1	178712001	178714000	2000	1	2.38E-05	0.212	0.7349144	9	0.45		
DMR1:180307001	1	180307001	180308000	1000	1	2.36E-06	0.117	0.7490511	8	0.8	ACBD6	Metabolism
DMR1:196195001	1	196195001	196196000	1000	1	6.18E-05	0.276	0.5774487	3	0.3		
DMR1:200107001	1	200107001	200108000	1000	1	4.40E-05	0.254	0.7393096	17	1.7	NR5A2	Receptor
DMR1:204275001	1	204275001	204276000	1000	1	8.00E-06	0.157	-0.6186251	12	1.2	PLEKHA6;AL592114.3	Unknown
DMR1:213426001	1	213426001	213427000	1000	1	2.12E-05	0.212	-0.6732454	12	1.2	RPL31P13	
DMR1:220057001	1	220057001	220058000	1000	1	6.63E-05	0.276	0.623377	17	1.7	BPNT1	Transcription
DMR1:227732001	1	227732001	227733000	1000	1	1.36E-05	0.185	-1.0992018	30	3	SNAP47;JMJD4	Cytoskeleton;Receptor
DMR1:230263001	1	230263001	230264000	1000	1	6.91E-05	0.276	-0.7935973	28	2.8	GALNT2;AL136988.2	Metabolism
DMR2:4447001	2	4447001	4448000	1000	1	9.61E-05	0.297	-0.9253999	10	1		
DMR2:10257001	2	10257001	10258000	1000	1	7.12E-05	0.279	-0.6211783	23	2.3		
DMR2:26844001	2	26844001	26846000	2000	1	2.37E-06	0.117	0.7190883	34	1.7	DPYSL5	Metabolism
DMR2:36974001	2	36974001	36975000	1000	1	7.49E-05	0.282	0.7089818	26	2.6	STRN;HEATR5B	Receptor
DMR2:85069001	2	85069001	85070000	1000	1	7.32E-05	0.282	0.6137615	17	1.7	KCMF1;LINC01964;AC078974.1	
DMR2:121796001	2	121796001	121797000	1000	1	7.45E-05	0.282	-0.7107261	15	1.5	LINC01823	
DMR2:136872001	2	136872001	136873000	1000	1	1.99E-05	0.209	0.7372966	8	0.8	THSD7B	Extracellular Matrix
DMR2:139975001	2	139975001	139976000	1000	1	2.69E-05	0.222	0.9861922	1	0.1	RN7SL283P	
DMR2:150249001	2	150249001	150251000	2000	1	5.08E-05	0.268	0.6865429	18	0.9	LINC01818;LINC01817	
DMR2:156263001	2	156263001	156264000	1000	1	9.22E-05	0.297	-0.6652247	6	0.6	LINC01876	
DMR2:173320001	2	173320001	173321000	1000	1	1.85E-05	0.203	0.7095593	11	1.1		
DMR2:219480001	2	219480001	219481000	1000	1	8.81E-05	0.297	-0.9069257	11	1.1	SPEG;ASIC4-AS1	
DMR2:220823001	2	220823001	220824000	1000	1	4.89E-05	0.261	0.7076744	18	1.8	AC093843.1;AC093843.2	
DMR2:230931001	2	230931001	230932000	1000	1	1.63E-05	0.188	-0.5832339	23	2.3	GPR55;AC012507.3	Receptor
DMR2:232263001	2	232263001	232264000	1000	1	7.45E-06	0.157	-0.8707451	23	2.3	DIS3L2	Transcription
DMR2:233287001	2	233287001	233288000	1000	1	3.53E-05	0.24	0.9146456	9	0.9	ATG16L1;SCARNA6	Unknown
DMR2:236127001	2	236127001	236128000	1000	1	6.80E-05	0.276	-0.8110873	19	1.9	AGAP1	Signaling
DMR2:239989001	2	239989001	239990000	1000	1	4.20E-05	0.249	-0.670395	14	1.4	NDUFA10	Metabolism
DMR3:2125001	3	2125001	2126000	1000	1	5.87E-05	0.273	0.6956516	14	1.4	CNTN4;CNTN4-AS2;AC087427.1	Cytoskeleton
DMR3:16235001	3	16235001	16236000	1000	1	5.90E-05	0.273	-0.672694	11	1.1	GALNT15	Metabolism
DMR3:41647001	3	41647001	41648000	1000	1	4.01E-05	0.247	-0.6179633	23	2.3	ULK4	
DMR3:54242001	3	54242001	54243000	1000	1	1.42E-05	0.185	-0.7268623	16	1.6	CACNA2D3	Transport
DMR3:76803001	3	76803001	76804000	1000	1	1.53E-05	0.188	0.6200346	10	1	ROBO2	Receptor
DMR3:116116001	3	116116001	116117000	1000	1	1.01E-05	0.162	0.8339338	15	1.5	LSAMP	Extracellular Matrix
DMR3:126390001	3	126390001	126391000	1000	1	8.71E-05	0.296	-0.5360294	29	2.9	CCDC37-DT;CFAP100	
DMR3:146754001	3	146754001	146755000	1000	1	5.12E-05	0.268	0.6330083	6	0.6		
DMR3:149101001	3	149101001	149104000	3000	1	6.36E-05	0.276	0.7340604	30	1	HLTF-AS1;Y_RNA;MED28P2	
DMR3:154088001	3	154088001	154090000	2000	1	5.29E-05	0.268	-0.6431693	55	2.75	ARHGEF26-AS1	
DMR3:170270001	3	170270001	170271000	1000	1	9.49E-05	0.297	-0.7733442	13	1.3	PRKCI	Signaling
DMR3:173478001	3	173478001	173479000	1000	1	7.33E-06	0.157	1.012265	4	0.4	NLGN1	Signaling
DMR3:173814001	3	173814001	173816000	2000	1	3.61E-05	0.241	0.7112116	24	1.2	NLGN1	Signaling
DMR3:184712001	3	184712001	184714000	2000	1	4.71E-05	0.257	0.845434	42	2.1	MAGEF1;AC107294.2	
DMR3:189949001	3	189949001	189950000	1000	1	1.08E-05	0.162	0.8120709	8	0.8	P3H2	
DMR3:194297001	3	194297001	194299000	2000	1	4.50E-07	0.101	-1.0544994	78	3.9	LINC02048;LINC00887;AC117469.1	

DMR3:195289001	3	195289001	195290000	1000	1	8.62E-05	0.295	0.6159412	17	1.7	ACAP2;ACAP2-IT1	Transcription
DMR4:7161001	4	7161001	7164000	3000	1	9.85E-05	0.301	-0.6196606	64	2.133		
DMR4:9225001	4	9225001	9238000	13000	1	1.33E-05	0.185	-0.63487	408	3.138	AC108519.1;USP17L11;USP17L12;USP17L13;USP17L14P;USP17L15;USP17L16P;USP17L17	Protease
DMR4:9239001	4	9239001	9248000	9000	1	6.67E-05	0.276	-0.7629088	279	3.1	AC108519.1;USP17L14P;USP17L15;USP17L16P;USP17L17;USP17L18;USP17L19	Protease
DMR4:9254001	4	9254001	9273000	19000	2	4.75E-05	0.257	-0.6725356	605	3.184	AC108519.1;USP17L17;USP17L18;USP17L19;USP17L20;USP17L21;USP17L22;USP17L23	Protease
DMR4:9323001	4	9323001	9370000	47000	2	4.16E-06	0.133	-0.7534909	1482	3.153	AC108519.1;USP17L24;USP17L25;USP17L26;USP17L5;USP17L27;USP17L28;USP17L29;USP17L9P;USP17L30;USP17L6P	Protease
DMR4:28540001	4	28540001	28541000	1000	1	3.46E-06	0.129	0.6829793	0	0	AC097480.1	
DMR4:43994001	4	43994001	43995000	1000	1	9.41E-05	0.297	0.6857949	11	1.1	AC114757.1	
DMR4:53731001	4	53731001	53732000	1000	1	3.88E-05	0.245	-0.7421856	13	1.3	AC058822.1;AC124017.1	
DMR4:58432001	4	58432001	58433000	1000	1	2.15E-05	0.212	0.7846277	12	1.2		
DMR4:64581001	4	64581001	64582000	1000	1	1.43E-05	0.185	0.8185	11	1.1		
DMR4:71175001	4	71175001	71176000	1000	1	1.66E-06	0.11	-0.9083772	21	2.1	SLC4A4	Transport
DMR4:82209001	4	82209001	82211000	2000	1	6.28E-05	0.276	0.7738565	27	1.35		
DMR4:82824001	4	82824001	82825000	1000	1	9.34E-05	0.297	0.7798957	12	1.2	SEC31A	Transport
DMR4:101799001	4	101799001	101800000	1000	1	6.86E-05	0.276	0.8910423	13	1.3	BANK1	Development
DMR4:139164001	4	139164001	139165000	1000	1	9.94E-05	0.301	0.4910118	10	1	ELF2;Metazoa_SRP	Transcription
DMR4:162380001	4	162380001	162381000	1000	1	9.34E-05	0.297	0.7794223	10	1		
DMR4:171745001	4	171745001	171746000	1000	1	5.35E-05	0.268	0.97484	7	0.7		
DMR5:1031001	5	1031001	1032000	1000	1	9.25E-05	0.297	-0.9375841	26	2.6	NKD2	
DMR5:1400001	5	1400001	1402000	2000	1	9.20E-05	0.297	-0.9093993	58	2.9	SLC6A3	Transport
DMR5:5267001	5	5267001	5268000	1000	1	2.46E-05	0.212	-0.7411193	29	2.9	ADAMTS16	Protease
DMR5:8784001	5	8784001	8786000	2000	1	5.50E-05	0.268	0.4754778	7	0.35	AC091932.1;AC091932.3	
DMR5:14558001	5	14558001	14559000	1000	1	3.61E-05	0.241	0.9677786	10	1		
DMR5:18782001	5	18782001	18783000	1000	1	3.97E-05	0.247	0.9394629	8	0.8		
DMR5:19549001	5	19549001	19550000	1000	1	5.41E-05	0.268	0.6846499	3	0.3	CDH18	Cytoskeleton
DMR5:27835001	5	27835001	27836000	1000	1	1.98E-08	0.022	0.7368629	18	1.8		
DMR5:30186001	5	30186001	30187000	1000	1	9.34E-05	0.297	0.7308722	3	0.3		
DMR5:31357001	5	31357001	31358000	1000	1	7.86E-05	0.284	-0.7286543	19	1.9		
DMR5:36651001	5	36651001	36652000	1000	1	6.66E-05	0.276	0.6702785	4	0.4	SLC1A3;AC008957.2	Metabolism
DMR5:47715001	5	47715001	47716000	1000	1	2.74E-05	0.222	1.0146262	20	2		
DMR5:72925001	5	72925001	72927000	2000	1	7.06E-06	0.157	-0.8123816	57	2.85	TNPO1	Metabolism
DMR5:79492001	5	79492001	79493000	1000	1	4.66E-05	0.257	-0.8797911	12	1.2	HOMER1	Signaling
DMR5:122870001	5	122870001	122871000	1000	1	2.53E-05	0.213	-0.6663753	9	0.9	SNX24	
DMR5:147702001	5	147702001	147703000	1000	1	7.77E-06	0.157	0.5885004	2	0.2	JAKMIP2	
DMR5:155738001	5	155738001	155740000	2000	1	4.03E-05	0.247	0.3431794	7	0.35		
DMR5:162690001	5	162690001	162691000	1000	1	9.77E-05	0.3	0.776897	13	1.3	AC113414.1	
DMR5:168831001	5	168831001	168832000	1000	1	7.20E-05	0.282	-0.5737609	17	1.7	SLIT3	Development
DMR5:173081001	5	173081001	173083000	2000	1	8.14E-05	0.288	-0.7600884	34	1.7	CREBRF;CDC42P5	
DMR6:3872001	6	3872001	3873000	1000	1	9.60E-05	0.297	-0.6971246	25	2.5	AL391422.2	
DMR6:16290001	6	16290001	16291000	1000	1	4.97E-05	0.265	-0.6497151	18	1.8	GMPR;ATXN1	Metabolism;Transcription
DMR6:25110001	6	25110001	25111000	1000	1	3.24E-05	0.233	-0.8203943	6	0.6	CMAHP;AL133268.4	
DMR6:33414001	6	33414001	33417000	3000	1	1.35E-05	0.185	-0.559841	47	1.567	KIFC1;PHF1;CUTA;SYNGAP1	Cytoskeleton;Epigenetic;Signaling
DMR6:39855001	6	39855001	39857000	2000	1	4.77E-05	0.257	-0.6754721	24	1.2	DAAM2	Cytoskeleton
DMR6:40156001	6	40156001	40157000	1000	1	5.46E-05	0.268	-0.648341	8	0.8		
DMR6:48705001	6	48705001	48706000	1000	1	4.85E-05	0.26	0.5554156	2	0.2	FO393412.1	
DMR6:106946001	6	106946001	106947000	1000	1	6.71E-05	0.276	0.5954986	12	1.2		
DMR6:114971001	6	114971001	114972000	1000	1	5.40E-06	0.148	-0.9856488	7	0.7	AL590550.1	
DMR6:125239001	6	125239001	125240000	1000	1	7.03E-05	0.278	-0.9612325	6	0.6	TPD52L1;HDDC2	
DMR6:130261001	6	130261001	130262000	1000	1	1.43E-05	0.185	-0.8591036	14	1.4	SAMD3	Signaling
DMR6:132462001	6	132462001	132463000	1000	1	7.68E-06	0.157	0.8663081	8	0.8	STX7	Transcription
DMR6:143194001	6	143194001	143195000	1000	1	1.28E-05	0.182	-0.7839042	15	1.5	AIG1	
DMR6:152811001	6	152811001	152813000	2000	1	6.31E-05	0.276	0.5289729	10	0.5	LINC02840	
DMR6:153234001	6	153234001	153235000	1000	1	7.98E-06	0.157	0.6782797	7	0.7	AL590867.1	
DMR6:153305001	6	153305001	153306000	1000	1	3.15E-05	0.233	0.6515728	5	0.5	AL590867.1;AL358134.1	
DMR6:157178001	6	157178001	157179000	1000	1	2.23E-05	0.212	-0.8080044	20	2	ARID1B	Transcription
DMR6:157612001	6	157612001	157613000	1000	1	7.36E-05	0.282	-0.6928543	14	1.4	ZDHHC14	Unknown
DMR6:162111001	6	162111001	162112000	1000	1	2.66E-05	0.221	0.6358878	23	2.3	PRKN	

DMR6:166183001	6	166183001	166185000	2000	1	8.21E-05	0.288	-0.6148261	26	1.3		
DMR6:166639001	6	166639001	166640000	1000	1	3.57E-05	0.241	-0.5797635	21	2.1	RPS6KA2	
DMR6:166733001	6	166733001	166734000	1000	1	3.18E-05	0.233	-0.6129514	18	1.8	RPS6KA2	
DMR6:170466001	6	170466001	170468000	2000	1	4.26E-05	0.252	-0.3742239	14	0.7		
DMR7:4543001	7	4543001	4545000	2000	1	6.53E-05	0.276	0.8385193	22	1.1		
DMR7:29069001	7	29069001	29070000	1000	1	8.24E-05	0.288	0.7421796	15	1.5	CPVL	Protease
DMR7:43059001	7	43059001	43060000	1000	1	9.65E-05	0.297	-0.8228739	36	3.6	AC005537.1	
DMR7:47165001	7	47165001	47166000	1000	1	5.54E-05	0.268	0.6070267	15	1.5		
DMR7:54492001	7	54492001	54494000	2000	1	6.64E-05	0.276	0.8505341	20	1		
DMR7:77463001	7	77463001	77464000	1000	1	9.01E-05	0.297	0.82035	11	1.1	GCNT1P5	
DMR7:78466001	7	78466001	78467000	1000	1	7.99E-05	0.287	-0.6250665	9	0.9	MAGI2	Metabolism
DMR7:82036001	7	82036001	82037000	1000	1	5.40E-05	0.268	0.6922018	5	0.5	CACNA2D1;CACNA2D1-AS1	Metabolism
DMR7:91617001	7	91617001	91619000	2000	1	1.97E-05	0.209	0.9932357	21	1.05		
DMR7:101088001	7	101088001	101090000	2000	1	2.30E-05	0.212	-0.6563952	94	4.7	TRIM56	Metabolism
DMR7:109050001	7	109050001	109051000	1000	1	7.71E-05	0.283	0.5421244	4	0.4		
DMR7:116626001	7	116626001	116627000	1000	1	9.14E-05	0.297	0.6414961	10	1	COMETT	
DMR7:128089001	7	128089001	128090000	1000	1	2.11E-05	0.212	-0.655339	22	2.2	SND1;MIR593	Transcription
DMR7:130374001	7	130374001	130375000	1000	1	7.45E-05	0.282	-0.7860485	17	1.7	CPA5;CPA1	Protease
DMR7:150978001	7	150978001	150979000	1000	1	1.42E-06	0.11	1.8140494	101	10.1	KCNH2	
DMR8:2482001	8	2482001	2485000	3000	1	5.53E-05	0.268	-0.7810081	63	2.1	AC245519.1	
DMR8:4135001	8	4135001	4136000	1000	1	9.16E-06	0.158	1.2123793	3	0.3	CSMD1	Signaling
DMR8:5549001	8	5549001	5550000	1000	1	7.02E-05	0.278	-0.5781185	18	1.8		
DMR8:10182001	8	10182001	10183000	1000	1	3.67E-05	0.241	-0.8602191	4	0.4	MSRA	Metabolism
DMR8:11173001	8	11173001	11175000	2000	1	2.81E-05	0.223	-0.8237837	41	2.05	XKR6;AF131215.1	Immune
DMR8:13484001	8	13484001	13485000	1000	1	8.93E-05	0.297	0.7365642	8	0.8	DLC1	Signaling
DMR8:14756001	8	14756001	14758000	2000	1	4.72E-05	0.257	0.7387398	25	1.25	SGCZ	Cytoskeleton
DMR8:15449001	8	15449001	15450000	1000	1	8.34E-06	0.157	-0.8806365	12	1.2	TUSC3	Metabolism
DMR8:38188001	8	38188001	38189000	1000	1	9.92E-05	0.301	0.8107904	6	0.6	BAG4	Protein Binding
DMR8:42506001	8	42506001	42507000	1000	1	7.74E-05	0.283	-0.611471	20	2	SLC20A2	Metabolism
DMR8:47898001	8	47898001	47900000	2000	1	3.36E-06	0.129	0.7557558	29	1.45	PRKDC	Signaling
DMR8:49711001	8	49711001	49712000	1000	1	8.82E-06	0.157	0.7713347	17	1.7		
DMR8:54505001	8	54505001	54506000	1000	1	5.87E-05	0.273	0.729874	7	0.7	RP1	Receptor
DMR8:64044001	8	64044001	64045000	1000	1	1.95E-05	0.209	0.6825514	2	0.2	LINC01414	
DMR8:69840001	8	69840001	69841000	1000	1	6.12E-05	0.276	0.6220525	12	1.2	SLCO5A1;AC079089.1	Metabolism
DMR8:73605001	8	73605001	73606000	1000	1	7.09E-05	0.279	0.936455	9	0.9	STAU2	Transcription
DMR8:74422001	8	74422001	74423000	1000	1	3.88E-05	0.245	0.5381695	3	0.3	GDAP1	Signaling
DMR8:75569001	8	75569001	75570000	1000	1	2.38E-05	0.212	-0.9303842	9	0.9	HNF4G	Transcription
DMR8:95878001	8	95878001	95879000	1000	1	3.59E-06	0.129	0.6386625	4	0.4		
DMR8:100881001	8	100881001	100882000	1000	1	6.18E-05	0.276	-0.7307589	10	1		
DMR8:110351001	8	110351001	110352000	1000	1	5.38E-05	0.268	0.7280198	1	0.1	AC073023.1	
DMR8:116631001	8	116631001	116632000	1000	1	1.03E-05	0.162	-0.7788231	20	2		
DMR8:122573001	8	122573001	122574000	1000	1	4.09E-05	0.249	-0.610334	20	2	LINC01151	
DMR8:123059001	8	123059001	123060000	1000	1	6.17E-05	0.276	0.6071968	17	1.7	TBC1D31	
DMR8:123411001	8	123411001	123412000	1000	1	3.23E-05	0.233	-0.5795666	19	1.9	ATAD2;IMPDH1P6;NTAQ1	Metabolism
DMR8:142946001	8	142946001	142947000	1000	1	7.59E-05	0.282	-0.7509827	20	2		
DMR8:144598001	8	144598001	144599000	1000	1	4.76E-05	0.257	-0.8684843	32	3.2	ARHGAP39	
DMR9:36230001	9	36230001	36231000	1000	1	4.76E-05	0.257	0.5754555	16	1.6	CLTA;GNE	Transport;Signaling
DMR9:40661001	9	40661001	40663000	2000	1	7.45E-05	0.282	0.5176053	18	0.9		
DMR9:42678001	9	42678001	42681000	3000	1	6.40E-05	0.276	0.8187117	37	1.233	FGF7P4	
DMR9:63460001	9	63460001	63462000	2000	1	2.16E-05	0.212	0.6021715	17	0.85		
DMR9:67382001	9	67382001	67385000	3000	1	4.77E-06	0.144	0.672953	67	2.233		
DMR9:86223001	9	86223001	86226000	3000	1	1.19E-06	0.104	0.7511934	39	1.3	C9orf153;AL137849.1	
DMR9:86250001	9	86250001	86251000	1000	1	9.52E-05	0.297	-0.7394473	6	0.6	C9orf153;RN7SKP264	
DMR9:96031001	9	96031001	96034000	3000	1	6.72E-05	0.276	-0.6506345	49	1.633	ERCC6L2;LINC00092	
DMR9:96203001	9	96203001	96204000	1000	1	9.15E-05	0.297	-0.6216674	18	1.8		
DMR9:114905001	9	114905001	114906000	1000	1	6.89E-05	0.276	0.6994659	9	0.9	DELEC1;TNFSF8	
DMR9:117967001	9	117967001	117968000	1000	1	6.60E-05	0.276	0.6620306	8	0.8	AL160272.2	
DMR9:121076001	9	121076001	121077000	1000	1	5.39E-05	0.268	0.3428838	4	0.4	CNTRL	Unknown
DMR9:128232001	9	128232001	128234000	2000	1	9.19E-05	0.297	-0.6625876	48	2.4	DNM1	Cytoskeleton
DMR9:130202001	9	130202001	130207000	5000	1	5.70E-05	0.272	-0.7410102	97	1.94	NCS1	Signaling
DMR9:135888001	9	135888001	135890000	2000	1	1.53E-05	0.188	-0.9299517	52	2.6	CAMSAP1	
DMR9:136873001	9	136873001	136875000	2000	1	8.13E-05	0.288	0.7841544	38	1.9	EDF1;TRAF2	Transcription
DMR10:8681001	10	8681001	8682000	1000	1	9.64E-06	0.162	0.6992715	5	0.5		
DMR10:12708001	10	12708001	12709000	1000	1	7.80E-05	0.283	-0.6661158	21	2.1	CAMK1D	Signaling
DMR10:25848001	10	25848001	25849000	1000	1	6.57E-05	0.276	0.539871	5	0.5		
DMR10:41476001	10	41476001	41478000	2000	1	1.80E-05	0.2	0.7704126	31	1.55		
DMR10:41543001	10	41543001	41544000	1000	1	4.11E-06	0.133	1.0539044	21	2.1		
DMR10:44653001	10	44653001	44654000	1000	1	3.73E-05	0.241	0.8059361	8	0.8		

DMR10:46503001	10	46503001	46505000	2000	1	2.00E-06	0.117	-0.8767672	12	0.6	AC244230.2	
DMR10:70515001	10	70515001	70516000	1000	1	7.72E-06	0.157	-0.6665884	12	1.2	PALD1	Signaling
DMR10:78310001	10	78310001	78312000	2000	1	8.35E-05	0.29	0.5559849	22	1.1	AC012560.1;LINC00595;AC010163.1	
DMR10:78988001	10	78988001	78989000	1000	1	5.01E-06	0.144	-0.7998366	8	0.8	ZMIZ1-AS1	
DMR10:83189001	10	83189001	83190000	1000	1	3.35E-05	0.235	0.9100135	10	1		
DMR10:97678001	10	97678001	97679000	1000	1	2.79E-05	0.223	1.4620037	5	0.5	AL355315.1;PI4K2A;AVPI1	Signaling
DMR10:100603001	10	100603001	100605000	2000	1	4.03E-05	0.247	0.5669876	30	1.5		
DMR10:106291001	10	106291001	106293000	2000	1	9.24E-05	0.297	0.7064251	17	0.85		
DMR10:112684001	10	112684001	112685000	1000	1	1.50E-05	0.188	0.8672151	15	1.5	VTI1A;AL139120.1	Transport
DMR10:132071001	10	132071001	132072000	1000	1	3.07E-05	0.23	0.6428377	5	0.5	JAKMIP3	
DMR10:132134001	10	132134001	132137000	3000	1	2.05E-05	0.21	-0.8705312	132	4.4	JAKMIP3	
DMR11:291001	11	291001	295000	4000	1	2.53E-05	0.213	-0.7234634	135	3.375	NLRP6;AC136475.3;PGGHG;IFITM5;AC136475.6	Unknown
DMR11:2000001	11	2000001	2001000	1000	1	9.64E-05	0.297	-0.8358427	40	4	LINC01219;H19;AC051649.3;MIR675;AC051649.2	
DMR11:22316001	11	22316001	22318000	2000	1	5.64E-05	0.271	0.5856187	42	2.1	AC104009.1	
DMR11:27596001	11	27596001	27597000	1000	1	9.96E-05	0.301	-0.6753202	10	1	BDNF-AS	
DMR11:34139001	11	34139001	34140000	1000	1	1.64E-05	0.188	-0.8460207	15	1.5	NAT10	Metabolism
DMR11:38860001	11	38860001	38861000	1000	1	6.54E-05	0.276	0.6548918	10	1		
DMR11:45068001	11	45068001	45069000	1000	1	9.10E-05	0.297	0.4877675	11	1.1		
DMR11:65868001	11	65868001	65869000	1000	1	2.85E-06	0.123	-1.0209093	26	2.6	CFL1;MUS81;EFEMP2	Cytoskeleton;Transcription;Signaling
DMR11:69681001	11	69681001	69682000	1000	1	6.76E-05	0.276	0.8683811	13	1.3	LTO1	
DMR11:75127001	11	75127001	75128000	1000	1	6.41E-06	0.157	0.9273015	11	1.1	AP001972.4;SLCO2B1;OR2AT1P	Metabolism
DMR11:82132001	11	82132001	82133000	1000	1	2.40E-05	0.212	0.7313282	9	0.9	MIR4300HG	
DMR11:84494001	11	84494001	84496000	2000	1	9.62E-05	0.297	-0.5976226	10	0.5	DLG2	
DMR11:90518001	11	90518001	90519000	1000	1	3.37E-05	0.235	0.7817564	13	1.3	DISC1FP1	
DMR11:105811001	11	105811001	105814000	3000	1	1.60E-05	0.188	0.7735123	29	0.967	GRIA4	Signaling
DMR11:134635001	11	134635001	134636000	1000	1	1.21E-06	0.104	-0.9065303	22	2.2		
DMR12:213001	12	213001	214000	1000	1	3.71E-06	0.129	-0.8199414	28	2.8	SLC6A12;SLC6A12-AS1;SLC6A13	Transport
DMR12:10282001	12	10282001	10283000	1000	1	2.51E-06	0.117	1.0541781	11	1.1	KLRD1	Receptor
DMR12:27484001	12	27484001	27485000	1000	1	3.34E-05	0.235	0.950375	9	0.9	SMCO2	
DMR12:28863001	12	28863001	28864000	1000	1	4.50E-05	0.256	0.6147812	0	0		
DMR12:67329001	12	67329001	67331000	2000	1	9.12E-06	0.158	0.8990534	16	0.8	CAND1;AC078983.1	Transcription
DMR12:71038001	12	71038001	71039000	1000	1	1.01E-05	0.162	0.7541272	0	0	AC123905.1;AC025575.1;AC025575.2	
DMR12:76922001	12	76922001	76923000	1000	1	2.94E-05	0.224	0.6488282	6	0.6		
DMR12:125679001	12	125679001	125680000	1000	1	2.90E-05	0.224	-0.5109605	6	0.6		
DMR12:126898001	12	126898001	126899000	1000	1	6.40E-05	0.276	0.463477	10	1	AC078878.2	
DMR13:27194001	13	27194001	27195000	1000	1	1.64E-05	0.188	-0.7295022	9	0.9	LINC02340	
DMR13:29227001	13	29227001	29228000	1000	1	4.99E-06	0.144	-0.8762244	8	0.8	MTUS2	Cytoskeleton
DMR13:38327001	13	38327001	38328000	1000	1	1.43E-05	0.185	1.0365281	6	0.6	LINC00571	
DMR13:47885001	13	47885001	47886000	1000	1	2.05E-05	0.21	0.8292492	21	2.1	SUCLA2	Metabolism
DMR13:52265001	13	52265001	52268000	3000	1	4.17E-05	0.249	0.7438305	29	0.967	AL158066.1;TPTE2P2	
DMR13:52881001	13	52881001	52882000	1000	1	1.58E-05	0.188	-0.8204531	8	0.8		
DMR13:98681001	13	98681001	98682000	1000	1	2.91E-05	0.224	0.9495598	14	1.4	RN7SL60P;SLC15A1	Metabolism
DMR13:102917001	13	102917001	102918000	1000	1	9.61E-05	0.297	0.5573643	5	0.5		
DMR13:108636001	13	108636001	108637000	1000	1	1.89E-07	0.096	1.1097145	9	0.9	MYO16	Cytoskeleton
DMR13:113649001	13	113649001	113651000	2000	1	7.64E-05	0.283	-0.6693879	55	2.75	TFDP1;ATP4B	Transcription;Transport
DMR14:22610001	14	22610001	22611000	1000	1	6.36E-05	0.276	-0.5716954	20	2	ABHD4	Protease
DMR14:29108001	14	29108001	29110000	2000	1	2.60E-06	0.117	0.7559796	6	0.3	AL135878.1	
DMR14:32238001	14	32238001	32239000	1000	1	9.45E-05	0.297	0.6414987	2	0.2	AL136298.2	
DMR14:34693001	14	34693001	34694000	1000	1	7.55E-05	0.282	-0.7733351	21	2.1	RPL23AP8	
DMR14:61886001	14	61886001	61887000	1000	1	5.93E-05	0.273	0.5587382	20	2	SYT16	Transport
DMR14:68572001	14	68572001	68573000	1000	1	5.90E-05	0.273	-0.6924682	13	1.3	RAD51B	Transcription
DMR14:72554001	14	72554001	72555000	1000	1	5.19E-05	0.268	-0.7107788	14	1.4	RGS6;AC004828.2;AC004828.1	Signaling
DMR14:88545001	14	88545001	88547000	2000	1	2.97E-05	0.225	0.8725831	34	1.7	PTPN21;AL162171.1	Signaling
DMR14:92442001	14	92442001	92444000	2000	1	4.44E-05	0.254	-0.7229027	43	2.15	SLC24A4	Metabolism
DMR14:93377001	14	93377001	93378000	1000	1	1.90E-05	0.205	0.7746667	17	1.7	UNC79	
DMR14:103786001	14	103786001	103787000	1000	1	1.75E-05	0.198	0.9504157	14	1.4	PPP1R13B	Signaling
DMR14:105163001	14	105163001	105165000	2000	1	8.65E-06	0.157	-0.8871449	41	2.05	JAG2;AL512356.1;NUDT14	Growth Factors & Cytokines;Transcription
DMR15:20100001	15	20100001	20108000	8000	1	6.92E-05	0.276	-0.6949394	73	0.912	RN7SL584P	
DMR15:20348001	15	20348001	20351000	3000	1	2.56E-07	0.096	-0.7553247	56	1.867	AC026495.1	
DMR15:20411001	15	20411001	20413000	2000	1	1.11E-05	0.163	-0.7092343	25	1.25	HERC2P3	

DMR15:20772001	15	20772001	20773000	1000	1	3.53E-05	0.24	-0.7242649	17	1.7	AC012414.2;AC012414.3;LONRF2P3	
DMR15:21160001	15	21160001	21165000	5000	1	3.29E-05	0.235	-0.6428106	66	1.32	AC126335.2;BMS1P16	
DMR15:22403001	15	22403001	22405000	2000	2	7.93E-06	0.157	-1.0018406	28	1.4	SPATA31E3P	
DMR15:23165001	15	23165001	23168000	3000	1	7.59E-05	0.282	-0.843789	55	1.833	GOLGA8DP;RN7SL106P	
DMR15:40144001	15	40144001	40145000	1000	1	1.06E-05	0.162	0.8340852	4	0.4	AC021755.1	
DMR15:42607001	15	42607001	42608000	1000	1	1.60E-05	0.188	0.8328186	8	0.8	STARD9	
DMR15:45970001	15	45970001	45971000	1000	1	1.25E-05	0.182	0.6484186	3	0.3		
DMR15:56314001	15	56314001	56315000	1000	1	8.81E-05	0.297	-0.6246418	7	0.7	TEX9;HMGB1P33	Cytoskeleton
DMR15:65746001	15	65746001	65747000	1000	1	6.65E-05	0.276	0.6389006	22	2.2	DENND4A;RAB11A	Signaling
DMR15:66209001	15	66209001	66210000	1000	1	2.43E-05	0.212	0.9280683	19	1.9	MEGF11	Extracellular Matrix
DMR15:69885001	15	69885001	69886000	1000	1	1.49E-05	0.188	-1.0197698	19	1.9		
DMR15:80970001	15	80970001	80971000	1000	1	2.91E-05	0.224	-0.7367371	15	1.5	MESD	
DMR15:82107001	15	82107001	82108000	1000	1	9.65E-05	0.297	0.5663545	10	1	LINC01583;AC026956.2	
DMR15:82922001	15	82922001	82923000	1000	1	1.90E-05	0.205	-0.8630629	6	0.6	HOMER2;AC022558.2	Signaling
DMR15:85789001	15	85789001	85790000	1000	1	2.44E-05	0.212	-0.9050189	19	1.9	KLHL25	Transcription
DMR15:90639001	15	90639001	90641000	2000	1	1.16E-06	0.104	0.8964048	36	1.8	CRTC3;CRTC3-AS1;AC103739.3;HSPE1P3;AC021422.1	Transcription
DMR15:93099001	15	93099001	93100000	1000	1	7.68E-06	0.157	0.7996016	16	1.6	RGMA	
DMR15:93182001	15	93182001	93184000	2000	1	6.91E-05	0.276	-0.5481681	33	1.65	AC108457.1	
DMR15:93978001	15	93978001	93979000	1000	1	1.11E-06	0.104	0.576378	7	0.7	LINC01579;LINC01580;LINC01581	
DMR15:98104001	15	98104001	98107000	3000	1	8.18E-07	0.102	-0.7525215	98	3.267	AC022523.1;LINC01582;AC022523.3	
DMR15:99640001	15	99640001	99641000	1000	1	5.82E-05	0.273	0.5653434	6	0.6	MEF2A	Transcription
DMR15:100680001	15	100680001	100681000	1000	1	3.66E-05	0.241	0.7541443	11	1.1		
DMR16:3849001	16	3849001	3850000	1000	1	1.59E-05	0.188	1.0806486	10	1	CREBBP;AC007151.1	Transcription
DMR16:11402001	16	11402001	11403000	1000	1	7.12E-07	0.102	-0.774745	33	3.3	AC099489.1	
DMR16:12575001	16	12575001	12576000	1000	1	5.51E-05	0.268	0.8463193	15	1.5	SNX29;AC010333.2	Cytoskeleton
DMR16:16283001	16	16283001	16285000	2000	1	7.58E-05	0.282	-0.6194839	36	1.8	NOMO3;AC136624.2	Development
DMR16:16470001	16	16470001	16471000	1000	1	2.06E-05	0.21	0.723135	6	0.6		
DMR16:22892001	16	22892001	22893000	1000	1	5.84E-05	0.273	0.7139617	12	1.2	HS3ST2	Golgi
DMR16:29114001	16	29114001	29115000	1000	1	3.75E-05	0.241	-0.8175252	13	1.3	AC009093.11;AC009093.3;RRN3P2;AC009093.9;AC009093.8	
DMR16:32883001	16	32883001	32887000	4000	1	8.55E-05	0.294	-0.6698009	250	6.25	BCAP31P2;SLC6A10P;AC142086.1	
DMR16:34460001	16	34460001	34462000	2000	2	1.52E-06	0.11	0.6873079	10	0.5		
DMR16:34941001	16	34941001	34943000	2000	1	4.60E-05	0.257	0.4237152	17	0.85	AC135776.4	
DMR16:36487001	16	36487001	36488000	1000	1	7.04E-05	0.278	0.8576506	20	2		
DMR16:36561001	16	36561001	36562000	1000	1	7.55E-05	0.282	0.8607738	17	1.7		
DMR16:37801001	16	37801001	37802000	1000	1	2.47E-05	0.212	1.0127235	19	1.9		
DMR16:49305001	16	49305001	49306000	1000	1	4.32E-05	0.254	0.6442511	7	0.7	AC007614.1;AC007614.2	
DMR16:50324001	16	50324001	50325000	1000	1	3.34E-06	0.129	-0.5905887	10	1	ADCY7;BRD7	Metabolism;Epigenetic
DMR16:56323001	16	56323001	56324000	1000	1	3.74E-05	0.241	-0.6277229	5	0.5	GNAO1	Signaling
DMR16:60843001	16	60843001	60844000	1000	1	7.33E-05	0.282	1.0200138	5	0.5		
DMR16:74425001	16	74425001	74426000	1000	1	2.79E-05	0.223	-0.6509885	14	1.4	CLEC18B;AC009053.3	
DMR16:78331001	16	78331001	78332000	1000	1	2.43E-05	0.212	-0.7229409	13	1.3	WWOX	Metabolism
DMR16:88728001	16	88728001	88730000	2000	1	3.31E-05	0.235	-0.7947117	72	3.6	PIEZO1;AC138028.4;AC138028.2	
DMR17:131001	17	131001	132000	1000	1	4.36E-05	0.254	-0.7334576	19	1.9	SCGB1C2	
DMR17:17799001	17	17799001	17801000	2000	1	6.53E-06	0.157	-0.6519035	55	2.75	RAI1;SREBF1	Transcription
DMR17:18786001	17	18786001	18787000	1000	1	2.83E-05	0.223	-0.8029019	9	0.9	FBXW10;TVP23B	
DMR17:32206001	17	32206001	32207000	1000	1	5.18E-05	0.268	0.681169	19	1.9	RHOT1	Signaling
DMR17:39355001	17	39355001	39356000	1000	1	5.33E-05	0.268	0.6599387	18	1.8	FBXL20	Transcription
DMR17:41235001	17	41235001	41237000	2000	1	8.82E-05	0.297	0.5476374	13	0.65	KRTAP9-2;KRTAP9-3;KRTAP9-8	Cytoskeleton
DMR17:44193001	17	44193001	44194000	1000	1	4.20E-05	0.249	-0.9598986	14	1.4	ASB16-AS1;TMUB2;ATXN7L3;AC004596.1	
DMR17:47475001	17	47475001	47479000	4000	1	5.43E-05	0.268	0.671257	56	1.4	MRPL45P2	
DMR17:77400001	17	77400001	77404000	4000	1	9.81E-05	0.301	-0.5298062	120	3	SEPTIN9;MIR4316	
DMR18:20937001	18	20937001	20939000	2000	1	6.89E-05	0.276	0.8784194	32	1.6	ROCK1	Signaling
DMR18:25869001	18	25869001	25870000	1000	1	8.09E-05	0.288	0.5573316	11	1.1		
DMR18:26302001	18	26302001	26304000	2000	1	2.73E-05	0.222	0.8224402	9	0.45	TAF4B	Transcription
DMR18:26393001	18	26393001	26394000	1000	1	8.57E-06	0.157	0.6613561	11	1.1	TAF4B	Transcription
DMR18:41468001	18	41468001	41469000	1000	1	4.53E-05	0.257	0.7347635	13	1.3	AC079052.1;KC6	
DMR18:51243001	18	51243001	51245000	2000	1	3.19E-06	0.129	0.7132363	22	1.1		
DMR18:58233001	18	58233001	58235000	2000	1	7.45E-06	0.157	0.7750099	14	0.7	NEDD4L	Protease

DMR18:58941001	18	58941001	58942000	1000	1	2.21E-05	0.212	0.5296753	4	0.4	ZNF532	Transcription
DMR18:64318001	18	64318001	64320000	2000	1	1.28E-05	0.182	0.5756697	12	0.6	LINC01924	
DMR18:76781001	18	76781001	76782000	1000	1	7.70E-05	0.283	0.6862806	18	1.8		
DMR19:1170001	19	1170001	1172000	2000	1	4.18E-05	0.249	-0.6990396	95	4.75	SBNO2;STK11	Transcription;Signaling
DMR19:4247001	19	4247001	4248000	1000	1	8.39E-06	0.157	1.357134	35	3.5	EBI3;AC005578.1;YJU2	Receptor
DMR19:6225001	19	6225001	6228000	3000	1	8.67E-05	0.296	-0.7084853	102	3.4	MLLT1	Development
DMR19:8262001	19	8262001	8263000	1000	1	4.44E-05	0.254	-0.9073311	16	1.6	CERS4	
DMR19:11027001	19	11027001	11028000	1000	1	2.46E-06	0.117	-0.8806801	25	2.5	SMARCA4;AC006127.1	Epigenetic
DMR19:14331001	19	14331001	14332000	1000	1	9.03E-05	0.297	0.7054671	15	1.5	LINC01841;LINC01842	
DMR19:14562001	19	14562001	14564000	2000	1	5.54E-05	0.268	-0.7597192	66	3.3	DNAJB1;TECR;NDUFB7	Transcription;Cytoskeleton;Metabolism
DMR19:15686001	19	15686001	15687000	1000	1	3.44E-05	0.238	0.5128054	4	0.4	CYP4F12	Metabolism
DMR19:19342001	19	19342001	19343000	1000	1	6.53E-06	0.157	-0.9095122	33	3.3	MAU2	
DMR19:33167001	19	33167001	33168000	1000	1	7.42E-06	0.157	0.6284587	5	0.5	WDR88;LRP3	Unknown;Receptor
DMR19:35526001	19	35526001	35527000	1000	1	5.79E-05	0.273	-0.7958351	14	1.4	SBSN;GAPDHS	
DMR19:37615001	19	37615001	37616000	1000	1	4.38E-05	0.254	0.6985741	18	1.8	ZNF540;ZFP30	Transcription
DMR19:46870001	19	46870001	46871000	1000	1	8.49E-05	0.294	0.7058523	30	3	ARHGAP35	Signaling
DMR19:47644001	19	47644001	47645000	1000	1	7.53E-05	0.282	0.6801059	11	1.1	AC010519.1;BICRA	
DMR19:48285001	19	48285001	48286000	1000	1	7.77E-05	0.283	0.5747382	12	1.2	ZNF114	Transcription
DMR19:54221001	19	54221001	54223000	2000	2	2.31E-05	0.212	-0.904809	40	2	RPS9;LILRB3;AC245052.7;AC245052.3	Translation;Receptor
DMR19:58498001	19	58498001	58499000	1000	1	3.88E-05	0.245	-0.7498879	25	2.5	SLC27A5;RN7SL693P;AC012313.9	Transport
DMR20:1684001	20	1684001	1685000	1000	1	2.54E-05	0.213	0.7871246	2	0.2	SIRPB3P;AL109809.3	
DMR20:16679001	20	16679001	16680000	1000	1	4.11E-07	0.101	-0.712285	7	0.7	Y_RNA;AL135938.1	
DMR20:26933001	20	26933001	26934000	1000	1	3.80E-06	0.129	-0.6479708	19	1.9		
DMR20:32631001	20	32631001	32633000	2000	1	9.73E-06	0.162	0.7325112	34	1.7	C20orf203;FO393400.1	
DMR20:37163001	20	37163001	37165000	2000	1	9.30E-05	0.297	0.5809423	16	0.8	MROH8;AL031659.1	
DMR20:43626001	20	43626001	43628000	2000	1	9.09E-05	0.297	0.6454519	21	1.05	IFT52	Cytoskeleton
DMR20:48577001	20	48577001	48579000	2000	1	1.61E-06	0.11	-0.8503254	38	1.9		
DMR20:51545001	20	51545001	51546000	1000	1	2.86E-05	0.224	0.3438217	6	0.6	NFATC2	Transcription
DMR21:9768001	21	9768001	9769000	1000	1	6.73E-05	0.276	-0.554014	40	4		
DMR21:23101001	21	23101001	23102000	1000	1	9.38E-05	0.297	0.835228	25	2.5	AP001116.1;ZNF299P;MSANTD2P1;AP001255.1	
DMR21:28877001	21	28877001	28878000	1000	1	6.21E-05	0.276	0.5260008	3	0.3	N6AMT1;HSPD1P7	Transcription
DMR21:33577001	21	33577001	33578000	1000	1	2.56E-06	0.117	1.315097	4	0.4	SON;DONSON;AP000311.1	Transcription
DMR21:43443001	21	43443001	43444000	1000	1	8.38E-06	0.157	-0.8969238	21	2.1	LINC00319	
DMR21:45355001	21	45355001	45358000	3000	1	7.27E-05	0.282	-0.8196857	88	2.933		
DMR21:45367001	21	45367001	45368000	1000	1	2.04E-06	0.117	-0.7561349	26	2.6	MTCO1P3	
DMR22:19770001	22	19770001	19772000	2000	1	5.45E-05	0.268	-0.6160679	42	2.1	TBX1	Epigenetic
DMR22:23154001	22	23154001	23155000	1000	1	1.01E-05	0.162	-0.6849688	14	1.4	RSPH14;RAB36	Signaling
DMR22:40320001	22	40320001	40321000	1000	1	6.63E-05	0.276	0.7500674	8	0.8	TNRC6B	Apoptosis
DMR22:41606001	22	41606001	41608000	2000	1	9.23E-05	0.297	-0.719079	25	1.25	DESI1	Unknown
DMR22:49219001	22	49219001	49221000	2000	1	2.48E-05	0.212	0.8028285	18	0.9	RPL35P8	
DMRX:823001	X	823001	825000	2000	1	9.27E-05	0.297	0.9054493	22	1.1		
DMRX:1070001	X	1070001	1072000	2000	1	7.50E-05	0.282	0.4906729	36	1.8		
DMRX:2276001	X	2276001	2284000	8000	1	1.08E-05	0.162	0.511984	156	1.95	DHRSX	Metabolism
DMRX:13967001	X	13967001	13968000	1000	1	3.64E-05	0.241	-0.7955154	14	1.4	AC003035.2	
DMRX:14685001	X	14685001	14686000	1000	1	8.05E-05	0.288	-0.6345654	8	0.8	GLRA2	Receptor
DMRX:14974001	X	14974001	14975000	1000	1	6.44E-05	0.276	-0.7153719	22	2.2	TPT1P14	
DMRX:27012001	X	27012001	27013000	1000	1	6.10E-05	0.276	0.8518343	12	1.2		
DMRX:28676001	X	28676001	28677000	1000	1	3.05E-05	0.229	0.8784512	12	1.2	IL1RAPL1	Receptor
DMRX:29158001	X	29158001	29159000	1000	1	1.08E-05	0.162	0.6915737	10	1	IL1RAPL1	Receptor
DMRX:46894001	X	46894001	46895000	1000	1	2.42E-05	0.212	-0.6946073	9	0.9	LINC01545	
DMRX:50982001	X	50982001	50983000	1000	1	9.41E-05	0.297	0.795437	16	1.6		
DMRX:57532001	X	57532001	57534000	2000	1	4.68E-06	0.144	0.7234373	4	0.2		
DMRX:58536001	X	58536001	58556000	20000	1	8.26E-05	0.288	0.7321276	384	1.92		
DMRX:62463001	X	62463001	62505000	42000	4	2.22E-05	0.212	0.779105	846	2.014		
DMRX:68317001	X	68317001	68318000	1000	1	6.51E-06	0.157	0.6690277	7	0.7	OPHN1	Development
DMRX:77556001	X	77556001	77557000	1000	1	1.41E-05	0.185	0.9082523	5	0.5	ATRX	Epigenetic
DMRX:87174001	X	87174001	87176000	2000	1	5.33E-06	0.148	0.9394219	8	0.4		
DMRX:155466001	X	155466001	155468000	2000	1	8.63E-05	0.295	0.662469	24	1.2	F8A3;MIR1184-3;H2AB3;TMLHE-AS1;BX571846.1	Immune
