# Supplementary material for: Epigenome association study for DNA methylation biomarkers in buccal and monocyte cells for female rheumatoid arthritis
Source: Sci Rep. 2021 Dec 10;11:23789. doi: 10.1038/s41598-021-03170-6 (PMC8664902; doi:10.1038/s41598-021-03170-6)
Supplement: Supplementary file 4 — Supplementary Table S2. [file 41598_2021_3170_MOESM4_ESM.pdf]

Supplemental Table S2
DMR Table RA CC Monocyte 1e-04

DMR Name	Chr	Start	Stop	Length	# Sig Win	minP	Min FDR	maxLFC	CpG #	CpG Density	Gene Annotation	Gene Category
DMR1:5394001	1	5394001	5395000	1000	1	7.45E-05	0.162	-0.8580598	8	0.8		
DMR1:7498001	1	7498001	7501000	3000	1	3.99E-05	0.14	0.4653717	45	1.5	CAMTA1	Transcription
DMR1:11487001	1	11487001	11488000	1000	1	6.28E-06	0.082	-0.8574727	13	1.3	DISP3	
DMR1:19602001	1	19602001	19604000	2000	1	5.01E-05	0.149	-0.9787791	19	0.95	MICOS10;AL031727.2;NBL1;MICOS10-NBL1;AL031727.1	Transcription
DMR1:26920001	1	26920001	26921000	1000	1	1.50E-05	0.1	0.5372672	14	1.4	NUDC;NR0B2	Cytoskeleton;Receptor
DMR1:37712001	1	37712001	37713000	1000	1	4.77E-06	0.077	-0.824134	9	0.9	CDCA8;EPHA10	Receptor
DMR1:40351001	1	40351001	40352000	1000	1	9.66E-05	0.178	0.5274836	17	1.7	SMAP2	Transcription
DMR1:41424001	1	41424001	41427000	3000	1	5.29E-05	0.15	0.4401215	46	1.533		
DMR1:48776001	1	48776001	48777000	1000	1	8.61E-05	0.171	-1.1981384	68	6.8	AGBL4;BEND5	Signaling
DMR1:51156001	1	51156001	51158000	2000	1	3.45E-05	0.135	-0.8468118	11	0.55	C1orf185;CFL1P2	
DMR1:53945001	1	53945001	53946000	1000	1	9.87E-05	0.178	-1.0298376	90	9	HSPB11;LRRC42	Signaling;Unknown
DMR1:58145001	1	58145001	58146000	1000	1	7.79E-05	0.167	-0.7784909	13	1.3	DAB1	Signaling
DMR1:59916001	1	59916001	59918000	2000	1	7.60E-05	0.164	0.6860717	22	1.1	CYP2J2	Electron Transport
DMR1:60029001	1	60029001	60030000	1000	1	6.93E-05	0.16	0.4251821	11	1.1	C1orf87	
DMR1:77905001	1	77905001	77907000	2000	1	1.96E-05	0.109	-0.9831377	14	0.7	NEXN	
DMR1:84706001	1	84706001	84708000	2000	1	2.55E-05	0.118	-0.88394	3	0.15		
DMR1:89884001	1	89884001	89885000	1000	1	5.74E-05	0.154	-0.6997991	13	1.3	AC093423.3;LRRC8D	Unknown
DMR1:91951001	1	91951001	91952000	1000	1	9.50E-05	0.178	0.4606705	22	2.2	BRDT	Epigenetic
DMR1:92699001	1	92699001	92700000	1000	1	5.97E-05	0.156	-1.1432329	7	0.7	EVI5;RNU4-59P	
DMR1:101332001	1	101332001	101333000	1000	1	6.29E-05	0.156	-0.9838937	3	0.3	LINC01307	
DMR1:109828001	1	109828001	109829000	1000	1	2.00E-05	0.109	-0.9561344	16	1.6	LINC01768	
DMR1:110291001	1	110291001	110293000	2000	2	3.10E-05	0.127	-0.9810789	17	0.85	KCNC4;RBM15-AS1	Transport
DMR1:114726001	1	114726001	114728000	2000	1	1.56E-05	0.102	-0.9359876	15	0.75	NRAS;CSDE1;RNY1P13	Transcription
DMR1:120751001	1	120751001	120752000	1000	1	1.63E-05	0.103	-0.8365398	7	0.7	NOTCH2NLR;NBPF26	
DMR1:120941001	1	120941001	120942000	1000	1	8.85E-05	0.174	0.6612918	15	1.5	LINC00623;RNVU1-4	
DMR1:144782001	1	144782001	144783000	1000	1	7.99E-05	0.17	-0.9595865	12	1.2	FP700111.1;FP700111.2	
DMR1:150729001	1	150729001	150730000	1000	1	1.40E-05	0.098	0.8127482	63	6.3	HORMAD1;CTSS	Development;Proteolysis
DMR1:152171001	1	152171001	152172000	1000	1	6.19E-07	0.04	-0.8225655	4	0.4	PUDPP2;FLG-AS1	
DMR1:152471001	1	152471001	152472000	1000	1	5.12E-05	0.149	0.8319539	10	1		
DMR1:152877001	1	152877001	152879000	2000	1	2.30E-05	0.113	-1.20735	8	0.4	SMCP	
DMR1:157662001	1	157662001	157663000	1000	1	1.67E-05	0.104	-1.0930505	8	0.8		
DMR1:161569001	1	161569001	161571000	2000	1	1.89E-05	0.109	-0.9246235	17	0.85	AL590385.2	
DMR1:165267001	1	165267001	165268000	1000	1	3.23E-05	0.13	-0.9248399	3	0.3	LMX1A	Transcription
DMR1:167894001	1	167894001	167897000	3000	1	3.60E-05	0.136	-0.8752288	23	0.767	ADCY10	Signaling
DMR1:170050001	1	170050001	170051000	1000	1	4.32E-05	0.143	-0.7407794	7	0.7	KIFAP3	Cytoskeleton
DMR1:176295001	1	176295001	176296000	1000	1	4.52E-05	0.145	-0.7262141	6	0.6	AL591043.2;AL591043.1	
DMR1:177190001	1	177190001	177191000	1000	1	2.47E-05	0.117	0.6115749	7	0.7	BRINP2	
DMR1:203785001	1	203785001	203786000	1000	1	8.43E-05	0.171	-0.9694419	5	0.5	LAX1;ZC3H11A;ZBED6	Transcription
DMR1:205912001	1	205912001	205913000	1000	1	1.37E-05	0.098	-0.6629078	10	1	SLC26A9	Transport
DMR1:205956001	1	205956001	205957000	1000	1	1.18E-05	0.098	-0.7986133	11	1.1		
DMR1:205972001	1	205972001	205976000	4000	1	5.46E-05	0.152	0.4220145	50	1.25	RAB7B	Signaling
DMR1:209770001	1	209770001	209772000	2000	1	2.08E-05	0.11	0.6539779	47	2.35	TRAF3IP3;C1orf74	Unknown
DMR1:213945001	1	213945001	213946000	1000	1	4.16E-05	0.141	-0.7652893	5	0.5	PROX1-AS1	
DMR1:215255001	1	215255001	215256000	1000	1	2.68E-05	0.12	-0.9513034	8	0.8		
DMR1:218693001	1	218693001	218694000	1000	1	1.41E-05	0.098	-1.0480148	10	1		
DMR1:220127001	1	220127001	220128000	1000	1	1.31E-05	0.098	-0.7808259	6	0.6	IARS2;MIR215;MIR194-1	Metabolism
DMR1:221233001	1	221233001	221235000	2000	1	6.02E-05	0.156	-0.7138824	19	0.95		
DMR1:223199001	1	223199001	223200000	1000	1	3.18E-05	0.13	-1.0026597	7	0.7		
DMR1:225519001	1	225519001	225520000	1000	1	6.22E-05	0.156	0.557504	19	1.9	ENAH	Signaling
DMR1:226785001	1	226785001	226786000	1000	1	5.68E-05	0.154	0.6241232	25	2.5	RPS27P5	
DMR1:236100001	1	236100001	236103000	3000	1	1.08E-05	0.097	0.5359867	63	2.1	AL122018.1	
DMR1:237846001	1	237846001	237848000	2000	1	8.82E-06	0.088	-0.7434129	19	0.95		
DMR1:237941001	1	237941001	237942000	1000	1	8.27E-05	0.171	0.6866161	11	1.1	MTCO1P38;AL590396.2;AL590396.3;MTRNR2L11;MTCYBP15;MTND6P15;MTND5P18	
DMR1:239270001	1	239270001	239271000	1000	1	2.31E-09	0.003	-1.1083838	10	1		
DMR1:248752001	1	248752001	248755000	3000	1	2.74E-05	0.12	0.4353906	24	0.8	CR589904.2;LYPD8	
DMR2:317001	2	317001	320000	3000	1	3.85E-05	0.14	0.5383305	104	3.467	AC079779.3;AC079779.4;LINC01865	
DMR2:329001	2	329001	330000	1000	1	9.38E-05	0.177	-0.9319475	6	0.6	LINC01865	
DMR2:1679001	2	1679001	1681000	2000	2	1.01E-05	0.091	0.5911597	55	2.75	PXDN	Metabolism

DMR2:3526001	2	3526001	3529000	3000	1	3.34E-07	0.038	0.5898772	92	3.067	ADI1;AC231981.1;AC108488.1;AC108488.2	Metabolism
DMR2:22949001	2	22949001	22950000	1000	1	6.58E-05	0.158	-0.9822151	3	0.3		
DMR2:26424001	2	26424001	26425000	1000	1	9.01E-05	0.174	0.5206402	16	1.6	DRC1	
DMR2:30116001	2	30116001	30117000	1000	1	2.58E-06	0.067	-1.1087956	11	1.1	AC016907.2	
DMR2:31136001	2	31136001	31137000	1000	1	7.65E-05	0.165	-1.1235895	12	1.2	GALNT14	Metabolism
DMR2:33351001	2	33351001	33352000	1000	1	4.93E-05	0.149	-0.972549	6	0.6	LTBP1	Receptor
DMR2:45657001	2	45657001	45658000	1000	1	3.34E-05	0.132	-1.0569781	13	1.3	PRKCE	Binding Protein
DMR2:55138001	2	55138001	55139000	1000	1	4.90E-05	0.149	-0.6872517	12	1.2		
DMR2:56255001	2	56255001	56256000	1000	1	5.33E-05	0.15	-0.706649	3	0.3	AC007744.1;CCDC85A	
DMR2:63614001	2	63614001	63615000	1000	1	5.60E-05	0.153	-0.7415433	4	0.4	WDPCP;MDH1;PRELID1P6	Metabolism
DMR2:65634001	2	65634001	65636000	2000	1	7.28E-05	0.161	0.5488589	17	0.85	AC007389.1;AC007389.2;AC007389.3	
DMR2:72735001	2	72735001	72736000	1000	1	1.14E-05	0.098	0.5877386	10	1	EXOC6B	Transport
DMR2:84349001	2	84349001	84350000	1000	1	5.59E-05	0.153	-0.9905806	4	0.4	AC106874.1	
DMR2:86154001	2	86154001	86155000	1000	1	1.36E-05	0.098	0.6034925	21	2.1	IMMT;Y_RNA	
DMR2:86972001	2	86972001	86973000	1000	1	6.06E-05	0.156	0.5028754	25	2.5	RGPD1	Signaling
DMR2:91430001	2	91430001	91431000	1000	1	6.38E-05	0.157	-1.1806431	7	0.7		
DMR2:99285001	2	99285001	99286000	1000	1	5.82E-05	0.155	-0.67255	12	1.2	AC079447.1;LYG1	
DMR2:101639001	2	101639001	101640000	1000	1	4.68E-05	0.147	-0.7118198	24	2.4		
DMR2:120187001	2	120187001	120188000	1000	1	5.74E-05	0.154	-1.1539445	5	0.5	EPB41L5;AC012363.1;Y_RNA	
DMR2:121543001	2	121543001	121544000	1000	1	2.97E-05	0.122	-1.1305347	7	0.7	CLASP1	Unknown
DMR2:122003001	2	122003001	122004000	1000	1	6.76E-05	0.159	-0.9085215	13	1.3	AC011246.1	
DMR2:122734001	2	122734001	122735000	1000	1	7.01E-06	0.082	-0.8796255	7	0.7	AC011246.1	
DMR2:124318001	2	124318001	124319000	1000	1	8.93E-06	0.088	-1.2396233	5	0.5	CNTNAP5	Receptor
DMR2:127043001	2	127043001	127046000	3000	1	1.21E-05	0.098	0.5472903	78	2.6	AC012508.3;BIN1	Receptor
DMR2:132272001	2	132272001	132274000	2000	1	3.36E-06	0.07	0.6323749	78	3.9	CDC27P1	
DMR2:133299001	2	133299001	133302000	3000	1	4.09E-05	0.141	-0.7173837	33	1.1	NCKAP5	
DMR2:141057001	2	141057001	141058000	1000	1	3.38E-05	0.134	-0.9278945	6	0.6	LRP1B	Metabolism
DMR2:144735001	2	144735001	144736000	1000	1	7.18E-05	0.16	-0.9530131	2	0.2	TEX41	
DMR2:148263001	2	148263001	148264000	1000	1	8.56E-05	0.171	-0.8228011	4	0.4	MBD5;AC019070.1	
DMR2:151714001	2	151714001	151715000	1000	1	9.11E-06	0.089	-0.8501989	4	0.4	NEB	Cytoskeleton
DMR2:161288001	2	161288001	161290000	2000	1	5.92E-05	0.155	-0.6176243	14	0.7	AC009299.2;AC009299.1	
DMR2:168116001	2	168116001	168117000	1000	1	8.48E-06	0.087	-1.2265459	8	0.8	STK39	
DMR2:169757001	2	169757001	169758000	1000	1	7.94E-07	0.045	-1.3976683	6	0.6	KLHL23;PTCHD3P2	
DMR2:170465001	2	170465001	170467000	2000	1	1.33E-05	0.098	-0.8244985	29	1.45	MYO3B	Cytoskeleton
DMR2:171162001	2	171162001	171163000	1000	1	8.89E-05	0.174	0.5831447	23	2.3	TLK1	Signaling
DMR2:177114001	2	177114001	177115000	1000	1	1.94E-05	0.109	0.704493	8	0.8	AC074286.1;AC079305.2	
DMR2:182786001	2	182786001	182787000	1000	1	4.51E-06	0.075	-0.9308816	9	0.9	DNAJC10;RPL31P15	Transcription
DMR2:185762001	2	185762001	185763000	1000	1	3.92E-05	0.14	-1.1755814	3	0.3	FSIP2-AS1;FSIP2	
DMR2:200968001	2	200968001	200969000	1000	1	9.10E-05	0.174	-0.5764868	5	0.5	ORC2;AC005037.1;FAM126B	Unknown
DMR2:223485001	2	223485001	223486000	1000	1	6.66E-07	0.04	-1.0178309	6	0.6		
DMR2:226896001	2	226896001	226897000	1000	1	4.97E-06	0.077	-0.9589609	7	0.7	RHBDD1	Protease
DMR2:230611001	2	230611001	230612000	1000	1	4.44E-05	0.144	-1.0682547	15	1.5		
DMR2:232651001	2	232651001	232652000	1000	1	2.29E-05	0.113	-0.7125157	10	1	EFHD1;RN7SL359P	Unknown
DMR2:234969001	2	234969001	234972000	3000	1	2.91E-05	0.122	0.5231338	62	2.067	SH3BP4	Signaling
DMR2:240734001	2	240734001	240735000	1000	1	2.85E-05	0.122	0.5034771	32	3.2	KIF1A	Cytoskeleton
DMR2:240961001	2	240961001	240963000	2000	1	1.16E-05	0.098	0.6673889	68	3.4	CROCC2;UICLM	
DMR3:5153001	3	5153001	5154000	1000	1	6.28E-06	0.082	-1.2237828	11	1.1	ARL8B;AC026202.2	Translation
DMR3:5331001	3	5331001	5333000	2000	1	5.74E-05	0.154	-0.8116048	37	1.85		
DMR3:6073001	3	6073001	6074000	1000	1	2.91E-05	0.122	-0.7801978	11	1.1	AC087857.1	
DMR3:13199001	3	13199001	13201000	2000	1	8.87E-05	0.174	0.5345693	51	2.55	IQSEC1	Signaling
DMR3:26843001	3	26843001	26844000	1000	1	5.68E-05	0.154	0.6977539	25	2.5		
DMR3:29052001	3	29052001	29054000	2000	1	2.54E-05	0.118	-0.7548583	10	0.5	AC098650.1;RBMS3;RBMS3-AS3	Epigenetic
DMR3:47453001	3	47453001	47454000	1000	1	4.98E-05	0.149	-0.9217957	4	0.4	SCAP	Translation
DMR3:51960001	3	51960001	51961000	1000	1	8.53E-06	0.087	-0.6736391	21	2.1	AC115284.2;GPR62;PCBP4;AC115284.1;ABHD14B	Receptor;Apoptosis;Metabolism
DMR3:53696001	3	53696001	53697000	1000	1	1.74E-07	0.032	-1.1244011	9	0.9	CACNA1D	Metabolism
DMR3:57558001	3	57558001	57561000	3000	1	1.49E-05	0.1	0.644978	64	2.133	RNU6-483P;AC092418.2;PDE12	
DMR3:68618001	3	68618001	68619000	1000	1	1.58E-06	0.056	-0.8811949	15	1.5		
DMR3:73209001	3	73209001	73210000	1000	1	7.86E-06	0.087	-0.7845738	7	0.7		
DMR3:74531001	3	74531001	74533000	2000	1	1.50E-05	0.1	-1.1000714	15	0.75	CNTN3	Cytoskeleton
DMR3:79130001	3	79130001	79131000	1000	1	9.38E-05	0.177	-0.8905453	8	0.8	ROBO1	Development
DMR3:84908001	3	84908001	84910000	2000	1	6.39E-07	0.04	-0.932254	9	0.45		
DMR3:94074001	3	94074001	94075000	1000	1	6.84E-05	0.16	-1.1906393	5	0.5	NSUN3;RBBP4P2	Transcription

DMR3:106673001	3	106673001	106674000	1000	1	3.90E-05	0.14	-0.9335338	2	0.2	LINC00882	
DMR3:106929001	3	106929001	106930000	1000	1	6.29E-05	0.156	-0.7888353	5	0.5	LINC00882	
DMR3:107665001	3	107665001	107666000	1000	1	2.13E-05	0.111	-1.0842399	10	1	BBX	Transcription
DMR3:115422001	3	115422001	115423000	1000	1	4.17E-05	0.141	-1.159606	5	0.5	AC026341.1;AC026341.2;AC026341.3	
DMR3:120387001	3	120387001	120388000	1000	1	8.16E-05	0.171	0.8675429	12	1.2	AC063952.1;AC063952.3;FSTL1;MIR198	Signaling
DMR3:121470001	3	121470001	121471000	1000	1	5.37E-06	0.078	0.8093882	13	1.3	POLQ	Transcription
DMR3:122930001	3	122930001	122932000	2000	1	7.15E-05	0.16	0.5032562	26	1.3	SEMA5B	
DMR3:124003001	3	124003001	124004000	1000	1	5.84E-05	0.155	-0.8097971	4	0.4		
DMR3:129369001	3	129369001	129371000	2000	1	5.09E-05	0.149	0.3835812	24	1.2		
DMR3:135516001	3	135516001	135517000	1000	1	8.36E-05	0.171	-0.7666261	13	1.3		
DMR3:137718001	3	137718001	137719000	1000	1	3.88E-05	0.14	-1.0724397	2	0.2	NPM1P17	
DMR3:141712001	3	141712001	141713000	1000	1	8.61E-05	0.171	-0.8776809	9	0.9	LINC02618;TPT1P3	
DMR3:151556001	3	151556001	151557000	1000	1	6.48E-05	0.158	-0.9616976	6	0.6	MIR5186	
DMR3:151624001	3	151624001	151625000	1000	1	9.87E-05	0.178	0.8314423	22	2.2		
DMR3:171426001	3	171426001	171428000	2000	1	3.06E-06	0.069	-1.1825452	22	1.1	TNIK	Signaling
DMR3:172115001	3	172115001	172116000	1000	1	4.43E-05	0.144	-1.116794	5	0.5	FNDC3B	Cytoskeleton
DMR3:176427001	3	176427001	176428000	1000	1	6.69E-05	0.159	-0.801649	9	0.9	LINC01208	
DMR3:178056001	3	178056001	178057000	1000	1	5.91E-05	0.155	0.8266747	15	1.5	AC110992.1	
DMR3:181678001	3	181678001	181679000	1000	1	4.68E-07	0.04	-1.2547941	8	0.8	SOX2-OT;AC125613.1	
DMR3:185898001	3	185898001	185899000	1000	1	4.26E-05	0.142	-0.9143219	12	1.2		
DMR3:187694001	3	187694001	187695000	1000	1	1.87E-05	0.109	-0.8787577	4	0.4	RTP2;AC072022.1	
DMR3:192567001	3	192567001	192568000	1000	1	8.30E-06	0.087	-0.9433436	8	0.8	FGF12	Growth Factors & Cytokines
DMR3:193511001	3	193511001	193512000	1000	1	2.87E-05	0.122	-1.0389333	11	1.1	ATP13A4	Transport
DMR3:194072001	3	194072001	194073000	1000	1	4.95E-05	0.149	-0.9522004	16	1.6	LINC02028;AC024559.2	
DMR4:335001	4	335001	336000	1000	1	5.72E-05	0.154	-0.7835109	10	1	AC079140.6;AC079140.4;ZNF141;AC079140.1;AC079140.2	Transcription
DMR4:1434001	4	1434001	1436000	2000	1	2.82E-05	0.122	0.6353971	37	1.85		
DMR4:5604001	4	5604001	5605000	1000	1	9.77E-05	0.178	-0.6050535	9	0.9	EVC2	Development
DMR4:6160001	4	6160001	6162000	2000	1	8.24E-05	0.171	0.4299853	29	1.45	C4orf50;JAKMIP1	
DMR4:6299001	4	6299001	6301000	2000	1	1.66E-05	0.103	0.5408859	59	2.95	WFS1;AC116317.1	Development
DMR4:6533001	4	6533001	6535000	2000	1	3.48E-06	0.07	0.5564613	37	1.85	PPP2R2C	Signaling
DMR4:6700001	4	6700001	6702000	2000	1	4.30E-06	0.074	0.6495516	47	2.35	LINC02481;AC093323.2;S100P;AC093323.3;MRFAP1L1	Signaling
DMR4:7639001	4	7639001	7641000	2000	1	7.14E-05	0.16	0.5223761	24	1.2	SORCS2	Receptor
DMR4:14311001	4	14311001	14312000	1000	1	9.55E-05	0.178	-0.6829741	3	0.3		
DMR4:42666001	4	42666001	42667000	1000	1	7.10E-05	0.16	-0.9941612	6	0.6	ATP8A1;AC096734.2	Metabolism
DMR4:48798001	4	48798001	48799000	1000	1	9.00E-05	0.174	-0.673604	5	0.5	OCIAD1	Cytoskeleton
DMR4:93984001	4	93984001	93986000	2000	1	4.00E-05	0.14	-0.7984261	10	0.5		
DMR4:100293001	4	100293001	100294000	1000	1	3.05E-06	0.069	-1.2701406	5	0.5		
DMR4:104848001	4	104848001	104849000	1000	1	2.66E-05	0.12	0.6396126	15	1.5	AC004053.1	
DMR4:109141001	4	109141001	109142000	1000	1	8.77E-05	0.173	-0.6178277	6	0.6	COL25A1	Extracellular Matrix
DMR4:121519001	4	121519001	121520000	1000	1	3.93E-05	0.14	-0.6628217	8	0.8		
DMR4:123709001	4	123709001	123710000	1000	1	5.33E-05	0.15	-0.7770373	7	0.7	LINC01091	
DMR4:124648001	4	124648001	124649000	1000	1	8.47E-06	0.087	-0.9396036	5	0.5		
DMR4:126334001	4	126334001	126335000	1000	1	9.92E-06	0.091	-1.1769753	3	0.3		
DMR4:129774001	4	129774001	129775000	1000	1	3.33E-05	0.132	-1.0945687	5	0.5	LINC02466;LINC02465	
DMR4:136787001	4	136787001	136788000	1000	1	4.00E-05	0.14	-1.0539732	5	0.5	LINC02511	
DMR4:145377001	4	145377001	145379000	2000	1	3.22E-05	0.13	-0.9253434	25	1.25	RTN3P1;AC079228.1	
DMR4:149835001	4	149835001	149836000	1000	1	2.02E-05	0.109	0.6043071	30	3	IQCM	
DMR4:151646001	4	151646001	151647000	1000	1	2.60E-05	0.118	-0.901252	12	1.2	FAM160A1	
DMR4:158044001	4	158044001	158046000	2000	1	7.08E-05	0.16	0.7907477	61	3.05	AC084740.1	
DMR4:161359001	4	161359001	161360000	1000	1	1.38E-05	0.098	-1.2074632	6	0.6		
DMR4:170498001	4	170498001	170499000	1000	1	4.95E-05	0.149	-0.5603718	8	0.8		
DMR4:177392001	4	177392001	177393000	1000	1	9.33E-05	0.176	-0.9903147	7	0.7		
DMR4:181652001	4	181652001	181653000	1000	1	6.50E-08	0.024	-1.1374562	8	0.8		
DMR4:184739001	4	184739001	184741000	2000	2	3.09E-07	0.038	-1.2605592	26	1.3	CENPU	
DMR4:185483001	4	185483001	185484000	1000	1	9.69E-05	0.178	0.6072863	8	0.8	AC106897.1	
DMR5:1041001	5	1041001	1050000	9000	1	5.57E-05	0.153	0.449935	174	1.933	NKD2;SLC12A7	Transport
DMR5:4614001	5	4614001	4615000	1000	1	3.84E-06	0.072	-0.951374	10	1	AC106799.2	
DMR5:12673001	5	12673001	12674000	1000	1	6.51E-06	0.082	-0.7666366	11	1.1	LINC01194	
DMR5:12942001	5	12942001	12943000	1000	1	5.51E-05	0.153	0.8943199	7	0.7	LINC02220	
DMR5:41380001	5	41380001	41381000	1000	1	6.70E-05	0.159	-0.8531705	8	0.8	PLCXD3	
DMR5:60559001	5	60559001	60560000	1000	1	6.20E-05	0.156	-0.8742124	8	0.8		
DMR5:64662001	5	64662001	64663000	1000	1	4.18E-05	0.141	-0.9161728	6	0.6		

DMR5:66040001	5	66040001	66042000	2000	1	1.35E-05	0.098	-0.8827077	7	0.35	ERBIN	
DMR5:71608001	5	71608001	71609000	1000	1	2.97E-05	0.122	-0.6098951	8	0.8	MCCC2	Metabolism
DMR5:79340001	5	79340001	79341000	1000	1	2.18E-05	0.113	-1.0503046	10	1		
DMR5:91569001	5	91569001	91570000	1000	1	6.26E-07	0.04	-1.0670839	6	0.6	ARRDC3-AS1	
DMR5:103558001	5	103558001	103559000	1000	1	2.26E-05	0.113	-0.9329858	2	0.2	NUDT12	Metabolism
DMR5:117030001	5	117030001	117031000	1000	1	6.72E-05	0.159	-1.0516846	4	0.4	AC093534.2	
DMR5:119330001	5	119330001	119331000	1000	1	6.90E-05	0.16	-0.657047	12	1.2	TNFAIP8	Unknown
DMR5:127995001	5	127995001	127996000	1000	1	5.97E-05	0.156	-1.0069356	8	0.8	LINC01184	
DMR5:134756001	5	134756001	134757000	1000	1	2.56E-05	0.118	-0.6497842	13	1.3	CAMLG;DDX46	Transcription
DMR5:141444001	5	141444001	141445000	1000	1	9.58E-05	0.178	-0.9044661	8	0.8	PCDHGA1;PCDHGA2;PCDHGA3;PCDHGB1;PCDHGA4;PCDHGB2;PCDHGA5;PCDHGB3;PCDHGA6;PCDHGA7;PCDHGB4;PCDHGA8;PCDHGB5;PCDHGA9;PCDHGB6;PCDHGA10;PCDHGB7;PCDHGA11;PCDHGA12;PCDHGB9P	Extracellular Matrix;EST
DMR5:148156001	5	148156001	148157000	1000	1	1.96E-06	0.061	-0.8422247	3	0.3	AC011346.1	
DMR5:149654001	5	149654001	149656000	2000	1	4.56E-05	0.145	0.425073	17	0.85		
DMR5:152452001	5	152452001	152453000	1000	1	7.17E-05	0.16	-1.2136441	6	0.6	AC008571.2	
DMR5:153469001	5	153469001	153471000	2000	1	4.21E-06	0.074	-0.8978886	8	0.4		
DMR5:154178001	5	154178001	154179000	1000	1	4.01E-05	0.14	-0.9708803	8	0.8	MFAP3	Extracellular Matrix
DMR5:163583001	5	163583001	163585000	2000	1	3.43E-05	0.135	0.6241947	78	3.9		
DMR5:164466001	5	164466001	164468000	2000	1	3.83E-06	0.072	0.6114779	25	1.25	AC109466.1;LINC02143	
DMR5:169226001	5	169226001	169227000	1000	1	9.00E-05	0.174	-0.8413714	13	1.3	SLIT3	Development
DMR5:169841001	5	169841001	169842000	1000	1	7.12E-05	0.16	-0.7835331	10	1	DOCK2	Signaling
DMR5:173340001	5	173340001	173341000	1000	1	5.27E-05	0.15	-0.936532	18	1.8	MIR8056	
DMR5:176274001	5	176274001	176276000	2000	1	4.25E-06	0.074	-0.8612601	13	0.65	SIMC1	
DMR5:176374001	5	176374001	176376000	2000	1	1.72E-05	0.105	0.7386365	22	1.1	ARL10;MIR1271;NOP16	Signaling
DMR5:176470001	5	176470001	176471000	1000	1	1.60E-05	0.103	0.4775324	22	2.2	FAF2	Proteolysis
DMR6:456001	6	456001	457000	1000	1	5.11E-05	0.149	-0.7500874	9	0.9	AL512308.1	
DMR6:1196001	6	1196001	1197000	1000	1	4.46E-05	0.144	-0.9847361	6	0.6		
DMR6:7300001	6	7300001	7302000	2000	1	6.38E-05	0.157	-0.7248362	20	1	SSR1;AL139095.4	Transcription
DMR6:11529001	6	11529001	11530000	1000	1	8.99E-05	0.174	0.7557983	13	1.3	TMEM170B	
DMR6:14006001	6	14006001	14008000	2000	1	1.11E-07	0.031	0.6225383	33	1.65	AL022396.1	
DMR6:18549001	6	18549001	18551000	2000	1	4.41E-06	0.074	0.6472295	14	0.7	MIR548A1HG	
DMR6:22383001	6	22383001	22384000	1000	1	1.33E-05	0.098	-1.0171607	11	1.1	CASC15	
DMR6:35239001	6	35239001	35240000	1000	1	7.67E-06	0.087	-1.2894862	7	0.7	SCUBE3	Extracellular Matrix
DMR6:35342001	6	35342001	35343000	1000	1	1.78E-05	0.107	-0.9916951	79	7.9	PPARD	Receptor
DMR6:40338001	6	40338001	40339000	1000	1	1.71E-05	0.105	0.5160691	11	1.1	TDRG1;LINC00951	
DMR6:41576001	6	41576001	41577000	1000	1	9.87E-05	0.178	0.5263715	17	1.7	FOXP4	Transcription
DMR6:44131001	6	44131001	44133000	2000	1	8.98E-05	0.174	-0.8836256	22	1.1	MRPL14;TMEM63B	Transcription;EST
DMR6:46054001	6	46054001	46055000	1000	1	8.32E-05	0.171	-0.880946	7	0.7	CLIC5	Transport
DMR6:50415001	6	50415001	50416000	1000	1	8.48E-06	0.087	-0.8820504	5	0.5	AL132799.1	
DMR6:55366001	6	55366001	55367000	1000	1	7.67E-05	0.165	-0.6627618	6	0.6	GFRAL	
DMR6:78388001	6	78388001	78389000	1000	1	9.82E-05	0.178	-0.8419495	5	0.5		
DMR6:83252001	6	83252001	83253000	1000	1	2.60E-07	0.037	-0.8739151	5	0.5	ME1	Metabolism
DMR6:83676001	6	83676001	83677000	1000	1	7.10E-06	0.082	-1.0139233	4	0.4	SNAP91	Transport
DMR6:113178001	6	113178001	113179000	1000	1	5.99E-05	0.156	-0.9899988	1	0.1		
DMR6:113374001	6	113374001	113375000	1000	1	2.60E-05	0.118	-1.0667402	8	0.8	AL589684.1	
DMR6:114444001	6	114444001	114445000	1000	1	5.89E-05	0.155	-0.8309278	8	0.8	HDAC2-AS2	
DMR6:116753001	6	116753001	116754000	1000	1	3.21E-05	0.13	-0.8225297	9	0.9	FAM162B	
DMR6:118050001	6	118050001	118051000	1000	1	2.65E-06	0.067	-1.2291346	1	0.1	SLC35F1	Metabolism
DMR6:122848001	6	122848001	122849000	1000	1	3.00E-06	0.069	0.7203493	66	6.6		
DMR6:128306001	6	128306001	128307000	1000	1	7.35E-05	0.161	-0.9087185	7	0.7	PTPRK	Signaling
DMR6:130339001	6	130339001	130340000	1000	1	1.47E-05	0.1	-1.1284268	7	0.7	SAMD3	Signaling
DMR6:133280001	6	133280001	133281000	1000	1	1.28E-06	0.054	0.7322818	14	1.4	EYA4	Transcription
DMR6:138421001	6	138421001	138422000	1000	1	6.11E-05	0.156	-0.830099	18	1.8	HEBP2;NHSL1	Unknown
DMR6:142876001	6	142876001	142877000	1000	1	5.00E-05	0.149	-1.1418874	8	0.8	HIVEP2	Transcription
DMR6:153592001	6	153592001	153593000	1000	1	4.81E-05	0.149	-1.1233359	2	0.2		
DMR6:161374001	6	161374001	161375000	1000	1	4.90E-05	0.149	0.425415	15	1.5	PRKN	
DMR6:163356001	6	163356001	163358000	2000	1	9.24E-05	0.175	0.451748	43	2.15	AL031121.2;AL031121.3;AL031121.1	
DMR6:163945001	6	163945001	163947000	2000	1	1.88E-06	0.061	-0.9312165	34	1.7	AL078602.1;AL136225.1	
DMR6:165708001	6	165708001	165710000	2000	1	7.10E-05	0.16	0.5282343	70	3.5	PDE10A;RNU6-730P	Signaling
DMR6:170123001	6	170123001	170124000	1000	1	3.85E-05	0.14	0.6266025	16	1.6		
DMR7:4954001	7	4954001	4956000	2000	1	5.55E-05	0.153	-0.7196781	21	1.05	MMD2	Development

DMR7:6054001	7	6054001	6055000	1000	1	5.57E-06	0.078	0.7546726	18	1.8	EIF2AK1;RN7SL851P;RNU6-218P	Signaling
DMR7:9385001	7	9385001	9386000	1000	1	1.84E-05	0.108	-0.9213785	8	0.8		
DMR7:12914001	7	12914001	12915000	1000	1	2.43E-05	0.116	-0.8085232	4	0.4	AC011287.1;RBMX2P4	
DMR7:24729001	7	24729001	24730000	1000	1	1.86E-06	0.061	-0.9496471	17	1.7	GSDME	
DMR7:32298001	7	32298001	32299000	1000	1	4.01E-05	0.14	-0.7631104	78	7.8	PDE1C	Metabolism
DMR7:34635001	7	34635001	34637000	2000	1	3.82E-05	0.14	-1.0018021	5	0.25	NPSR1-AS1	
DMR7:38371001	7	38371001	38372000	1000	1	3.46E-05	0.135	0.4663782	17	1.7	TRG-AS1;TRGV2;TRGV1	
DMR7:38562001	7	38562001	38563000	1000	1	6.79E-05	0.159	-0.8236264	7	0.7	AMPH	Receptor
DMR7:45881001	7	45881001	45882000	1000	1	4.41E-05	0.144	-1.0676582	19	1.9	CCDC201;IGFBP1	Receptor
DMR7:50399001	7	50399001	50401000	2000	1	6.24E-05	0.156	0.5991684	94	4.7	IKZF1;AC124014.1	Transcription
DMR7:51330001	7	51330001	51331000	1000	1	9.05E-05	0.174	0.6505368	5	0.5		
DMR7:62740001	7	62740001	62741000	1000	1	2.06E-05	0.109	-1.076527	8	0.8		
DMR7:64155001	7	64155001	64156000	1000	1	2.58E-05	0.118	-0.8933418	8	0.8	GUSBP6;AC091685.1;VN1R37P	
DMR7:64595001	7	64595001	64596000	1000	1	6.43E-05	0.157	0.5216557	23	2.3	AC016769.4	
DMR7:67885001	7	67885001	67886000	1000	1	2.77E-05	0.121	-0.963737	8	0.8		
DMR7:73078001	7	73078001	73081000	3000	1	4.07E-05	0.141	0.5203943	75	2.5	AC211476.5;AC211476.4;SPDYE9	
DMR7:73106001	7	73106001	73107000	1000	1	2.25E-05	0.113	0.713457	36	3.6	PMS2P6;SPDYE10P	
DMR7:73658001	7	73658001	73661000	3000	1	2.95E-06	0.069	0.5135413	63	2.1	VPS37D	
DMR7:77026001	7	77026001	77028000	2000	1	5.30E-06	0.078	0.5433125	39	1.95	DTX2P1-UPK3BP1-PMS2P11;SPDYE17	
DMR7:91399001	7	91399001	91400000	1000	1	4.28E-06	0.074	-0.9728448	1	0.1	AC079760.2;AC079760.1	
DMR7:92992001	7	92992001	92994000	2000	1	5.55E-05	0.153	-1.0083135	14	0.7		
DMR7:98995001	7	98995001	98997000	2000	1	2.91E-05	0.122	0.5293618	47	2.35	TRRAP;AC004893.2;RNF14P3	Signaling
DMR7:115150001	7	115150001	115152000	2000	1	3.77E-05	0.139	-0.964306	9	0.45	LINC01392	
DMR7:122154001	7	122154001	122155000	1000	1	1.06E-06	0.054	-1.0706318	2	0.2	AASS;AC015983.2;AC006020.1	Metabolism
DMR7:131265001	7	131265001	131266000	1000	1	5.15E-05	0.149	-0.7671637	11	1.1	MKLN1	Signaling
DMR7:139081001	7	139081001	139082000	1000	1	7.54E-05	0.164	0.6204202	8	0.8	ZC3HAV1	Transcription
DMR7:149387001	7	149387001	149389000	2000	1	4.23E-05	0.142	0.5947246	31	1.55	AC004941.1	
DMR7:151920001	7	151920001	151923000	3000	1	8.96E-06	0.088	0.6240269	50	1.667	Y_RNA	
DMR7:151948001	7	151948001	151949000	1000	1	2.01E-05	0.109	0.4656768	9	0.9	AC074257.1;GALNTL5	Metabolism
DMR7:155739001	7	155739001	155740000	1000	1	4.81E-07	0.04	0.6823937	26	2.6	RBM33	
DMR7:157583001	7	157583001	157584000	1000	1	6.72E-05	0.159	0.5492666	18	1.8	PTPRN2;MIR153-2	Signaling
DMR7:157653001	7	157653001	157658000	5000	1	4.85E-05	0.149	0.3851245	179	3.58	PTPRN2	Signaling
DMR7:158084001	7	158084001	158086000	2000	1	8.73E-05	0.173	0.5243226	88	4.4	PTPRN2	Signaling
DMR7:158917001	7	158917001	158918000	1000	1	2.30E-05	0.113	0.5580851	29	2.9	DYNC2I1	
DMR8:2236001	8	2236001	2238000	2000	1	7.05E-06	0.082	0.68991	48	2.4		
DMR8:22799001	8	22799001	22800000	1000	1	1.22E-06	0.054	-1.1392011	11	1.1	AC055854.1;PEBP4	Binding Protein
DMR8:27387001	8	27387001	27388000	1000	1	3.04E-05	0.125	-1.0443533	8	0.8	PTK2B	Signaling
DMR8:30747001	8	30747001	30748000	1000	1	2.91E-05	0.122	0.8292744	14	1.4	UBXN8;HIKESHIP3	Proteolysis
DMR8:35974001	8	35974001	35975000	1000	1	2.14E-05	0.111	-1.1551328	2	0.2	AC124290.1	
DMR8:36669001	8	36669001	36670000	1000	1	9.28E-05	0.176	-0.6902134	4	0.4	AC090809.1	
DMR8:38918001	8	38918001	38919000	1000	1	6.21E-05	0.156	0.4225909	13	1.3	PLEKHA2	
DMR8:40233001	8	40233001	40234000	1000	1	8.31E-05	0.171	-0.9666652	2	0.2		
DMR8:42475001	8	42475001	42476000	1000	1	7.60E-05	0.164	0.5009967	25	2.5	SLC20A2;AC093367.1	Metabolism
DMR8:43066001	8	43066001	43067000	1000	1	8.55E-05	0.171	0.5448174	17	1.7	AC110275.1;FNTA;RNU1-124P	Metabolism
DMR8:47996001	8	47996001	47999000	3000	1	4.28E-05	0.142	0.4892218	68	2.267	UBE2V2	Proteolysis
DMR8:48659001	8	48659001	48660000	1000	1	6.29E-05	0.156	0.7318768	44	4.4	AC022915.2;AC022915.3	
DMR8:69692001	8	69692001	69693000	1000	1	9.77E-05	0.178	-0.6459155	12	1.2	SLCO5A1;RN7SKP29	Metabolism
DMR8:73210001	8	73210001	73212000	2000	1	5.60E-05	0.153	-0.8705159	19	0.95	AC100823.2	
DMR8:90663001	8	90663001	90664000	1000	1	3.76E-05	0.139	-0.7796911	7	0.7	LINC00534;TMEM64;AC106038.1	
DMR8:93633001	8	93633001	93634000	1000	1	2.40E-05	0.116	-0.9132297	10	1	LINC00535	
DMR8:99724001	8	99724001	99725000	1000	1	1.80E-05	0.108	-0.8963137	6	0.6	VPS13B	
DMR8:120738001	8	120738001	120739000	1000	1	8.58E-05	0.171	-0.9079719	2	0.2	SNTB1	Development
DMR8:128041001	8	128041001	128042000	1000	1	1.37E-05	0.098	0.5021793	10	1	PVT1;MIR1207	
DMR8:128419001	8	128419001	128420000	1000	1	5.76E-05	0.154	-0.8096829	7	0.7	LINC00824	
DMR8:130492001	8	130492001	130493000	1000	1	1.64E-05	0.103	-0.9360929	12	1.2		
DMR8:132333001	8	132333001	132335000	2000	1	2.54E-05	0.118	-0.9178379	15	0.75	KCNQ3	Transport
DMR8:132892001	8	132892001	132894000	2000	1	1.26E-06	0.054	0.5128163	28	1.4	TG	Signaling
DMR8:133302001	8	133302001	133303000	1000	1	5.37E-05	0.15	-0.6339922	6	0.6	NDRG1	Transcription
DMR8:137105001	8	137105001	137106000	1000	1	8.17E-05	0.171	-0.8722326	7	0.7	RNU6-144P	
DMR8:141302001	8	141302001	141303000	1000	1	8.87E-06	0.088	0.4534619	16	1.6	SLC45A4;AC011676.3	Metabolism
DMR8:143122001	8	143122001	143125000	3000	1	5.30E-05	0.15	0.6471279	92	3.067		
DMR8:144828001	8	144828001	144829000	1000	1	3.51E-05	0.135	-0.7868867	21	2.1	ZNF7	Transcription

DMR9:826001	9	826001	827000	1000	1	1.12E-05	0.098	0.4473205	33	3.3		
DMR9:1824001	9	1824001	1826000	2000	1	9.92E-05	0.178	-0.9737718	9	0.45		
DMR9:9317001	9	9317001	9318000	1000	1	8.51E-05	0.171	-0.8208323	8	0.8	PTPRD	Signaling
DMR9:18263001	9	18263001	18264000	1000	1	9.04E-05	0.174	-0.813708	11	1.1	ADAMTSL1	Extracellular Matrix
DMR9:20660001	9	20660001	20661000	1000	1	4.71E-05	0.147	-0.793804	4	0.4	FOCAD	
DMR9:22521001	9	22521001	22522000	1000	1	3.57E-05	0.136	-1.0255977	3	0.3		
DMR9:35363001	9	35363001	35364000	1000	1	9.94E-05	0.178	-0.8625876	6	0.6	UNC13B;AL160274.1	Receptor
DMR9:38231001	9	38231001	38232000	1000	1	5.05E-05	0.149	0.7916222	14	1.4		
DMR9:38443001	9	38443001	38444000	1000	1	7.28E-05	0.161	0.6015588	12	1.2	AL390726.5;ARMC8P1	
DMR9:41538001	9	41538001	41539000	1000	1	3.65E-05	0.137	-0.867947	2	0.2		
DMR9:44842001	9	44842001	44844000	2000	1	2.43E-05	0.116	-1.0254251	30	1.5		
DMR9:62450001	9	62450001	62452000	2000	1	1.44E-05	0.099	0.4529057	28	1.4	BX664725.1;LINC01189	
DMR9:62807001	9	62807001	62808000	1000	1	8.38E-05	0.171	-0.8488896	5	0.5	LINC01410;AL512625.3;RNA5SP283	
DMR9:63740001	9	63740001	63741000	1000	1	1.38E-05	0.098	-0.8426812	3	0.3	AL772155.1;AL772155.2	
DMR9:67833001	9	67833001	67835000	2000	1	2.15E-05	0.111	0.5201652	38	1.9	ANKRD20A1	
DMR9:71393001	9	71393001	71394000	1000	1	7.00E-05	0.16	-1.0149764	9	0.9	TRPM3	Receptor
DMR9:79968001	9	79968001	79969000	1000	1	4.97E-06	0.077	-0.8046459	3	0.3	AL161782.1	
DMR9:81956001	9	81956001	81958000	2000	1	2.96E-05	0.122	-0.7288402	7	0.35	AL158154.3;SPATA31D3;SPATA31D2P	
DMR9:92185001	9	92185001	92186000	1000	1	7.32E-05	0.161	0.6014412	16	1.6	PRSS47	
DMR9:94555001	9	94555001	94557000	2000	1	9.60E-07	0.052	0.4343874	45	2.25	PCAT7;FBP2	Metabolism
DMR9:99498001	9	99498001	99499000	1000	1	2.43E-05	0.116	0.7653574	25	2.5	AL359710.1;STX17-AS1	
DMR9:101243001	9	101243001	101244000	1000	1	8.10E-05	0.17	-1.0902142	3	0.3	PLPPR1	
DMR9:108247001	9	108247001	108248000	1000	1	8.13E-05	0.171	0.4287943	6	0.6	AL353742.1	
DMR9:112510001	9	112510001	112512000	2000	1	6.61E-05	0.159	-0.7194631	8	0.4	KIAA1958	EST
DMR9:115149001	9	115149001	115150000	1000	1	1.66E-05	0.103	-0.8492741	5	0.5	DELEC1	
DMR9:116088001	9	116088001	116089000	1000	1	9.97E-06	0.091	-0.9887985	5	0.5		
DMR9:117328001	9	117328001	117329000	1000	1	3.32E-05	0.132	-0.8472098	11	1.1	ASTN2	Unknown
DMR9:118486001	9	118486001	118487000	1000	1	3.53E-05	0.136	0.492496	8	0.8		
DMR9:119196001	9	119196001	119197000	1000	1	6.33E-05	0.157	-1.0103445	5	0.5	BRINP1	
DMR9:119953001	9	119953001	119954000	1000	1	9.52E-06	0.09	-0.8704375	7	0.7		
DMR9:120144001	9	120144001	120146000	2000	1	6.66E-06	0.082	-1.4111936	10	0.5		
DMR9:123972001	9	123972001	123974000	2000	1	2.04E-05	0.109	0.5172247	39	1.95		
DMR9:131847001	9	131847001	131849000	2000	1	1.96E-05	0.109	0.6468872	69	3.45	MED27	
DMR9:133762001	9	133762001	133763000	1000	1	6.53E-05	0.158	0.5522808	20	2	VAV2	Signaling
DMR9:134278001	9	134278001	134280000	2000	1	5.11E-05	0.149	0.4447575	28	1.4		
DMR9:136579001	9	136579001	136582000	3000	2	1.96E-06	0.061	0.5557957	60	2		
DMR9:137544001	9	137544001	137545000	1000	1	9.70E-05	0.178	0.4323931	27	2.7	PNPLA7;MRPL41;DPH7	Metabolism;Translation
DMR10:1746001	10	1746001	1747000	1000	1	4.71E-05	0.147	-0.7924689	18	1.8	ADARB2	Metabolism
DMR10:12279001	10	12279001	12280000	1000	1	6.98E-05	0.16	0.6527116	22	2.2		
DMR10:27660001	10	27660001	27661000	1000	1	8.42E-05	0.171	-0.8657703	17	1.7		
DMR10:69438001	10	69438001	69439000	1000	1	5.98E-05	0.156	-0.8173604	13	1.3	ATP5MC1P7	
DMR10:77579001	10	77579001	77581000	2000	1	1.36E-05	0.098	-0.7961421	26	1.3	KCNMA1;RNA5SP321	Metabolism
DMR10:78792001	10	78792001	78793000	1000	1	5.65E-05	0.154	-0.8927076	13	1.3		
DMR10:79377001	10	79377001	79380000	3000	1	2.28E-05	0.113	0.5965113	82	2.733	ZCCHC24;AL133481.1	
DMR10:86553001	10	86553001	86554000	1000	1	9.88E-05	0.178	-0.7355689	13	1.3		
DMR10:93064001	10	93064001	93065000	1000	1	4.96E-05	0.149	-1.0014807	9	0.9	EXOC6;AL358613.1;CYP26C1;AL358613.3;AL358613.2;CYP26A1	Transport;Metabolism;Electron Transport
DMR10:93474001	10	93474001	93475000	1000	1	4.60E-07	0.04	-0.9007054	6	0.6	MYOF	Cytoskeleton
DMR10:101760001	10	101760001	101761000	1000	1	4.84E-05	0.149	0.8072225	10	1	FGF8	Signaling
DMR10:107691001	10	107691001	107692000	1000	1	9.30E-06	0.09	0.8387696	9	0.9	LINC01435	
DMR10:109269001	10	109269001	109270000	1000	1	4.56E-06	0.075	-1.0707819	7	0.7		
DMR10:109369001	10	109369001	109371000	2000	1	3.48E-05	0.135	-0.7942199	8	0.4		
DMR10:111006001	10	111006001	111007000	1000	1	4.45E-05	0.144	0.8697455	24	2.4	SHOC2	Signaling
DMR10:114108001	10	114108001	114109000	1000	1	1.01E-05	0.091	0.6535932	16	1.6	AC022023.2;RNU6-709P;UBE2V1P5	
DMR10:116350001	10	116350001	116351000	1000	1	8.57E-05	0.171	-0.9229004	4	0.4	CCDC172	
DMR10:118282001	10	118282001	118283000	1000	1	5.15E-06	0.078	0.4888667	6	0.6		
DMR10:120502001	10	120502001	120503000	1000	1	5.65E-07	0.04	-0.8492586	7	0.7	PLPP4	
DMR10:121867001	10	121867001	121868000	1000	1	2.63E-05	0.119	-0.8156815	9	0.9	ATE1	Metabolism
DMR10:127948001	10	127948001	127949000	1000	1	2.45E-05	0.116	-1.0196892	10	1	PTPRE	Signaling
DMR10:132514001	10	132514001	132515000	1000	1	4.55E-05	0.145	0.4795366	7	0.7	AL451069.1;LINC01165	
DMR10:132835001	10	132835001	132836000	1000	1	2.31E-06	0.065	0.7601909	28	2.8	CFAP46	
DMR11:1306001	11	1306001	1307000	1000	1	1.28E-05	0.098	0.6048517	29	2.9	TOLLIP;TOLLIP-AS1	Immune
DMR11:1496001	11	1496001	1497000	1000	1	1.99E-05	0.109	-0.7324227	12	1.2	MOB2	Signaling
DMR11:3143001	11	3143001	3144000	1000	1	4.12E-05	0.141	0.6657484	45	4.5	OSBPL5	Binding Protein

DMR11:3605001	11	3605001	3606000	1000	1	7.93E-05	0.169	-0.8224113	10	1	OR7E117P;TRPC2	
DMR11:4995001	11	4995001	4997000	2000	1	7.77E-05	0.167	-0.7021375	11	0.55	MMP26;OR51L1	Protease
DMR11:13252001	11	13252001	13253000	1000	1	4.08E-05	0.141	-0.9069295	7	0.7		
DMR11:14926001	11	14926001	14927000	1000	1	3.93E-05	0.14	-0.9365664	4	0.4	CALCB	
DMR11:45303001	11	45303001	45304000	1000	1	5.84E-05	0.155	-1.0335894	10	1		
DMR11:45418001	11	45418001	45419000	1000	1	5.34E-05	0.15	-0.8744207	9	0.9	AC103855.3;AC018716.2	
DMR11:46512001	11	46512001	46513000	1000	1	4.17E-06	0.074	-0.8394543	11	1.1	AMBRA1	
DMR11:57102001	11	57102001	57103000	1000	1	8.64E-05	0.171	-0.9606308	3	0.3		
DMR11:57966001	11	57966001	57968000	2000	1	2.71E-05	0.12	0.597377	22	1.1		
DMR11:61547001	11	61547001	61548000	1000	1	1.43E-06	0.056	0.63991	30	3	SYT7;AP003559.1	
DMR11:62811001	11	62811001	62812000	1000	1	5.24E-06	0.078	-0.7827912	3	0.3	NXF1;STX5;AP001160.4;RNU6-118P	Transcription;Transport
DMR11:62930001	11	62930001	62931000	1000	1	6.93E-05	0.16	-0.9144837	5	0.5	CHRM1;RN7SL259P;SLC22A6;AP000438.2	Receptor;Transport
DMR11:71067001	11	71067001	71070000	3000	1	8.33E-05	0.171	0.4712602	28	0.933	SHANK2	Protein Binding
DMR11:74326001	11	74326001	74327000	1000	1	1.09E-05	0.097	-0.9757078	8	0.8	P4HA3-AS1;PGM2L1	Metabolism
DMR11:93979001	11	93979001	93980000	1000	1	2.78E-05	0.121	-0.9866508	2	0.2		
DMR11:96327001	11	96327001	96328000	1000	1	7.10E-05	0.16	0.589606	19	1.9	MAML2	Transcription
DMR11:116081001	11	116081001	116082000	1000	1	9.22E-05	0.175	-0.7030232	9	0.9		
DMR11:116401001	11	116401001	116402000	1000	1	6.88E-05	0.16	0.6376092	11	1.1		
DMR11:117442001	11	117442001	117443000	1000	1	4.16E-05	0.141	0.6386972	19	1.9	DSCAML1	Development
DMR11:117454001	11	117454001	117455000	1000	1	8.30E-05	0.171	-0.7451259	5	0.5	DSCAML1	Development
DMR11:131190001	11	131190001	131191000	1000	1	1.51E-06	0.056	-1.0883272	11	1.1	AP002856.3	
DMR11:133738001	11	133738001	133740000	2000	2	2.11E-05	0.111	0.4912298	15	0.75		
DMR11:134739001	11	134739001	134741000	2000	1	2.60E-05	0.118	0.4672247	26	1.3	LINC02714	
DMR12:4240001	12	4240001	4241000	1000	1	9.84E-05	0.178	-0.8813062	8	0.8	CCND2-AS1	
DMR12:18173001	12	18173001	18175000	2000	2	2.01E-07	0.032	-1.1500679	7	0.35	RERGL	Signaling
DMR12:24297001	12	24297001	24298000	1000	1	2.41E-05	0.116	-0.9543994	15	1.5	SOX5	Transcription
DMR12:42432001	12	42432001	42433000	1000	1	6.50E-05	0.158	-0.8220053	10	1	PPHLN1;AC079601.2	
DMR12:49877001	12	49877001	49881000	4000	1	1.52E-05	0.1	0.5615466	47	1.175	FAIM2	Apoptosis
DMR12:53702001	12	53702001	53704000	2000	1	5.87E-05	0.155	-0.9949206	15	0.75	CALCOCO1	
DMR12:65896001	12	65896001	65897000	1000	1	6.38E-06	0.082	-1.0418705	8	0.8	HMGA2	Transcription
DMR12:66186001	12	66186001	66187000	1000	1	7.39E-05	0.162	-1.0761824	3	0.3	IRAK3	Receptor
DMR12:78911001	12	78911001	78912000	1000	1	1.92E-05	0.109	-0.9401804	5	0.5	SYT1	Transport
DMR12:79370001	12	79370001	79371000	1000	1	8.32E-06	0.087	0.8211747	21	2.1	SYT1;AC027288.3	Transport
DMR12:105602001	12	105602001	105603000	1000	1	1.79E-05	0.107	-0.9260368	7	0.7		
DMR12:107301001	12	107301001	107302000	1000	1	1.43E-06	0.056	-1.1545809	4	0.4		
DMR12:108105001	12	108105001	108106000	1000	1	4.82E-05	0.149	-0.8757162	8	0.8		
DMR12:110405001	12	110405001	110406000	1000	1	9.46E-05	0.178	0.4609128	18	1.8	ANAPC7;AC144548.1	Cell Cycle
DMR12:112712001	12	112712001	112714000	2000	1	1.16E-05	0.098	-0.5380577	18	0.9	RPH3A	Unknown
DMR12:117407001	12	117407001	117408000	1000	1	9.08E-05	0.174	0.5152095	15	1.5	NOS1	Metabolism
DMR12:120346001	12	120346001	120347000	1000	1	4.43E-05	0.144	0.4664775	23	2.3	MSI1	Transcription
DMR12:122093001	12	122093001	122096000	3000	1	6.14E-06	0.082	0.3976216	34	1.133	MLXIP	Transcription
DMR12:122187001	12	122187001	122188000	1000	1	8.34E-05	0.171	0.5631506	20	2	LRRC43	
DMR12:123173001	12	123173001	123174000	1000	1	1.48E-05	0.1	-0.8085797	8	0.8	MPHOSPH9	Cell Cycle
DMR12:125297001	12	125297001	125298000	1000	1	7.91E-05	0.169	-0.7638773	13	1.3	TMEM132B;AC093028.1	Unknown
DMR12:126844001	12	126844001	126845000	1000	1	2.58E-05	0.118	-1.1213243	10	1		
DMR12:130583001	12	130583001	130585000	2000	1	8.63E-05	0.171	0.3968417	16	0.8	RIMBP2	Unknown
DMR12:130758001	12	130758001	130760000	2000	2	1.32E-05	0.098	0.4554502	20	1		
DMR12:131854001	12	131854001	131856000	2000	1	8.33E-05	0.171	0.5441208	70	3.5	MMP17;AC131009.2	Proteolysis
DMR12:131917001	12	131917001	131920000	3000	1	7.29E-05	0.161	0.5356677	125	4.167	ULK1;AC131009.1;PUS1	Development;Metabolism
DMR12:132788001	12	132788001	132789000	1000	1	6.17E-05	0.156	0.6328264	37	3.7	GOLGA3	Golgi
DMR13:18903001	13	18903001	18905000	2000	1	4.82E-05	0.149	0.3711568	38	1.9	SNX19P2;LINC00408	
DMR13:20362001	13	20362001	20363000	1000	1	9.68E-05	0.178	0.6133446	24	2.4		
DMR13:24355001	13	24355001	24358000	3000	2	4.19E-05	0.141	0.5149596	57	1.9		
DMR13:28338001	13	28338001	28339000	1000	1	7.22E-05	0.161	-0.7997922	11	1.1	FLT1	Receptor
DMR13:30816001	13	30816001	30818000	2000	1	2.01E-05	0.109	-0.6584503	26	1.3	LINC00398	
DMR13:31406001	13	31406001	31407000	1000	1	8.48E-05	0.171	-1.0213641	13	1.3		
DMR13:34989001	13	34989001	34990000	1000	1	4.05E-05	0.141	-1.001322	8	0.8	NBEA	Signaling
DMR13:40553001	13	40553001	40554000	1000	1	9.63E-05	0.178	-0.7133325	8	0.8	AL133318.1;FOXO1	Transcription
DMR13:42024001	13	42024001	42025000	1000	1	7.13E-05	0.16	-0.9608333	4	0.4		
DMR13:78029001	13	78029001	78030000	1000	1	1.33E-05	0.098	-1.1926815	6	0.6	OBI1-AS1;LINC00446	
DMR13:86508001	13	86508001	86509000	1000	1	5.13E-05	0.149	-0.7876722	7	0.7		
DMR13:90755001	13	90755001	90757000	2000	1	6.16E-05	0.156	-0.7599091	13	0.65		
DMR13:105093001	13	105093001	105094000	1000	1	2.00E-05	0.109	-1.0081335	2	0.2		
DMR13:107518001	13	107518001	107519000	1000	1	9.21E-05	0.175	-0.6147607	6	0.6	FAM155A	

DMR13:112692001	13	112692001	112694000	2000	1	8.92E-05	0.174	0.5111758	46	2.3	ATP11AUN;AL139384.1;ATP11A	Metabolism
DMR13:113774001	13	113774001	113776000	2000	1	7.98E-05	0.17	0.4820144	45	2.25	TMEM255B	
DMR14:19834001	14	19834001	19835000	1000	1	8.77E-05	0.173	-1.1923676	9	0.9	OR4N2	
DMR14:20460001	14	20460001	20461000	1000	1	4.41E-05	0.144	-0.8046175	16	1.6	OSGEP;AL355075.2;APEX1;PIP4P1;PNP	Protease;DNA Repair;Metabolism
DMR14:35113001	14	35113001	35114000	1000	1	8.51E-05	0.171	-0.9381657	6	0.6	FAM177A1;PPP2R3C;PRORP;AL121594.1	Signaling
DMR14:61693001	14	61693001	61694000	1000	1	9.83E-05	0.178	0.7701814	19	1.9	HIF1A-AS1;HIF1A	Transcription
DMR14:64766001	14	64766001	64767000	1000	1	1.96E-05	0.109	0.4905366	22	2.2	SPTB	Cytoskeleton
DMR14:74298001	14	74298001	74299000	1000	1	2.23E-05	0.113	0.6081094	23	2.3	ABCD4;AC005519.1;VRTN;SUB1P2	Transport
DMR14:92436001	14	92436001	92438000	2000	1	5.99E-06	0.082	-1.0523058	23	1.15	SLC24A4	Metabolism
DMR15:19896001	15	19896001	19897000	1000	1	1.20E-06	0.054	-1.2645607	5	0.5	AC138701.1;AC138701.2	
DMR15:20187001	15	20187001	20189000	2000	1	6.36E-05	0.157	-1.2689552	7	0.35		
DMR15:20222001	15	20222001	20224000	2000	1	5.65E-06	0.078	-0.7718539	15	0.75	RHPN2P1	
DMR15:20387001	15	20387001	20388000	1000	1	4.58E-05	0.145	-1.4207019	2	0.2	HERC2P3	
DMR15:20597001	15	20597001	20598000	1000	1	2.21E-05	0.113	-1.0709116	12	1.2	SPATA31E2P	
DMR15:21123001	15	21123001	21124000	1000	1	3.23E-05	0.13	-1.0928681	8	0.8	AC126335.1	
DMR15:21335001	15	21335001	21337000	2000	1	6.16E-05	0.156	-0.9719842	20	1	AC068446.2;AC060814.4;AC060814.3;LONRF2P4	
DMR15:21645001	15	21645001	21646000	1000	1	2.86E-05	0.122	-1.0500473	7	0.7	LINC02203;AC135068.1;AC135068.4	
DMR15:22767001	15	22767001	22771000	4000	1	1.62E-05	0.103	0.5845569	88	2.2	AC138649.1;AC011767.1;NIPA1	Development
DMR15:40403001	15	40403001	40405000	2000	1	8.22E-05	0.171	0.5214034	36	1.8	KNSTRN;IVD	Metabolism
DMR15:41965001	15	41965001	41966000	1000	1	6.60E-05	0.159	0.712119	13	1.3	EHD4;PLA2G4E-AS1	Signaling
DMR15:42182001	15	42182001	42183000	1000	1	6.86E-06	0.082	-0.9618579	9	0.9	VPS39	Proteolysis
DMR15:45160001	15	45160001	45161000	1000	1	5.04E-05	0.149	-0.5744004	13	1.3	DUOX1;AC051619.5;SHF;AC051619.3	
DMR15:49861001	15	49861001	49862000	1000	1	2.69E-05	0.12	-0.9462598	6	0.6	ATP8B4	Transport
DMR15:66054001	15	66054001	66055000	1000	1	7.33E-05	0.161	-0.5912419	7	0.7	MEGF11	Extracellular Matrix
DMR15:79135001	15	79135001	79137000	2000	1	6.95E-05	0.16	-0.8097482	11	0.55	ANKRD34C-AS1	
DMR15:80444001	15	80444001	80446000	2000	1	5.17E-05	0.149	0.433505	28	1.4	ARNT2;AC016705.1;AC016705.3	Transcription
DMR15:82487001	15	82487001	82488000	1000	1	8.80E-05	0.173	0.6537697	17	1.7	CSPG4P10;GOLGA2P10	
DMR15:86057001	15	86057001	86058000	1000	1	8.10E-05	0.17	-0.7381629	7	0.7		
DMR15:87648001	15	87648001	87649000	1000	1	6.64E-05	0.159	0.825983	9	0.9	AC020687.1;AC103871.1	
DMR15:93870001	15	93870001	93871000	1000	1	7.14E-06	0.082	-0.7178083	4	0.4	LINC01579;LINC02207	
DMR15:97064001	15	97064001	97065000	1000	1	3.71E-05	0.138	-1.2306237	5	0.5		
DMR15:97882001	15	97882001	97883000	1000	1	1.25E-05	0.098	-0.8631797	8	0.8	LINC00923;AC024651.2	
DMR15:98537001	15	98537001	98538000	1000	1	5.36E-05	0.15	0.4918937	11	1.1	FAM169B	
DMR15:98592001	15	98592001	98593000	1000	1	1.88E-05	0.109	0.540337	28	2.8		
DMR15:100717001	15	100717001	100720000	3000	1	1.33E-05	0.098	-1.262755	30	1	AC087762.1	
DMR16:5451001	16	5451001	5452000	1000	1	1.45E-05	0.099	-0.7504102	13	1.3	RBFOX1	Unknown
DMR16:5564001	16	5564001	5565000	1000	1	1.24E-05	0.098	-0.8910777	11	1.1	RBFOX1	Unknown
DMR16:10581001	16	10581001	10582000	1000	1	9.62E-05	0.178	0.5931457	11	1.1	EMP2;AC027277.2	Cytoskeleton
DMR16:11131001	16	11131001	11132000	1000	1	5.56E-05	0.153	0.6116409	15	1.5	CLEC16A	
DMR16:15061001	16	15061001	15062000	1000	1	2.51E-06	0.067	-1.1086643	13	1.3	PDXDC1;NTAN1;RRN3	Metabolism;Transcription
DMR16:15197001	16	15197001	15198000	1000	1	6.43E-05	0.157	-0.8481795	10	1		
DMR16:19321001	16	19321001	19322000	1000	1	9.90E-05	0.178	-0.5455039	5	0.5	CLEC19A;AC130456.1	
DMR16:31693001	16	31693001	31695000	2000	1	1.71E-05	0.105	0.4796592	27	1.35	AC074050.1;AC074050.2;CLUHP3;AC074050.4	
DMR16:33506001	16	33506001	33510000	4000	1	6.42E-05	0.157	-0.8004669	33	0.825	AC136944.5	
DMR16:33625001	16	33625001	33626000	1000	1	3.60E-06	0.071	-1.1868946	1	0.1	AC142384.1	
DMR16:33978001	16	33978001	33979000	1000	1	3.16E-06	0.069	-0.8899743	18	1.8	AC140658.1;AC133561.1;BCAP31P1	
DMR16:54472001	16	54472001	54473000	1000	1	5.66E-06	0.078	-1.0896656	7	0.7		
DMR16:67816001	16	67816001	67817000	1000	1	3.34E-05	0.132	0.6894721	39	3.9	RANBP10;TSNAXIP1	Transport
DMR16:68618001	16	68618001	68619000	1000	1	9.58E-05	0.178	0.4823365	24	2.4		
DMR16:72389001	16	72389001	72390000	1000	1	8.25E-06	0.087	-0.9296976	5	0.5	LINC01572	
DMR16:73169001	16	73169001	73172000	3000	1	7.56E-06	0.086	-0.7554001	44	1.467	ZFHX3	Transcription
DMR16:81626001	16	81626001	81628000	2000	1	1.14E-06	0.054	0.4919693	35	1.75	CMIP;AC092135.2;AC092135.1;AC092135.3	Signaling
DMR16:85400001	16	85400001	85402000	2000	1	1.24E-08	0.007	0.5213576	55	2.75	GSE1	Transcription
DMR16:86587001	16	86587001	86589000	2000	1	7.18E-05	0.16	0.444851	11	0.55	FOXL1;AC009108.4	Transcription

DMR16:87734001	16	87734001	87736000	2000	1	6.49E-05	0.158	0.484472	63	3.15	KLHDC4;AC010536.2	Unknown
DMR16:89682001	16	89682001	89686000	4000	1	2.72E-05	0.12	0.6800727	88	2.2	AC010538.1;CDK10;LINC02166	Signaling
DMR17:1072001	17	1072001	1075000	3000	1	6.13E-05	0.156	0.7959911	98	3.267	ABR	Signaling
DMR17:4022001	17	4022001	4023000	1000	1	6.79E-05	0.159	-0.7087108	15	1.5	ZZEF1	Transcription
DMR17:4262001	17	4262001	4263000	1000	1	3.84E-06	0.072	0.6723368	36	3.6	ANKFY1;AC087742.1;UBE2G1	Metabolism
DMR17:6239001	17	6239001	6241000	2000	1	2.38E-05	0.116	0.4567056	40	2		
DMR17:11926001	17	11926001	11927000	1000	1	6.04E-05	0.156	-0.8388053	9	0.9	DNAH9	Cytoskeleton
DMR17:18725001	17	18725001	18727000	2000	1	3.33E-06	0.07	-1.0592141	20	1	TRIM16L	Metabolism
DMR17:20505001	17	20505001	20506000	1000	1	6.68E-05	0.159	0.6313769	23	2.3	KRT16P3;AC015818.7;KRT17P6	
DMR17:22090001	17	22090001	22093000	3000	1	5.45E-05	0.152	0.4145191	83	2.767	UBBP4	
DMR17:28265001	17	28265001	28268000	3000	2	9.58E-06	0.09	0.641514	87	2.9	AC061975.2;AC061975.4;AC061975.1;AC061975.5;AC061975.3;KRT18P55	
DMR17:29892001	17	29892001	29894000	2000	1	4.87E-05	0.149	-0.9160243	7	0.35	SSH2;AC104982.1	Signaling
DMR17:30196001	17	30196001	30197000	1000	1	3.44E-06	0.07	-1.3005652	8	0.8	NSRP1;SLC6A4;AC104984.1	Transcription;Transport
DMR17:37360001	17	37360001	37362000	2000	1	1.36E-05	0.098	-1.1581736	28	1.4	ACACA	Metabolism
DMR17:37988001	17	37988001	37989000	1000	1	8.52E-05	0.171	-0.737408	34	3.4	TBC1D3L	
DMR17:38296001	17	38296001	38297000	1000	1	9.25E-05	0.175	0.6342416	57	5.7	MRPL45	Translation
DMR17:41962001	17	41962001	41963000	1000	1	4.28E-05	0.142	-0.8975816	15	1.5	TTC25;CNP	Metabolism
DMR17:45890001	17	45890001	45891000	1000	1	8.00E-05	0.17	-0.7657365	5	0.5	MAPT-AS1;MAPT;MAPT-IT1	Cytoskeleton
DMR17:47041001	17	47041001	47042000	1000	1	2.77E-06	0.068	0.9411203	17	1.7	AC005670.2;LRRC37A17P;AC005670.3;RN7SL270P	
DMR17:49490001	17	49490001	49491000	1000	1	3.90E-05	0.14	-0.6546255	18	1.8	AC015656.1;NGFR	Receptor
DMR17:61344001	17	61344001	61346000	2000	1	4.86E-05	0.149	-0.6774319	27	1.35	BCAS3;AC005746.3	Transcription
DMR17:61888001	17	61888001	61889000	1000	1	5.24E-05	0.15	0.5233291	19	1.9	INTS2	Translation
DMR17:74465001	17	74465001	74467000	2000	1	3.92E-06	0.073	0.4906128	45	2.25	CD300A	Receptor
DMR17:77215001	17	77215001	77217000	2000	1	2.40E-05	0.116	0.7308332	53	2.65	SEC14L1	Metabolism
DMR17:79462001	17	79462001	79465000	3000	1	7.09E-05	0.16	0.645131	105	3.5	RBFOX3	
DMR17:79834001	17	79834001	79835000	1000	1	4.95E-06	0.077	0.7432347	90	9	LINC01977;CBX4	Epigenetic
DMR17:81300001	17	81300001	81302000	2000	1	7.16E-05	0.16	0.556242	68	3.4	SLC38A10;LINC00482;TMEM105	Metabolism
DMR17:81792001	17	81792001	81794000	2000	1	4.31E-05	0.143	0.3907335	20	1		
DMR17:82029001	17	82029001	82031000	2000	1	7.41E-05	0.162	0.8490412	118	5.9	CENPX;LRRC45;RAC3;DCXR;AC137723.1;DCXR-DT	Signaling;Metabolism
DMR18:5518001	18	5518001	5519000	1000	1	1.58E-05	0.102	0.5207781	17	1.7	EPB41L3	Cytoskeleton
DMR18:5985001	18	5985001	5986000	1000	1	8.07E-05	0.17	-0.7625928	6	0.6	AP001021.1;AP001021.3;L3MBTL4	Epigenetic
DMR18:6296001	18	6296001	6297000	1000	1	9.82E-05	0.178	-0.6689197	5	0.5	L3MBTL4	Epigenetic
DMR18:7000001	18	7000001	7001000	1000	1	2.29E-06	0.065	-1.0389761	12	1.2	LAMA1	Extracellular Matrix
DMR18:12550001	18	12550001	12551000	1000	1	3.85E-05	0.14	-0.6003774	9	0.9	SPIRE1	Cytoskeleton
DMR18:27053001	18	27053001	27054000	1000	1	1.42E-05	0.098	-0.7467242	2	0.2	AQP4-AS1;CHST9	Metabolism
DMR18:27999001	18	27999001	2.80E+07	1000	1	3.19E-06	0.069	-1.0687914	6	0.6	CDH2	Extracellular Matrix
DMR18:34090001	18	34090001	34091000	1000	1	5.15E-05	0.149	-0.8451282	2	0.2	NOL4	Translation
DMR18:34629001	18	34629001	34630000	1000	1	1.11E-05	0.098	-0.9872138	7	0.7	DTNA	Cytoskeleton
DMR18:37075001	18	37075001	37076000	1000	1	4.90E-05	0.149	-0.7944174	4	0.4	KIAA1328	EST
DMR18:44610001	18	44610001	44611000	1000	1	4.12E-05	0.141	-1.1945379	1	0.1		
DMR18:46436001	18	46436001	46438000	2000	1	5.85E-07	0.04	-1.1842229	14	0.7	RNF165	Development
DMR18:51506001	18	51506001	51507000	1000	1	4.84E-05	0.149	-1.0110779	3	0.3	LINC01630	
DMR18:57103001	18	57103001	57104000	1000	1	4.55E-05	0.145	-0.818734	8	0.8		
DMR18:63091001	18	63091001	63093000	2000	1	2.67E-06	0.067	0.5027536	23	1.15		
DMR18:63151001	18	63151001	63152000	1000	1	6.32E-06	0.082	0.6827114	13	1.3	BCL2;AC022726.1	Signaling
DMR18:67231001	18	67231001	67232000	1000	1	9.71E-05	0.178	0.6336959	14	1.4		
DMR18:70022001	18	70022001	70024000	2000	1	9.57E-05	0.178	-0.6704664	13	0.65	RTTN	Development
DMR18:76858001	18	76858001	76860000	2000	1	9.07E-05	0.174	0.4416984	27	1.35	ZNF236	Transcription
DMR18:79512001	18	79512001	79513000	1000	1	6.56E-05	0.158	0.6506644	53	5.3	NFATC1;AC018445.2	Transcription
DMR19:616001	19	616001	618000	2000	1	8.13E-06	0.087	0.6478925	187	9.35	HCN2;POLRMT	Metabolism;Transcription
DMR19:1114001	19	1114001	1115000	1000	1	5.74E-05	0.154	0.5907323	42	4.2	GPX4;SBNO2	Metabolism;Transcription
DMR19:1209001	19	1209001	1210000	1000	1	3.56E-05	0.136	0.5788786	27	2.7	STK11;HMGB2P1	Signaling
DMR19:2568001	19	2568001	2569000	1000	1	7.57E-05	0.164	0.5088567	10	1	GNG7	Signaling
DMR19:8407001	19	8407001	8408000	1000	1	2.83E-05	0.122	-0.6598351	14	1.4	RAB11B;MARCHF2	Signaling
DMR19:9021001	19	9021001	9022000	1000	1	2.21E-06	0.065	0.5726401	19	1.9	BOLA3P2	
DMR19:14676001	19	14676001	14679000	3000	1	1.57E-05	0.102	0.4804208	68	2.267	ADGRE3	
DMR19:14744001	19	14744001	14746000	2000	1	9.22E-05	0.175	0.4904076	38	1.9	ADGRE2	
DMR19:16736001	19	16736001	16737000	1000	1	2.50E-05	0.118	-0.7521787	8	0.8	NWD1	Unknown

DMR19:28759001	19	28759001	28760000	1000	1	1.41E-05	0.098	-0.8637165	2	0.2		
DMR19:41585001	19	41585001	41586000	1000	1	8.88E-05	0.174	-0.9467	6	0.6	CEACAM21	
DMR19:47490001	19	47490001	47491000	1000	1	1.14E-05	0.098	0.5179953	14	1.4	KPTN;NAPA-AS1;NAPA;AC073548.1	Development
DMR19:47864001	19	47864001	47865000	1000	1	7.35E-05	0.161	0.5133693	17	1.7	TPRX2P;LINC01595;SULT2A1	Metabolism
DMR19:53723001	19	53723001	53724000	1000	1	6.17E-05	0.156	0.6145271	21	2.1	MIR519D;MIR521-2;RNU6-803P;MIR520D;MIR517B;MIR520G;MIR516B2;MIR526A2;MIR518E;MIR518A1;RNU6-980P	
DMR19:56589001	19	56589001	56590000	1000	1	1.98E-05	0.109	-0.9478487	11	1.1	ZNF470;SIGLEC31P;ZNF71;ZIM2-AS1	Transcription
DMR20:13198001	20	13198001	13199000	1000	1	6.79E-06	0.082	-0.7646997	3	0.3		
DMR20:16858001	20	16858001	16859000	1000	1	2.70E-05	0.12	-0.7352461	7	0.7	AL121892.1	
DMR20:18627001	20	18627001	18628000	1000	1	2.24E-05	0.113	-0.8153961	16	1.6	AL121900.1;DTD1	Transcription
DMR20:19751001	20	19751001	19757000	6000	1	5.91E-05	0.155	0.4644174	116	1.933	AL121761.1;RIN2	Signaling
DMR20:31266001	20	31266001	31267000	1000	1	3.60E-05	0.136	-0.7773207	6	0.6	DEFB115;DKKL1P1	
DMR20:37266001	20	37266001	37267000	1000	1	1.32E-05	0.098	-1.0873809	12	1.2	GHRH	Signaling
DMR20:42124001	20	42124001	42125000	1000	1	3.51E-05	0.135	-0.8246334	18	1.8	PTPRT	Receptor
DMR20:43261001	20	43261001	43262000	1000	1	2.93E-05	0.122	-0.9470345	9	0.9		
DMR20:48010001	20	48010001	48012000	2000	1	2.90E-05	0.122	0.4255105	12	0.6	AL139351.2	
DMR20:48013001	20	48013001	48015000	2000	1	1.28E-05	0.098	-0.7586276	23	1.15	AL139351.2	
DMR20:48158001	20	48158001	48159000	1000	1	6.71E-06	0.082	-1.0738885	7	0.7		
DMR20:48892001	20	48892001	48893000	1000	1	7.37E-05	0.161	-0.6787441	10	1		
DMR20:51229001	20	51229001	51230000	1000	1	8.24E-05	0.171	0.5762014	19	1.9		
DMR20:54134001	20	54134001	54136000	2000	1	1.31E-05	0.098	-1.151683	10	0.5		
DMR20:60705001	20	60705001	60706000	1000	1	3.57E-05	0.136	-0.8864626	7	0.7		
DMR20:62160001	20	62160001	62162000	2000	1	6.25E-05	0.156	0.5505943	69	3.45	SS18L1	Transcription
DMR20:62398001	20	62398001	62399000	1000	1	9.82E-05	0.178	0.5654331	25	2.5	RPS21;CABLES2;AL121832.3	Translation;Cell Cycle
DMR20:63424001	20	63424001	63427000	3000	1	2.50E-06	0.067	0.6979096	104	3.467	KCNQ2;AL353658.1	Metabolism
DMR21:6070001	21	6070001	6071000	1000	1	2.88E-05	0.122	0.4462019	15	1.5	LINC01669	
DMR21:10411001	21	10411001	10416000	5000	1	9.91E-05	0.178	0.3489166	99	1.98	BAGE2	
DMR21:25526001	21	25526001	25528000	2000	1	5.36E-05	0.15	-0.6923896	14	0.7	AP000221.1	
DMR21:26230001	21	26230001	26234000	4000	1	4.59E-05	0.145	0.4880212	49	1.225		
DMR21:28446001	21	28446001	28447000	1000	1	8.56E-05	0.171	-1.1906545	5	0.5	AF165147.1	
DMR21:36341001	21	36341001	36342000	1000	1	6.67E-05	0.159	-0.8379575	8	0.8	MORC3	Transcription
DMR21:36640001	21	36640001	36641000	1000	1	2.33E-05	0.114	-1.053671	19	1.9	AP000696.1	
DMR21:42662001	21	42662001	42664000	2000	2	9.94E-06	0.091	0.4828704	51	2.55	PDE9A	Signaling
DMR21:43159001	21	43159001	43161000	2000	1	2.06E-05	0.109	0.5466364	66	3.3	AP001631.2;AP001631.1;CRYAA	Unknown
DMR21:44810001	21	44810001	44812000	2000	1	7.18E-05	0.16	0.5774084	38	1.9	UBE2G2;LINC01424;SUMO3	Metabolism
DMR22:10839001	22	10839001	10840000	1000	1	8.28E-05	0.171	-1.2401693	1	0.1		
DMR22:15871001	22	15871001	15873000	2000	1	5.44E-06	0.078	-0.9654748	12	0.6	NBEAP3	
DMR22:18350001	22	18350001	18352000	2000	1	9.48E-05	0.178	0.6919372	35	1.75	GGTLC5P;FAM230J	
DMR22:23986001	22	23986001	23989000	3000	2	1.53E-06	0.056	1.1245919	39	1.3	DDT;AC253536.7;AC253536.1;AC253536.5;GSTT2;GSTT4	Metabolism
DMR22:26389001	22	26389001	26390000	1000	1	6.46E-05	0.158	-0.8638393	10	1	SEZ6L;RNA5SP495	Receptor
DMR22:27649001	22	27649001	27650000	1000	1	4.88E-05	0.149	0.4162252	14	1.4		
DMR22:29395001	22	29395001	29397000	2000	1	9.48E-06	0.09	-0.8806338	23	1.15	AP1B1;AC002059.3	Metabolism
DMR22:33883001	22	33883001	33884000	1000	1	6.14E-05	0.156	0.5080398	15	1.5	LARGE1	
DMR22:35633001	22	35633001	35634000	1000	1	1.81E-05	0.108	0.639189	24	2.4	MB;AL049747.1	Binding Protein
DMR22:36824001	22	36824001	36826000	2000	1	7.45E-05	0.162	0.4351114	31	1.55	PVALB;Z82185.1	Receptor
DMR22:37125001	22	37125001	37126000	1000	1	8.08E-05	0.17	-0.8335123	11	1.1	IL2RB	
DMR22:39117001	22	39117001	39118000	1000	1	5.42E-05	0.152	0.7533022	22	2.2	CBX7;COX5BP7	Transcription
DMR22:41935001	22	41935001	41936000	1000	1	1.20E-05	0.098	0.9116198	17	1.7	TNFRSF13C;CENPM	
DMR22:45890001	22	45890001	45891000	1000	1	3.67E-05	0.138	0.6410695	70	7	BX324167.1	
DMR22:48269001	22	48269001	48270000	1000	1	8.63E-05	0.171	0.3951172	19	1.9	AL008720.1;MIR3201	
DMR22:48581001	22	48581001	48586000	5000	1	1.71E-05	0.105	0.4591569	91	1.82	TAFA5	
DMRX:1142001	X	1142001	1145000	3000	1	1.29E-05	0.098	0.6719909	66	2.2		
DMRX:12798001	X	12798001	12799000	1000	1	3.77E-05	0.139	-0.7896141	9	0.9	PRPS2	
DMRX:18883001	X	18883001	18884000	1000	1	6.03E-05	0.156	-0.7923964	15	1.5	PHKA2-AS1;PHKA2	Signaling
DMRX:20625001	X	20625001	20626000	1000	1	1.52E-07	0.032	-1.0186935	4	0.4		
DMRX:38349001	X	38349001	38350000	1000	1	9.69E-05	0.178	-0.8165465	5	0.5	AF241726.1;OTC	Metabolism
DMRX:39131001	X	39131001	39132000	1000	1	6.91E-05	0.16	-0.8311351	6	0.6		
DMRX:40041001	X	40041001	40042000	1000	1	5.13E-06	0.078	-0.8523478	4	0.4	BCOR	
DMRX:48038001	X	48038001	48039000	1000	1	6.75E-06	0.082	-1.0430505	5	0.5	ZNF630	Transcription
DMRX:52784001	X	52784001	52785000	1000	1	2.09E-06	0.064	-1.0310395	9	0.9	SSX2B;AC244505.4	Transcription
DMRX:56457001	X	56457001	56458000	1000	1	4.67E-05	0.147	-0.860616	4	0.4		

DMRX:66791001	X	66791001	66793000	2000	1	1.83E-05	0.108	-0.9464639	13	0.65		
DMRX:68698001	X	68698001	68699000	1000	1	3.69E-05	0.138	-0.8912648	10	1	STARD8	Signaling
DMRX:73905001	X	73905001	73906000	1000	1	8.01E-06	0.087	-1.2377745	6	0.6		
DMRX:74165001	X	74165001	74167000	2000	1	5.30E-05	0.15	0.493174	92	4.6	FTX;MKRN5P;ATP5MDP1	
DMRX:77213001	X	77213001	77214000	1000	1	2.03E-05	0.109	-0.9922755	9	0.9		
DMRX:109534001	X	109534001	109536000	2000	1	3.95E-05	0.14	0.5242736	25	1.25	NXT2	Transcription
DMRX:115843001	X	115843001	115884000	41000	3	1.53E-05	0.1	0.6633671	2553	6.227	DANT2;DANT1	
DMRX:120444001	X	120444001	120445000	1000	1	4.10E-05	0.141	-0.6479939	6	0.6	LAMP2	Cytoskeleton
DMRX:120854001	X	120854001	120855000	1000	1	5.14E-05	0.149	0.7569382	11	1.1		
DMRX:129302001	X	129302001	129304000	2000	1	1.96E-05	0.109	-1.2248557	8	0.4	AL008633.1	
DMRX:138940001	X	138940001	138941000	1000	1	1.41E-05	0.098	-0.9468551	5	0.5	FGF13	Growth Factors & Cytokines
DMRX:140113001	X	140113001	140115000	2000	1	4.54E-05	0.145	-1.184274	14	0.7		
DMRX:145853001	X	145853001	145854000	1000	1	8.75E-05	0.173	0.4739844	16	1.6		
DMRX:154445001	X	154445001	154448000	3000	1	2.29E-05	0.113	-0.8797222	70	2.333	ATP6AP1;GDI1;FAM50A;MIR6858	Transport;Signaling;Unknown
DMRX:155457001	X	155457001	155461000	4000	1	9.12E-05	0.174	0.885542	278	6.95	F8A3;MIR1184-3;H2AB3;TMLHE-AS1;BX571846.1	Immune
