# Supplementary material for: Epigenome association study for DNA methylation biomarkers in buccal and monocyte cells for female rheumatoid arthritis
Source: Sci Rep. 2021 Dec 10;11:23789. doi: 10.1038/s41598-021-03170-6 (PMC8664902; doi:10.1038/s41598-021-03170-6)
Supplement: Supplementary file 5 — Supplementary Table S3. [file 41598_2021_3170_MOESM5_ESM.pdf]

Supplemental Table S3
DMR Table RA AA Buccal 1e-04

DMR Name	Chr	Start	Stop	Length	# Sig Win	minP	Min FDR	maxLFC	CpG #	CpG Density	Gene Annotation	Gene Category
DMR1:2121001	1	2121001	2125000	4000	2	3.51E-05	0.438	1.4447084	228	5.7	PRKCZ	Signaling
DMR1:4653001	1	4653001	4654000	1000	1	2.77E-05	0.435	-0.8018869	34	3.4	AJAP1	
DMR1:5484001	1	5484001	5486000	2000	1	4.47E-05	0.455	-0.6483865	28	1.4	Z98259.3;Z98259.2;Z98259.1	
DMR1:5946001	1	5946001	5947000	1000	1	6.21E-05	0.471	-0.8265147	11	1.1	NPHP4	Development
DMR1:7342001	1	7342001	7344000	2000	1	3.74E-05	0.447	-1.025064	21	1.05	CAMTA1	Transcription
DMR1:9423001	1	9423001	9425000	2000	1	9.59E-05	0.518	-0.9230418	31	1.55	BX323043.1;LINC02606	
DMR1:17728001	1	17728001	17729000	1000	1	6.87E-05	0.471	-0.8233112	11	1.1	LINC02810	
DMR1:24932001	1	24932001	24933000	1000	1	5.96E-05	0.471	-0.9948062	65	6.5	RUNX3	Transcription
DMR1:30399001	1	30399001	30401000	2000	1	2.88E-05	0.438	-0.6324666	24	1.2	AL161638.2	
DMR1:30598001	1	30598001	30599000	1000	1	1.38E-05	0.408	-0.7880724	9	0.9		
DMR1:53547001	1	53547001	53548000	1000	1	2.92E-05	0.438	-0.7177498	17	1.7	GLIS1	Transcription
DMR1:77926001	1	77926001	77927000	1000	1	5.19E-05	0.471	0.7580559	13	1.3	NEXN	
DMR1:91671001	1	91671001	91672000	1000	1	7.02E-05	0.471	0.5818499	10	1	TGFBR3	Receptor
DMR1:100574001	1	100574001	100575000	1000	1	9.84E-05	0.52	-1.0470236	3	0.3		
DMR1:110116001	1	110116001	110118000	2000	1	4.14E-05	0.453	-0.8833819	22	1.1	LINC01397;UBL4B	Transcription
DMR1:112284001	1	112284001	112285000	1000	1	6.69E-05	0.471	-0.8835104	5	0.5	LINC02884	
DMR1:115112001	1	115112001	115115000	3000	1	3.42E-06	0.226	-1.1515747	49	1.633	LINC01765	
DMR1:119194001	1	119194001	119195000	1000	1	1.52E-05	0.408	0.6408775	7	0.7	WARS2-AS1	
DMR1:121261001	1	121261001	121262000	1000	1	5.88E-05	0.471	0.5528416	13	1.3	SRGAP2C	
DMR1:148126001	1	148126001	148127000	1000	1	5.27E-05	0.471	-0.7097422	17	1.7	NBPF11;PFN1P4	
DMR1:154578001	1	154578001	154579000	1000	1	2.30E-05	0.425	-1.0785862	14	1.4	CHRNB2;AL592078.1;ADAR	Receptor;Transcription
DMR1:155091001	1	155091001	155092000	1000	1	1.46E-05	0.408	-1.1133601	31	3.1	AL691442.1;EFNA3	Signaling
DMR1:158901001	1	158901001	158902000	1000	1	3.09E-05	0.438	-1.1402692	4	0.4		
DMR1:168100001	1	168100001	168101000	1000	1	5.25E-05	0.471	-0.8337965	8	0.8	GPR161	Receptor
DMR1:170449001	1	170449001	170450000	1000	1	5.00E-05	0.471	-1.0270653	12	1.2		
DMR1:173949001	1	173949001	173950000	1000	1	1.08E-05	0.378	0.6056604	11	1.1	RC3H1	
DMR1:180253001	1	180253001	180254000	1000	1	5.42E-06	0.268	-0.791831	17	1.7	LHX4	Transcription
DMR1:185830001	1	185830001	185831000	1000	1	8.90E-05	0.509	0.5454488	7	0.7	HMCN1	Immune
DMR1:196865001	1	196865001	196866000	1000	1	8.22E-05	0.493	0.7188718	8	0.8	BX248415.1	
DMR1:197728001	1	197728001	197730000	2000	1	6.52E-05	0.471	0.5344748	8	0.4	DENND1B;AL365258.2	
DMR1:229789001	1	229789001	229790000	1000	1	3.98E-05	0.451	-0.7534986	7	0.7		
DMR2:10529001	2	10529001	10531000	2000	1	3.23E-05	0.438	-0.8477636	22	1.1		
DMR2:11803001	2	11803001	11804000	1000	1	3.23E-05	0.438	-0.7921737	11	1.1	LPIN1	Development
DMR2:15283001	2	15283001	15284000	1000	1	6.37E-05	0.471	-0.7688298	12	1.2	NBAS	Unknown
DMR2:16118001	2	16118001	16119000	1000	1	1.93E-06	0.213	-1.366401	9	0.9		
DMR2:16645001	2	16645001	16646000	1000	1	9.64E-05	0.518	-0.866754	13	1.3	CYRIA	
DMR2:19361001	2	19361001	19362000	1000	1	5.71E-05	0.471	-0.8441574	67	6.7	OSR1	Development
DMR2:20029001	2	20029001	20031000	2000	1	6.88E-05	0.471	-1.0321478	10	0.5	LAPTM4A	Transport
DMR2:20463001	2	20463001	20465000	2000	1	7.80E-05	0.481	-0.8424459	14	0.7		
DMR2:25834001	2	25834001	25835000	1000	1	3.12E-05	0.438	0.7319194	16	1.6	ASXL2	
DMR2:31928001	2	31928001	31929000	1000	1	4.04E-05	0.452	0.6065213	9	0.9	MEMO1;DPY30	Signaling
DMR2:44045001	2	44045001	44046000	1000	1	2.77E-06	0.217	-0.6522557	8	0.8		
DMR2:46083001	2	46083001	46084000	1000	1	7.84E-05	0.481	-0.9316341	12	1.2	PRKCE;AC017006.2	Binding Protein
DMR2:46986001	2	46986001	46987000	1000	1	1.97E-05	0.408	-1.2457608	22	2.2	TTC7A	Metabolism
DMR2:61047001	2	61047001	61048000	1000	1	4.98E-05	0.471	0.6727511	14	1.4	PEX13	Signaling
DMR2:71480001	2	71480001	71482000	2000	1	4.11E-05	0.453	-1.2996944	18	0.9	DYSF	Transport
DMR2:77820001	2	77820001	77821000	1000	1	5.35E-05	0.471	0.6566927	8	0.8	AC012494.1	
DMR2:84317001	2	84317001	84318000	1000	1	4.39E-05	0.454	1.1550221	8	0.8	AC106874.1	
DMR2:119134001	2	119134001	119136000	2000	1	1.10E-05	0.378	-0.7851328	33	1.65		
DMR2:121044001	2	121044001	121045000	1000	1	2.85E-06	0.217	-1.1462577	9	0.9	Y_RNA	
DMR2:123447001	2	123447001	123448000	1000	1	2.05E-05	0.409	0.7378754	6	0.6	AC073409.2;AC073409.1	
DMR2:130498001	2	130498001	130499000	1000	1	8.55E-05	0.501	-0.7592205	10	1	POTEI;RNU6-473P	
DMR2:136966001	2	136966001	136967000	1000	1	1.83E-05	0.408	0.593786	8	0.8	THSD7B	Extracellular Matrix
DMR2:140483001	2	140483001	140484000	1000	1	5.09E-05	0.471	0.8480543	8	0.8	LRP1B	Metabolism
DMR2:155916001	2	155916001	155917000	1000	1	4.63E-06	0.268	0.7586014	16	1.6		
DMR2:184335001	2	184335001	184336000	1000	1	5.88E-05	0.471	0.6158102	9	0.9	AC020584.1	
DMR2:191139001	2	191139001	191140000	1000	1	2.79E-05	0.435	0.6623387	8	0.8	STAT4	Transcription
DMR2:199781001	2	199781001	199782000	1000	1	7.80E-05	0.481	0.5411561	12	1.2	FTCDNL1	
DMR2:206690001	2	206690001	206692000	2000	1	1.86E-05	0.408	0.6247048	17	0.85	DYTN;VPS26CP1;Y_RNA	
DMR2:213302001	2	213302001	213303000	1000	1	7.43E-05	0.481	0.7371433	6	0.6	SPAG16	Cytoskeleton
DMR2:227967001	2	227967001	227968000	1000	1	1.39E-05	0.408	0.704799	8	0.8		
DMR2:231393001	2	231393001	231394000	1000	1	1.19E-05	0.382	-0.8122319	13	1.3	AC017104.1;B3GNT7	Golgi
DMR3:5523001	3	5523001	5524000	1000	1	6.98E-05	0.471	-0.6436052	4	0.4		
DMR3:11403001	3	11403001	11404000	1000	1	9.05E-05	0.511	0.680887	8	0.8	ATG7	Apoptosis
DMR3:12983001	3	12983001	12984000	1000	1	5.41E-05	0.471	-0.7536884	17	1.7	IQSEC1	Signaling
DMR3:28082001	3	28082001	28083000	1000	1	7.52E-05	0.481	-1.0659296	5	0.5		

DMR3:32062001	3	32062001	32063000	1000	1	4.73E-05	0.462	-1.4480294	8	0.8	OSBPL10;NIFKP7	Receptor
DMR3:34682001	3	34682001	34683000	1000	1	6.41E-05	0.471	-1.1428599	7	0.7	LINC01811;AC007483.1	
DMR3:37491001	3	37491001	37492000	1000	1	5.57E-05	0.471	-1.0293582	9	0.9	ITGA9	Extracellular Matrix
DMR3:44410001	3	44410001	44411000	1000	1	4.37E-05	0.454	-0.9945434	9	0.9	TCAIM	
DMR3:51240001	3	51240001	51242000	2000	1	1.01E-05	0.375	-0.6798456	7	0.35	DOCK3	Signaling
DMR3:52089001	3	52089001	52090000	1000	1	3.04E-05	0.438	-0.8537003	13	1.3	POC1A	Unknown
DMR3:57509001	3	57509001	57510000	1000	1	3.16E-06	0.217	0.6760021	11	1.1	DNAH12;RNU6-1181P;RNF7P1	Cytoskeleton
DMR3:57671001	3	57671001	57672000	1000	1	8.29E-05	0.494	0.4922572	20	2	DENND6A	
DMR3:60426001	3	60426001	60427000	1000	1	1.78E-05	0.408	0.5847302	8	0.8	FHIT	Signaling
DMR3:61319001	3	61319001	61320000	1000	1	1.92E-05	0.408	0.6588282	13	1.3		
DMR3:86307001	3	86307001	86308000	1000	1	6.53E-05	0.471	0.5666031	5	0.5		
DMR3:86362001	3	86362001	86363000	1000	1	7.81E-05	0.481	0.7763936	5	0.5	RN7SKP284	
DMR3:99799001	3	99799001	99800000	1000	1	3.18E-05	0.438	-1.1172438	8	0.8	COL8A1;AC069222.1	Cytoskeleton
DMR3:109136001	3	109136001	109137000	1000	1	2.18E-05	0.42	0.7837144	12	1.2	C3orf85	
DMR3:122039001	3	122039001	122040000	1000	1	3.02E-06	0.217	0.7335433	5	0.5		
DMR3:127870001	3	127870001	127871000	1000	1	9.36E-05	0.516	0.8222737	12	1.2	MGLL	Metabolism
DMR3:128712001	3	128712001	128713000	1000	1	6.12E-06	0.275	-1.0766144	10	1	RAB7A;AC079945.1	Signaling
DMR3:133945001	3	133945001	133946000	1000	1	1.75E-05	0.408	-1.3134236	21	2.1	SLCO2A1	Metabolism
DMR3:144576001	3	144576001	144577000	1000	1	2.63E-05	0.435	0.6026328	14	1.4		
DMR3:163960001	3	163960001	163961000	1000	1	8.10E-05	0.491	0.5570409	6	0.6		
DMR3:186297001	3	186297001	186299000	2000	1	5.81E-05	0.471	-0.921139	17	0.85	DGKG	Signaling
DMR3:188326001	3	188326001	188327000	1000	1	4.66E-05	0.462	0.5565019	14	1.4	LPP	Cytoskeleton
DMR3:190612001	3	190612001	190613000	1000	1	2.38E-07	0.065	0.8763578	7	0.7	IL1RAP	Receptor
DMR4:1580001	4	1580001	1582000	2000	1	6.21E-05	0.471	-0.6714989	82	4.1	AC147067.1	
DMR4:5631001	4	5631001	5632000	1000	1	2.67E-06	0.217	-1.5152429	9	0.9	EVC2	Development
DMR4:6334001	4	6334001	6335000	1000	1	6.53E-05	0.471	-1.0020287	7	0.7	PPP2R2C	Signaling
DMR4:7042001	4	7042001	7043000	1000	1	1.30E-05	0.405	-0.8274836	81	8.1	TBC1D14;AC097382.2;CCDC96;TADA2B	Signaling;Transcription
DMR4:24522001	4	24522001	24524000	2000	1	1.49E-05	0.408	0.6746577	16	0.8	DHX15;MIR573	Transcription
DMR4:24765001	4	24765001	24766000	1000	1	8.98E-05	0.509	-0.885086	6	0.6	AC006390.1;HNRNPA1P65	
DMR4:31368001	4	31368001	31369000	1000	1	3.24E-05	0.438	0.6202668	5	0.5		
DMR4:36923001	4	36923001	36925000	2000	1	6.17E-05	0.471	-1.0717595	10	0.5	AC093746.1	
DMR4:57663001	4	57663001	57664000	1000	1	6.96E-05	0.471	0.5666045	6	0.6	AC093725.2	
DMR4:59438001	4	59438001	59439000	1000	1	4.67E-05	0.462	0.9474502	7	0.7		
DMR4:59486001	4	59486001	59487000	1000	1	9.56E-05	0.518	0.6328039	9	0.9		
DMR4:66071001	4	66071001	66072000	1000	1	2.62E-05	0.435	0.6627072	11	1.1	AC116049.1	
DMR4:67501001	4	67501001	67502000	1000	1	7.05E-05	0.471	0.6496892	6	0.6	CENPC	Transcription
DMR4:83659001	4	83659001	83661000	2000	1	7.84E-05	0.481	0.5847638	17	0.85	AC021192.1	
DMR4:93002001	4	93002001	93003000	1000	1	3.35E-05	0.438	0.9874411	8	0.8	GRID2	Signaling
DMR4:94795001	4	94795001	94796000	1000	1	9.89E-05	0.52	0.6465527	10	1	BMPR1B	Receptor
DMR4:101962001	4	101962001	101963000	1000	1	6.86E-05	0.471	0.8351566	4	0.4	BANK1;MTND5P5	Development
DMR4:110971001	4	110971001	110973000	2000	1	7.66E-05	0.481	0.6730063	13	0.65		
DMR4:111097001	4	111097001	111099000	2000	1	1.67E-05	0.408	0.6054527	10	0.5		
DMR4:128558001	4	128558001	128559000	1000	1	2.72E-05	0.435	-0.9828867	8	0.8	AC110609.1;AC078850.1	
DMR4:129472001	4	129472001	129473000	1000	1	1.95E-05	0.408	-0.8073838	2	0.2		
DMR4:137769001	4	137769001	137770000	1000	1	4.38E-05	0.454	0.5498065	17	1.7		
DMR4:138763001	4	138763001	138764000	1000	1	8.73E-05	0.505	0.5415727	9	0.9	AC093766.1	
DMR4:145319001	4	145319001	145320000	1000	1	2.68E-06	0.217	0.6694378	6	0.6		
DMR4:158885001	4	158885001	158886000	1000	1	7.77E-05	0.481	0.4496857	13	1.3	FNIP2;C4orf45	
DMR4:160269001	4	160269001	160270000	1000	1	6.47E-05	0.471	0.8075447	7	0.7		
DMR5:782001	5	782001	784000	2000	1	6.19E-05	0.471	-1.1178435	70	3.5	ZDHHC11B	Unknown
DMR5:6770001	5	6770001	6771000	1000	1	3.07E-05	0.438	-0.7903896	20	2	LINC02236;AC122710.2	
DMR5:21479001	5	21479001	21490000	11000	2	9.26E-06	0.359	-0.9541424	192	1.745	GUSBP1;AC138951.1;AC138951.2	
DMR5:30971001	5	30971001	30972000	1000	1	1.56E-05	0.408	0.8207912	13	1.3		
DMR5:44526001	5	44526001	44527000	1000	1	4.26E-05	0.453	0.6276417	16	1.6	LINC02224	
DMR5:59346001	5	59346001	59347000	1000	1	5.23E-05	0.471	0.7899481	10	1	PDE4D	Metabolism
DMR5:95549001	5	95549001	95551000	2000	1	3.07E-05	0.438	0.6283731	12	0.6	TTC37;ARSK	Metabolism
DMR5:100830001	5	100830001	100831000	1000	1	5.73E-05	0.471	1.0053096	8	0.8	ST8SIA4	Golgi
DMR5:107805001	5	107805001	107806000	1000	1	7.48E-05	0.481	-0.8847111	11	1.1	RN7SKP122	
DMR5:111222001	5	111222001	111223000	1000	1	5.22E-05	0.471	-1.0003131	8	0.8	CAMK4	Signaling
DMR5:134927001	5	134927001	134929000	2000	1	1.44E-06	0.198	0.7783663	46	2.3	PCBD2;MTCYBP18;MTND6P4;MTND5P11;MTND4P12;MTND4LP30;AC008670.1;MTND3P25	Metabolism
DMR5:142980001	5	142980001	142981000	1000	1	3.66E-05	0.441	-0.8146855	9	0.9	ARHGAP26	Signaling
DMR5:156780001	5	156780001	156781000	1000	1	8.01E-05	0.487	0.5630212	15	1.5		
DMR5:165697001	5	165697001	165698000	1000	1	1.74E-05	0.408	-1.0014472	6	0.6	AC008415.1	
DMR5:166922001	5	166922001	166923000	1000	1	1.96E-05	0.408	0.7232912	12	1.2	LINC01947	
DMR5:170186001	5	170186001	170188000	2000	1	9.92E-05	0.52	-1.2103188	14	0.7	LINC01187	
DMR6:1625001	6	1625001	1626000	1000	1	2.27E-05	0.425	-0.6489596	56	5.6	GMDS	Metabolism
DMR6:3551001	6	3551001	3552000	1000	1	5.21E-05	0.471	-0.7166055	4	0.4		
DMR6:22521001	6	22521001	22522000	1000	1	3.25E-05	0.438	-0.7116362	11	1.1	CASC15	

DMR6:39310001	6	39310001	39311000	1000	1	1.86E-05	0.408	-0.9947138	13	1.3	KCNK17;KCNK16	Transport
DMR6:66218001	6	66218001	66219000	1000	1	7.69E-05	0.481	1.1180111	5	0.5		
DMR6:70556001	6	70556001	70557000	1000	1	7.49E-05	0.481	0.5447932	6	0.6	FAM135A;SDHAF4	Unknown
DMR6:76910001	6	76910001	76912000	2000	1	5.45E-07	0.112	1.0181472	19	0.95	AL355612.1	
DMR6:80557001	6	80557001	80558000	1000	1	5.75E-06	0.275	0.6193325	9	0.9	AL590824.1	
DMR6:81063001	6	81063001	81064000	1000	1	5.18E-06	0.268	-0.9791678	11	1.1		
DMR6:82600001	6	82600001	82602000	2000	1	3.07E-05	0.438	0.7386983	9	0.45		
DMR6:86612001	6	86612001	86613000	1000	1	4.89E-05	0.467	0.7019935	13	1.3		
DMR6:94043001	6	94043001	94044000	1000	1	2.08E-05	0.409	0.5663227	4	0.4		
DMR6:112046001	6	112046001	112047000	1000	1	1.13E-05	0.382	-0.8400109	4	0.4	CCN6	
DMR6:114285001	6	114285001	114286000	1000	1	4.06E-05	0.453	0.8257192	7	0.7	HDAC2-AS2;HS3ST5	Metabolism
DMR6:117377001	6	117377001	117378000	1000	1	5.20E-05	0.471	0.7073228	15	1.5	ROS1;AL132671.2	Receptor
DMR6:122576001	6	122576001	122577000	1000	1	3.89E-05	0.451	0.7710695	20	2	PKIB;AL512283.2	Signaling
DMR6:123495001	6	123495001	123496000	1000	1	5.27E-05	0.471	0.6173573	9	0.9	TRDN;TRDN-AS1	Cytoskeleton
DMR6:133912001	6	133912001	133913000	1000	1	6.33E-05	0.471	0.6123218	5	0.5		
DMR6:162449001	6	162449001	162452000	3000	1	7.89E-05	0.482	0.7108695	33	1.1	PRKN	
DMR7:12335001	7	12335001	12336000	1000	1	8.66E-05	0.504	0.6623318	16	1.6	VWDE	
DMR7:15421001	7	15421001	15422000	1000	1	2.17E-06	0.213	0.6222877	10	1	AGMO	Metabolism
DMR7:21352001	7	21352001	21353000	1000	1	6.81E-05	0.471	0.5174112	7	0.7		
DMR7:35976001	7	35976001	35977000	1000	1	1.27E-06	0.188	0.6894546	12	1.2	SEPTIN7P3;PPP1R14BP4;AC083864.5	
DMR7:43810001	7	43810001	43811000	1000	1	8.95E-05	0.509	-0.6821442	19	1.9	BLVRA	Metabolism
DMR7:46857001	7	46857001	46858000	1000	1	3.54E-05	0.438	0.7632575	6	0.6		
DMR7:82799001	7	82799001	82801000	2000	1	5.65E-05	0.471	0.6143431	23	1.15	PCLO	Extracellular Matrix
DMR7:101802001	7	101802001	101803000	1000	1	7.43E-05	0.481	-0.6535858	10	1		
DMR7:132059001	7	132059001	132060000	1000	1	5.36E-05	0.471	-0.621148	12	1.2		
DMR7:139261001	7	139261001	139263000	2000	1	1.15E-05	0.382	0.5752809	29	1.45	UBN2	Transcription
DMR7:142515001	7	142515001	142516000	1000	1	6.78E-05	0.471	-1.0078572	5	0.5	TRBV6-8;TRBV7-7;TRBV5-7	
DMR7:146179001	7	146179001	146180000	1000	1	2.65E-05	0.435	0.7038097	9	0.9	CNTNAP2	Receptor
DMR7:149644001	7	149644001	149645000	1000	1	5.71E-05	0.471	-1.035568	9	0.9		
DMR7:155215001	7	155215001	155216000	1000	1	6.24E-05	0.471	-0.749398	16	1.6	AC099552.3;AC099552.5;AC099552.4	
DMR8:2890001	8	2890001	2891000	1000	1	8.86E-05	0.508	0.5852425	12	1.2		
DMR8:6989001	8	6989001	6995000	6000	1	4.62E-05	0.462	-0.7672174	84	1.4	DEFA1;DEFT1P;DEFA1B	
DMR8:27234001	8	27234001	27235000	1000	1	3.35E-05	0.438	-1.2454963	4	0.4	STMN4	Unknown
DMR8:30746001	8	30746001	30747000	1000	1	3.02E-05	0.438	-0.7059706	19	1.9	UBXN8;HIKESHIP3	Proteolysis
DMR8:35666001	8	35666001	35667000	1000	1	6.27E-05	0.471	0.7734065	8	0.8	UNC5D;AC105230.1	Receptor
DMR8:57030001	8	57030001	57031000	1000	1	2.63E-05	0.435	0.7230083	14	1.4		
DMR8:67214001	8	67214001	67215000	1000	1	3.57E-05	0.438	0.655981	6	0.6	ARFGEF1	Translation
DMR8:72538001	8	72538001	72540000	2000	1	3.62E-05	0.438	0.5989366	34	1.7	KCNB2	Metabolism
DMR8:77947001	8	77947001	77948000	1000	1	6.95E-06	0.296	0.9714593	4	0.4		
DMR8:80463001	8	80463001	80464000	1000	1	7.39E-05	0.481	-0.7119067	8	0.8	AC034114.2	
DMR8:92550001	8	92550001	92551000	1000	1	6.15E-05	0.471	0.5185465	7	0.7	AC091096.1	
DMR8:92905001	8	92905001	92907000	2000	1	3.59E-05	0.438	0.7446007	20	1	AC117834.1;TRIQK	
DMR8:114203001	8	114203001	114204000	1000	1	5.63E-05	0.471	0.5449991	20	2		
DMR8:117407001	8	117407001	117408000	1000	1	9.40E-05	0.516	-0.7412776	5	0.5		
DMR8:138858001	8	138858001	138859000	1000	1	5.93E-06	0.275	-0.6900014	13	1.3	COL22A1	Extracellular Matrix
DMR8:143420001	8	143420001	143422000	2000	1	6.75E-05	0.471	-0.7040322	108	5.4	AC105118.1;MAFA-AS1;MAFA	
DMR9:27725001	9	27725001	27726000	1000	1	6.07E-07	0.112	-1.0239234	11	1.1		
DMR9:38570001	9	38570001	38571000	1000	1	7.10E-05	0.471	0.6417558	10	1	FAM95C;AL390726.2;SNX18P3;ANKRD18A	
DMR9:70323001	9	70323001	70324000	1000	1	6.85E-05	0.471	0.5787776	8	0.8	SMC5	Transcription
DMR9:75709001	9	75709001	75711000	2000	1	3.55E-05	0.438	0.7029119	8	0.4		
DMR9:89688001	9	89688001	89689000	1000	1	9.26E-05	0.516	-0.8734459	12	1.2	UNQ6494	
DMR9:95625001	9	95625001	95627000	2000	1	6.89E-05	0.471	-0.7548488	42	2.1		
DMR9:96524001	9	96524001	96525000	1000	1	9.82E-05	0.52	-0.8974444	9	0.9	CDC14B	Signaling
DMR9:101899001	9	101899001	101900000	1000	1	1.84E-05	0.408	0.6613652	11	1.1		
DMR9:114422001	9	114422001	114423000	1000	1	2.21E-06	0.213	-0.799119	10	1	WHRN	
DMR9:115038001	9	115038001	115040000	2000	1	3.49E-05	0.438	-0.8506037	16	0.8	DELEC1;TNC	Extracellular Matrix
DMR9:121295001	9	121295001	121296000	1000	1	3.05E-06	0.217	-0.6742144	15	1.5	GSN;GSN-AS1	Cytoskeleton
DMR9:129384001	9	129384001	129385000	1000	1	5.24E-06	0.268	-0.9200623	25	2.5	AL353803.3	
DMR9:130432001	9	130432001	130433000	1000	1	2.23E-05	0.425	-0.6671665	29	2.9	HMCN2	
DMR9:130832001	9	130832001	130833000	1000	1	6.49E-05	0.471	-0.6208615	8	0.8	ABL1	Signaling
DMR10:7189001	10	7189001	7190000	1000	1	9.65E-05	0.518	0.6789917	14	1.4	SFMBT2	Transcription
DMR10:16565001	10	16565001	16566000	1000	1	8.86E-05	0.508	0.723885	10	1		
DMR10:19497001	10	19497001	19498000	1000	1	4.67E-05	0.462	0.8458093	6	0.6	MALRD1;HMGN1P20;AL354695.1	
DMR10:40704001	10	40704001	40705000	1000	1	9.41E-05	0.516	0.6708838	17	1.7		
DMR10:50535001	10	50535001	50536000	1000	1	5.30E-06	0.268	-1.0232828	13	1.3	SGMS1	Metabolism
DMR10:53019001	10	53019001	53020000	1000	1	7.80E-05	0.481	0.6855871	18	1.8	LINC02672	
DMR10:54270001	10	54270001	54271000	1000	1	4.20E-05	0.453	0.9875359	4	0.4	PCDH15	Extracellular Matrix
DMR10:68799001	10	68799001	68802000	3000	1	4.86E-08	0.025	-1.2821219	30	1	CCAR1	Signaling
DMR10:69481001	10	69481001	69482000	1000	1	2.26E-05	0.425	-1.1788613	9	0.9	TSPAN15	Extracellular Matrix
DMR10:92612001	10	92612001	92614000	2000	1	9.55E-05	0.518	-1.0400748	15	0.75	KIF11	Cytoskeleton

DMR10:111097001	10	111097001	111100000	3000	1	6.63E-05	0.471	-0.8121692	28	0.933	AL355863.1	
DMR10:112168001	10	112168001	112169000	1000	1	4.00E-05	0.451	0.6393579	12	1.2	GPAM	Metabolism
DMR10:125333001	10	125333001	125334000	1000	1	3.98E-05	0.451	-1.266698	8	0.8		
DMR11:8849001	11	8849001	8850000	1000	1	2.49E-05	0.434	-0.6955843	11	1.1	DENND2B;RNA5SP330	
DMR11:15892001	11	15892001	15894000	2000	1	8.14E-05	0.491	-0.6837338	23	1.15	AC009869.1	
DMR11:21719001	11	21719001	21720000	1000	1	3.93E-05	0.451	-0.9598133	8	0.8		
DMR11:44998001	11	44998001	4.50E+07	2000	1	4.49E-05	0.455	-0.8476968	15	0.75	AC068858.1	
DMR11:52089001	11	52089001	52090000	1000	1	6.15E-05	0.471	-1.0905006	13	1.3		
DMR11:53757001	11	53757001	53758000	1000	1	4.93E-08	0.025	-1.8858114	11	1.1		
DMR11:54240001	11	54240001	54241000	1000	1	4.26E-05	0.453	-0.9743233	10	1		
DMR11:63971001	11	63971001	63972000	1000	1	7.57E-05	0.481	-1.1133481	10	1	RNU6-45P;COX8A;AP000721.1	Electron Transport
DMR11:76544001	11	76544001	76545000	1000	1	3.30E-05	0.438	0.5486525	18	1.8	EMSY	
DMR11:85450001	11	85450001	85451000	1000	1	5.38E-05	0.471	0.5239282	7	0.7	DLG2;AP003035.1	
DMR11:90820001	11	90820001	90821000	1000	1	5.03E-07	0.112	0.8899287	10	1	DISC1FP1	
DMR11:98637001	11	98637001	98638000	1000	1	1.25E-05	0.396	0.7033013	9	0.9		
DMR11:107432001	11	107432001	107433000	1000	1	1.96E-05	0.408	0.5864926	11	1.1	CWF19L2	
DMR11:111407001	11	111407001	111408000	1000	1	9.33E-06	0.359	-0.9127118	11	1.1	POU2AF1;AP002008.3	
DMR11:116077001	11	116077001	116078000	1000	1	7.60E-05	0.481	-0.9025341	9	0.9		
DMR11:122554001	11	122554001	122556000	2000	1	3.52E-05	0.438	0.5122748	26	1.3	MIR100HG	
DMR11:124081001	11	124081001	124082000	1000	1	3.52E-06	0.226	0.7123803	10	1		
DMR11:126513001	11	126513001	126514000	1000	1	5.81E-05	0.471	-0.6300415	13	1.3	KIRREL3	Extracellular Matrix
DMR11:128652001	11	128652001	128653000	1000	1	6.34E-05	0.471	0.6797855	7	0.7	AP001122.1	
DMR12:6075001	12	6075001	6077000	2000	1	9.62E-05	0.518	-0.7555824	53	2.65	VWF	Extracellular Matrix
DMR12:15625001	12	15625001	15626000	1000	1	4.90E-05	0.467	0.6089829	5	0.5	EPS8	Signaling
DMR12:20606001	12	20606001	20607000	1000	1	6.48E-05	0.471	0.6803373	9	0.9	PDE3A	Signaling
DMR12:22249001	12	22249001	22250000	1000	1	7.61E-05	0.481	0.6084745	14	1.4	ST8SIA1	Metabolism
DMR12:31234001	12	31234001	31235000	1000	1	8.71E-05	0.505	-0.7667061	14	1.4	AC024940.4	
DMR12:44745001	12	44745001	44748000	3000	1	1.84E-05	0.408	0.7592098	30	1	NELL2	Signaling
DMR12:51343001	12	51343001	51345000	2000	1	2.50E-05	0.434	-0.5924982	22	1.1	CELA1;GALNT6	Protease;Metabolism
DMR12:55707001	12	55707001	55710000	3000	1	9.10E-05	0.513	-0.5694239	86	2.867	ITGA7;AC009779.3;BLOC1S1	Signaling;Metabolism
DMR12:66401001	12	66401001	66403000	2000	1	3.32E-05	0.438	-0.9040798	17	0.85	GRIP1	Signaling
DMR12:76490001	12	76490001	76491000	1000	1	3.80E-05	0.449	0.613741	17	1.7	OSBPL8	Receptor
DMR12:82189001	12	82189001	82190000	1000	1	6.12E-05	0.471	0.7666015	2	0.2		
DMR12:83732001	12	83732001	83733000	1000	1	2.59E-05	0.435	0.8586968	5	0.5		
DMR12:104010001	12	104010001	104011000	1000	1	2.48E-05	0.434	-0.9331038	6	0.6	GLT8D2	
DMR12:113078001	12	113078001	113082000	4000	1	6.12E-05	0.471	-1.0367816	61	1.525	DTX1	Transcription
DMR12:125735001	12	125735001	125736000	1000	1	4.69E-05	0.462	-0.8764184	5	0.5		
DMR12:128183001	12	128183001	128184000	1000	1	2.97E-05	0.438	-0.8519354	6	0.6	AC061709.2	
DMR12:129213001	12	129213001	129214000	1000	1	8.41E-05	0.497	-0.8553562	12	1.2	TMEM132D;TMEM132D-AS2	Unknown
DMR12:131072001	12	131072001	131073000	1000	1	3.60E-05	0.438	-0.80965	14	1.4	ADGRD1	
DMR13:22667001	13	22667001	22668000	1000	1	2.05E-05	0.409	0.8438494	10	1		
DMR13:24163001	13	24163001	24164000	1000	1	6.41E-07	0.112	-1.2725998	6	0.6	AL359736.1;SPATA13;MIR2276	Development
DMR13:44860001	13	44860001	44861000	1000	1	8.75E-05	0.505	-1.0220794	6	0.6		
DMR13:53787001	13	53787001	53788000	1000	1	6.89E-06	0.296	1.0167041	7	0.7	AL356295.1	
DMR13:55442001	13	55442001	55443000	1000	1	4.47E-05	0.455	-1.1103655	2	0.2		
DMR13:58903001	13	58903001	58904000	1000	1	9.14E-05	0.514	0.8281087	7	0.7		
DMR13:69855001	13	69855001	69856000	1000	1	6.88E-05	0.471	0.7924798	13	1.3	KLHL1	Cytoskeleton
DMR13:88217001	13	88217001	88218000	1000	1	1.80E-05	0.408	0.8489172	11	1.1	LINC00373	
DMR13:103330001	13	103330001	103331000	1000	1	6.58E-05	0.471	0.6194602	10	1		
DMR13:113066001	13	113066001	113067000	1000	1	1.82E-05	0.408	-0.8283226	32	3.2	MCF2L	Signaling
DMR14:46624001	14	46624001	46625000	1000	1	3.79E-05	0.449	0.7786101	4	0.4		
DMR14:54785001	14	54785001	54786000	1000	1	5.33E-06	0.268	-1.2740484	10	1	SAMD4A	Signaling
DMR14:57274001	14	57274001	57275000	1000	1	5.95E-05	0.471	0.7816553	11	1.1	EXOC5;AP5M1	Metabolism
DMR14:76378001	14	76378001	76379000	1000	1	3.05E-05	0.438	-1.0160978	11	1.1	ESRRB	
DMR14:77015001	14	77015001	77017000	2000	1	7.57E-05	0.481	-0.641032	28	1.4	IRF2BPL	
DMR14:93840001	14	93840001	93841000	1000	1	8.45E-05	0.498	-0.7018878	11	1.1		
DMR14:97822001	14	97822001	97823000	1000	1	3.55E-05	0.438	-0.8615257	4	0.4		
DMR14:100325001	14	100325001	100327000	2000	1	2.72E-05	0.435	-0.6421366	56	2.8	RN7SL523P;SLC25A47;AL157871.4;WARS1	
DMR14:102506001	14	102506001	102507000	1000	1	7.10E-05	0.471	-0.575613	28	2.8	TECPR2;RNU6-244P;ANKRD9	Unknown
DMR14:103963001	14	103963001	103964000	1000	1	9.29E-05	0.516	0.658539	10	1	TDRD9	Transcription
DMR14:104801001	14	104801001	104803000	2000	1	7.83E-07	0.126	-1.3163074	127	6.35	AKT1;ZBTB42	Signaling
DMR15:18359001	15	18359001	18363000	4000	1	3.86E-05	0.451	1.1195415	64	1.6		
DMR15:18780001	15	18780001	18782000	2000	1	5.26E-08	0.025	2.3784582	29	1.45		
DMR15:18800001	15	18800001	18804000	4000	1	2.19E-07	0.065	1.8128625	57	1.425		
DMR15:19311001	15	19311001	19312000	1000	1	1.57E-06	0.202	1.162665	16	1.6		
DMR15:19594001	15	19594001	19595000	1000	1	2.36E-07	0.065	1.5224822	14	1.4		
DMR15:19676001	15	19676001	19677000	1000	1	1.19E-05	0.382	1.1379775	15	1.5		
DMR15:19685001	15	19685001	19686000	1000	1	9.58E-09	0.018	2.4903836	15	1.5		

DMR15:25203001	15	25203001	25204000	1000	1	4.32E-05	0.454	-0.7669075	18	1.8	SNHG14;SNORD115-13;SNORD115-14;SNORD115-15;SNORD115-16;SNORD115-17;SNORD115-18;SNORD115-19;SNORD115-20;SNORD115-21;SNORD115-22;SNORD115-23;SNORD115-24	
DMR15:26014001	15	26014001	26015000	1000	1	6.52E-05	0.471	0.7875142	14	1.4	LINC02346;AC044913.1	
DMR15:35684001	15	35684001	35685000	1000	1	1.65E-05	0.408	0.7375264	7	0.7	DPH6-DT	
DMR15:43567001	15	43567001	43568000	1000	1	7.52E-06	0.309	-0.6897978	6	0.6	PPIP5K1	Signaling
DMR15:54053001	15	54053001	54054000	1000	1	4.21E-05	0.453	0.5616704	9	0.9	UNC13C;AC010867.1	Development
DMR15:68987001	15	68987001	68988000	1000	1	9.27E-05	0.516	-0.9217937	10	1	AC027088.1;NOX5;AC027088.5	Metabolism
DMR15:73728001	15	73728001	73729000	1000	1	4.87E-06	0.268	-0.7976065	10	1	AC022188.1;INSYN1	
DMR15:77605001	15	77605001	77606000	1000	1	8.38E-05	0.497	-0.7321783	11	1.1	AC046168.2;LINGO1	Development
DMR15:80723001	15	80723001	80724000	1000	1	4.75E-05	0.462	-0.8615766	13	1.3	ABHD17C	
DMR15:92108001	15	92108001	92109000	1000	1	4.10E-05	0.453	-0.8116343	13	1.3	SLCO3A1	Metabolism
DMR15:93040001	15	93040001	93043000	3000	1	9.43E-05	0.516	-1.0044582	67	2.233	RGMA	
DMR16:1327001	16	1327001	1328000	1000	1	2.91E-06	0.217	-0.9024217	23	2.3	UBE2I;AL031714.1;RPS20P2;BAIAP3	Metabolism
DMR16:3291001	16	3291001	3292000	1000	1	7.90E-05	0.482	-0.8045803	6	0.6	ZNF263;AC004232.2;TIGD7	Transcription
DMR16:11503001	16	11503001	11504000	1000	1	7.05E-06	0.296	-0.9237646	17	1.7	AC099489.1	
DMR16:28477001	16	28477001	28478000	1000	1	7.71E-05	0.481	-0.6090094	19	1.9	AC009093.11;AC138894.1;NPIPB7;CLN3	Transport
DMR16:29245001	16	29245001	29248000	3000	1	6.96E-05	0.471	0.4923802	38	1.267	AC009093.11;AC009093.10	
DMR16:55427001	16	55427001	55428000	1000	1	3.98E-05	0.451	-1.3327436	8	0.8	MMP2;AC007336.2;MMP2-AS1	Proteolysis
DMR16:57362001	16	57362001	57363000	1000	1	3.83E-06	0.238	-0.8756713	9	0.9	CCL22;CX3CL1	Signaling;Growth Factors & Cytokines
DMR16:63948001	16	63948001	63950000	2000	1	6.94E-05	0.471	0.5994341	23	1.15		
DMR16:66192001	16	66192001	66194000	2000	1	5.51E-05	0.471	-0.9891279	10	0.5		
DMR16:70643001	16	70643001	70644000	1000	1	1.93E-05	0.408	-0.6383482	14	1.4	IL34	Growth Factors & Cytokines
DMR16:76935001	16	76935001	76936000	1000	1	2.12E-06	0.213	0.6433537	11	1.1	AC106729.1	
DMR16:85109001	16	85109001	85110000	1000	1	3.32E-05	0.438	-0.9260587	12	1.2	CIBAR2	
DMR16:86080001	16	86080001	86081000	1000	1	6.95E-05	0.471	-0.7466166	18	1.8	AC135012.1	
DMR17:21860001	17	21860001	21992000	132000	1	9.34E-05	0.516	0.501216	3294	2.495		
DMR17:27779001	17	27779001	27780000	1000	1	1.32E-05	0.405	-0.6788586	7	0.7	NOS2	Metabolism
DMR17:36492001	17	36492001	36493000	1000	1	2.75E-05	0.435	-0.7213816	10	1	ZNHIT3;RNA5SP439;MYO19	Transcription;Cytoskeleton
DMR17:40478001	17	40478001	40480000	2000	1	8.16E-05	0.491	-0.5770026	43	2.15	AC018629.1;TNS4	Signaling
DMR17:42318001	17	42318001	42319000	1000	1	7.38E-05	0.481	-0.651074	9	0.9	STAT5A;STAT3	Transcription
DMR17:43844001	17	43844001	43846000	2000	1	7.04E-05	0.471	-0.7765098	21	1.05	CD300LG	Receptor
DMR17:59048001	17	59048001	59049000	1000	1	8.94E-05	0.509	0.5410918	12	1.2	TRIM37	Development
DMR17:69788001	17	69788001	69789000	1000	1	6.07E-05	0.471	0.7674975	10	1	LINC01483	
DMR17:74874001	17	74874001	74875000	1000	1	1.70E-05	0.408	-0.7393673	9	0.9	FDXR;FADS6	Metabolism
DMR17:77528001	17	77528001	77530000	2000	1	1.90E-05	0.408	-0.8465303	98	4.9	AC021683.3	
DMR18:750001	18	750001	752000	2000	1	3.13E-05	0.438	0.6611984	10	0.5	YES1	Transcription
DMR18:13112001	18	13112001	13113000	1000	1	4.77E-05	0.462	-0.9966081	4	0.4	CEP192	
DMR18:13861001	18	13861001	13862000	1000	1	2.17E-05	0.42	-1.0792433	4	0.4		
DMR18:23745001	18	23745001	23746000	1000	1	3.86E-05	0.451	0.7458169	3	0.3	LAMA3	Extracellular Matrix
DMR18:24440001	18	24440001	24441000	1000	1	6.15E-06	0.275	0.6290614	8	0.8	IMPACT;AC007922.2	
DMR18:37521001	18	37521001	37522000	1000	1	5.87E-05	0.471	-0.6068698	22	2.2	CELF4	Transcription
DMR18:39961001	18	39961001	39962000	1000	1	7.03E-05	0.471	0.7846533	8	0.8		
DMR18:46343001	18	46343001	46344000	1000	1	2.39E-05	0.434	-1.2579993	14	1.4	RNF165	Development
DMR18:51237001	18	51237001	51238000	1000	1	3.43E-05	0.438	-0.829986	3	0.3		
DMR18:61984001	18	61984001	61985000	1000	1	2.41E-05	0.434	0.8060535	6	0.6	PIGN	Signaling
DMR18:78176001	18	78176001	78178000	2000	1	5.54E-05	0.471	0.5493805	24	1.2		
DMR19:4583001	19	4583001	4584000	1000	1	1.96E-05	0.408	-0.7967856	21	2.1	SEMA6B	Signaling
DMR19:33677001	19	33677001	33679000	2000	1	6.95E-05	0.471	-0.8218744	15	0.75	CHST8	Metabolism
DMR19:37295001	19	37295001	37304000	9000	1	5.93E-05	0.471	-0.6724608	609	6.767	AC016590.1;AC016590.4;ZNF875	
DMR19:46853001	19	46853001	46854000	1000	1	6.39E-05	0.471	-0.6680804	11	1.1	AP2S1;ARHGAP35	Transport;Signaling
DMR19:50575001	19	50575001	50578000	3000	1	2.89E-05	0.438	-0.7077369	41	1.367	LRRC4B	Extracellular Matrix
DMR19:52957001	19	52957001	52958000	1000	1	5.31E-05	0.471	0.8193362	19	1.9	ZNF816-ZNF321P;ZNF816;AC010328.3;ZNF702P	
DMR19:56658001	19	56658001	56659000	1000	1	2.67E-05	0.435	-0.7184137	7	0.7	ZIM2-AS1;SMIM17;ZNF835	Transcription
DMR20:23268001	20	23268001	23270000	2000	1	9.81E-05	0.52	-0.7430207	17	0.85		
DMR20:37900001	20	37900001	37901000	1000	1	2.46E-05	0.434	-0.6555162	12	1.2	VSTM2L	Unknown
DMR20:40926001	20	40926001	40927000	1000	1	2.72E-05	0.435	0.6951641	12	1.2		
DMR20:41256001	20	41256001	41258000	2000	1	6.79E-05	0.471	0.6864399	16	0.8	ZHX3	Transcription
DMR20:48224001	20	48224001	48225000	1000	1	3.95E-06	0.238	-0.9122721	15	1.5		
DMR20:53417001	20	53417001	53418000	1000	1	9.84E-05	0.52	-0.7762736	7	0.7	TSHZ2;AL109930.1;AL354993.1;PPIAP10	Transcription

DMR21:21041001	21	21041001	21042000	1000	1	8.39E-05	0.497	0.6059046	6	0.6	NCAM2	Cytoskeleton
DMR21:22814001	21	22814001	22817000	3000	1	1.96E-05	0.408	0.9157695	46	1.533		
DMR21:36187001	21	36187001	36188000	1000	1	2.07E-05	0.409	-0.8920917	12	1.2	DOP1B	
DMR21:36829001	21	36829001	36830000	1000	1	1.08E-05	0.378	-0.8195215	10	1	HLCS	Metabolism
DMR21:38953001	21	38953001	38954000	1000	1	9.49E-06	0.359	-0.817977	9	0.9	AP001042.1	
DMR22:14340001	22	14340001	14341000	1000	1	3.44E-05	0.438	-1.0401586	18	1.8		
DMR22:18013001	22	18013001	18014000	1000	1	5.59E-05	0.471	-1.0723413	5	0.5	MICAL3;AC016027.2;RHEBP3	
DMR22:21161001	22	21161001	21164000	3000	1	6.22E-05	0.471	0.7143196	33	1.1	FAM230B	
DMR22:22305001	22	22305001	22307000	2000	1	1.00E-04	0.52	0.4594209	40	2	AC245060.7;AC245060.4	
DMR22:28017001	22	28017001	28018000	1000	1	4.28E-05	0.453	-0.716362	14	1.4	TTC28-AS1;TTC28	Unknown
DMR22:30546001	22	30546001	30548000	2000	1	4.19E-05	0.453	-0.511316	28	1.4	SEC14L6;SIRPAP1;GAL3ST1	Metabolism
DMR22:30626001	22	30626001	30627000	1000	1	4.72E-05	0.462	-0.9742771	16	1.6	TCN2;SLC35E4	Transport
DMR22:38879001	22	38879001	38880000	1000	1	2.32E-05	0.425	-1.086084	8	0.8	CBX6;AL022318.5	Transcription
DMR22:42226001	22	42226001	42227000	1000	1	1.07E-05	0.378	-0.7721307	9	0.9	TCF20	Transcription
DMR22:44647001	22	44647001	44650000	3000	1	1.73E-06	0.208	-0.7517442	52	1.733	ANP32BP2	
DMR22:44726001	22	44726001	44727000	1000	1	1.47E-05	0.408	-0.7312559	26	2.6	PRR5;PRR5-ARHGAP8	Signaling
DMRX:11630001	X	11630001	11631000	1000	1	7.12E-05	0.471	-0.7752028	9	0.9	ARHGAP6	Cytoskeleton
DMRX:14629001	X	14629001	14630000	1000	1	6.64E-05	0.471	-0.7685527	5	0.5	GLRA2	Receptor
DMRX:17247001	X	17247001	17248000	1000	1	2.01E-05	0.409	-0.7545059	6	0.6		
DMRX:23308001	X	23308001	23309000	1000	1	6.44E-05	0.471	-0.7124221	10	1		
DMRX:34503001	X	34503001	34504000	1000	1	7.18E-05	0.474	0.5969024	7	0.7		
DMRX:34978001	X	34978001	34979000	1000	1	4.82E-05	0.465	0.7886645	10	1		
DMRX:54926001	X	54926001	54927000	1000	1	8.55E-05	0.501	-0.9422005	9	0.9	TRO;SNORA11G;PFKFB1	Extracellular Matrix
DMRX:63986001	X	63986001	63987000	1000	1	2.80E-05	0.435	0.6433322	9	0.9		
DMRX:67699001	X	67699001	67700000	1000	1	1.68E-05	0.408	-1.085977	10	1	AR	Epigenetic
DMRX:77668001	X	77668001	77669000	1000	1	5.66E-05	0.471	0.6951908	12	1.2	ATRX	Epigenetic
DMRX:103922001	X	103922001	103923000	1000	1	9.56E-05	0.518	-0.6454528	11	1.1	AC234782.2;TMSB15B-AS1;TMSB15B;SLC25A53P1	
DMRX:105305001	X	105305001	105306000	1000	1	8.25E-05	0.493	0.5755639	7	0.7	IL1RAPL2	Receptor
DMRX:111658001	X	111658001	111659000	1000	1	6.68E-05	0.471	0.6419992	7	0.7	ALG13	
DMRX:113186001	X	113186001	113187000	1000	1	7.64E-05	0.481	-0.9236849	3	0.3	AC002072.1;AL023877.1	
DMRX:113369001	X	113369001	113371000	2000	1	8.04E-06	0.323	0.6617598	27	1.35		
DMRX:118820001	X	118820001	118821000	1000	1	8.63E-05	0.504	-1.1784297	5	0.5	ZCCHC12	Transcription
DMRX:154412001	X	154412001	154414000	2000	1	9.33E-05	0.516	-0.7254908	44	2.2	RPL10;DNASE1L1;TAZ	Metabolism
DMRY:11728001	Y	11728001	11750000	22000	1	5.10E-05	0.471	0.614367	527	2.395		
