# Supplementary material for: Epigenome association study for DNA methylation biomarkers in buccal and monocyte cells for female rheumatoid arthritis
Source: Sci Rep. 2021 Dec 10;11:23789. doi: 10.1038/s41598-021-03170-6 (PMC8664902; doi:10.1038/s41598-021-03170-6)
Supplement: Supplementary file 6 — Supplementary Table S4. [file 41598_2021_3170_MOESM6_ESM.pdf]

Supplemental Table S4
Combined AA & CC DMR Table 1e-04

DMR Name	Chr	Start	Stop	Length	# Sig Win	minP	Min FDR	maxLFC	CpG #	CpG Density	Gene Annotation	Gene Category
DMR1:3598001	1	3598001	3599000	1000	1	2.93E-05	0.424	-0.8804175	48	4.8	MEGF6	Growth Factors & Cytokines
DMR1:6636001	1	6636001	6638000	2000	1	9.40E-06	0.317	-0.6997226	43	2.15	THAP3;DNAJC11	Transcription;Protein Binding
DMR1:7501001	1	7501001	7503000	2000	1	3.17E-05	0.424	-0.6906492	44	2.2	CAMTA1	Transcription
DMR1:15414001	1	15414001	15415000	1000	1	8.78E-05	0.49	-0.5890549	20	2	;EFHD2	Signaling
DMR1:21904001	1	21904001	21905000	1000	1	4.60E-05	0.44	-0.5862805	32	3.2	HSPG2;	Extracellular Matrix
DMR1:24315001	1	24315001	24317000	2000	1	7.76E-05	0.489	-0.6694155	22	1.1	GRHL3-AS1;GRHL3	Transcription
DMR1:25211001	1	25211001	25212000	1000	1	3.33E-06	0.252	-0.661967	7	0.7	NA	
DMR1:45719001	1	45719001	45720000	1000	1	6.89E-05	0.468	0.406369	17	1.7	IPP	Cytoskeleton
DMR1:52362001	1	52362001	52363000	1000	1	7.96E-05	0.49	-0.5770178	8	0.8	CC2D1B;;PLA2G12AP1;ORC1	
DMR1:59215001	1	59215001	59216000	1000	1	2.75E-05	0.418	-0.6993767	10	1	LINC01358;FGGY-DT	
DMR1:80644001	1	80644001	80645000	1000	1	1.34E-05	0.33	0.3837008	8	0.8	LINC01781	
DMR1:83225001	1	83225001	83226000	1000	1	2.37E-05	0.395	0.4798846	4	0.4		
DMR1:88928001	1	88928001	88930000	2000	1	8.84E-05	0.49	-0.5313502	30	1.5	;KYAT3	
DMR1:110116001	1	110116001	110118000	2000	1	4.77E-06	0.252	-0.6638721	22	1.1	LINC01397;UBL4B	Transcription
DMR1:115711001	1	115711001	115712000	1000	1	6.51E-05	0.466	-0.471627	14	1.4	CASQ2	Signaling
DMR1:119194001	1	119194001	119195000	1000	1	5.90E-05	0.465	0.4564962	7	0.7	WARS2-AS1	
DMR1:156680001	1	156680001	156682000	2000	1	9.36E-05	0.49	-0.445302	23	1.15	NES;	Cytoskeleton
DMR1:157639001	1	157639001	157640000	1000	1	3.69E-05	0.427	-0.5616991	12	1.2	NA	
DMR1:161082001	1	161082001	161083000	1000	1	6.43E-05	0.466	-0.6959236	7	0.7	NECTIN4;NECTIN4-AS1	
DMR1:173455001	1	173455001	173456000	1000	1	5.63E-05	0.463	0.3926978	8	0.8	PRDX6-AS1	
DMR1:184321001	1	184321001	184323000	2000	1	8.46E-05	0.49	0.4209902	20	1	Y_RNA;	
DMR1:205932001	1	205932001	205933000	1000	1	6.15E-05	0.466	-0.5640275	10	1	SLC26A9;	Transport
DMR1:223415001	1	223415001	223417000	2000	1	4.26E-05	0.434	0.5701862	13	0.65		
DMR1:226987001	1	226987001	226988000	1000	1	8.18E-05	0.49	-0.5531006	22	2.2	;COQ8A;CDC42BPA	Signaling
DMR1:227732001	1	227732001	227733000	1000	1	1.24E-07	0.096	-0.8124598	30	3	SNAP47;JMJD4	Cytoskeleton;Receptor
DMR1:234105001	1	234105001	234106000	1000	1	8.37E-05	0.49	0.4749841	12	1.2	SLC35F3	
DMR1:237735001	1	237735001	237737000	2000	1	9.33E-05	0.49	-0.5430518	15	0.75	RYR2	Receptor
DMR2:4197001	2	4197001	4198000	1000	1	4.80E-05	0.443	0.4415755	6	0.6		
DMR2:10191001	2	10191001	10192000	1000	1	1.03E-05	0.323	-0.5253227	22	2.2	RRM2;MIR4261	
DMR2:11801001	2	11801001	11804000	3000	1	3.84E-05	0.432	-0.7931942	31	1.033	LPIN1	Development
DMR2:18240001	2	18240001	18241000	1000	1	8.63E-05	0.49	-0.529467	11	1.1	KCNS3	
DMR2:25961001	2	25961001	25962000	1000	1	4.05E-05	0.434	-0.6023421	9	0.9	KIF3C	Cytoskeleton
DMR2:26844001	2	26844001	26846000	2000	1	5.74E-05	0.465	0.4078971	34	1.7	DPYSL5	Metabolism
DMR2:27305001	2	27305001	27306000	1000	1	4.88E-05	0.443	-0.4845202	29	2.9	TRIM54;UCN;MPV17	Metabolism;Signaling;Unknown
DMR2:94998001	2	94998001	9.50E+07	2000	1	3.02E-06	0.252	-0.6140203	72	3.6	;RN7SL575P	
DMR2:95044001	2	95044001	95045000	1000	1	6.51E-05	0.466	-0.5473878	10	1	MAL;	Development
DMR2:103500001	2	103500001	103501000	1000	1	1.25E-05	0.33	0.6404451	4	0.4	CRLF3P1	
DMR2:109013001	2	109013001	109014000	1000	1	9.12E-05	0.49	-0.5867481	16	1.6		
DMR2:125560001	2	125560001	125561000	1000	1	7.89E-05	0.49	0.6196561	9	0.9		
DMR2:127624001	2	127624001	127626000	2000	1	2.46E-05	0.402	-0.6260796	55	2.75	MYO7B;	Cytoskeleton
DMR2:130498001	2	130498001	130499000	1000	1	8.32E-06	0.305	-0.6641899	10	1	POTEI;RNU6-473P	
DMR2:156263001	2	156263001	156264000	1000	1	3.39E-05	0.424	-0.5378893	6	0.6	LINC01876	
DMR2:161219001	2	161219001	161220000	1000	1	7.87E-07	0.244	0.6821073	1	0.1	TANK;	Signaling
DMR2:171191001	2	171191001	171192000	1000	1	9.69E-05	0.49	-0.5601089	7	0.7	TLK1	Signaling
DMR2:188002001	2	188002001	188003000	1000	1	7.18E-05	0.473	0.3800487	8	0.8	LINC01090	
DMR2:189031001	2	189031001	189032000	1000	1	4.13E-05	0.434	0.4195576	7	0.7	COL5A2	Extracellular Matrix
DMR2:189742001	2	189742001	189743000	1000	1	7.62E-05	0.489	0.9364422	4	0.4	ANKAR;OSGEPL1	Proteolysis
DMR2:194895001	2	194895001	194896000	1000	1	5.63E-06	0.281	0.697284	4	0.4		
DMR2:215333001	2	215333001	215334000	1000	1	4.46E-05	0.436	0.449268	15	1.5	ATIC	Metabolism
DMR3:12124001	3	12124001	12125000	1000	1	8.16E-05	0.49	0.4454555	7	0.7	SYN2	Cytoskeleton
DMR3:33269001	3	33269001	33270000	1000	1	4.26E-05	0.434	-0.5553829	7	0.7	FBXL2	Proteolysis
DMR3:35588001	3	35588001	35589000	1000	1	5.69E-05	0.464	0.6094043	9	0.9		
DMR3:57509001	3	57509001	57510000	1000	1	8.45E-06	0.305	0.5448255	11	1.1	DNAH12;RNU6-1181P;RNF7P1	Cytoskeleton
DMR3:88592001	3	88592001	88594000	2000	1	7.67E-05	0.489	0.4673591	11	0.55	NDUFA5P5	
DMR3:90614001	3	90614001	90615000	1000	1	7.34E-06	0.305	0.4712246	11	1.1		
DMR3:91949001	3	91949001	91950000	1000	1	6.64E-05	0.466	-0.6094188	19	1.9		
DMR3:123504001	3	123504001	123505000	1000	1	4.35E-05	0.434	0.5421266	8	0.8	HACD2	
DMR3:127606001	3	127606001	127608000	2000	1	9.72E-05	0.49	-0.4445336	45	2.25	TPRA1;MCM2	Cell Cycle
DMR3:127870001	3	127870001	127871000	1000	1	3.21E-05	0.424	0.6878869	12	1.2	MGLL	Metabolism

DMR3:145601001	3	145601001	145602000	1000	1	5.90E-05	0.465	0.568916	8	0.8		
DMR3:146626001	3	146626001	146628000	2000	1	1.46E-05	0.33	0.533427	15	0.75		
DMR3:146754001	3	146754001	146755000	1000	1	8.19E-05	0.49	0.5168656	6	0.6		
DMR3:148229001	3	148229001	148231000	2000	1	9.99E-05	0.49	0.3800089	19	0.95	;LINC02045	
DMR3:149896001	3	149896001	149897000	1000	1	9.62E-05	0.49	0.6340834	9	0.9	ANKUB1;RNF13	
DMR3:169738001	3	169738001	169739000	1000	1	8.60E-05	0.49	0.5051137	5	0.5		
DMR3:171910001	3	171910001	171911000	1000	1	6.70E-05	0.466	0.5419197	4	0.4	TMEM212-AS1;TMEM212	
DMR4:1844001	4	1844001	1845000	1000	1	9.65E-05	0.49	-0.4356277	23	2.3	LETM1	Signaling
DMR4:7430001	4	7430001	7431000	1000	1	3.15E-05	0.424	-0.7996396	25	2.5	SORCS2;PSAPL1	Receptor;Unknown
DMR4:20822001	4	20822001	20823000	1000	1	8.39E-05	0.49	-0.7095898	5	0.5	KCNIP4	Metabolism
DMR4:24522001	4	24522001	24524000	2000	1	2.45E-06	0.252	0.5606826	16	0.8	DHX15;MIR573	Transcription
DMR4:28539001	4	28539001	28541000	2000	1	6.72E-05	0.466	0.5173392	2	0.1	NA	
DMR4:83659001	4	83659001	83661000	2000	1	6.27E-05	0.466	0.4847344	17	0.85	NA	
DMR4:86427001	4	86427001	86428000	1000	1	4.35E-05	0.434	0.4856609	18	1.8	MAPK10	Signaling
DMR4:87375001	4	87375001	87376000	1000	1	6.32E-05	0.466	0.4423445	19	1.9	HSD17B11	Metabolism
DMR4:97619001	4	97619001	97620000	1000	1	3.60E-06	0.252	0.5263726	8	0.8	STPG2;	Development
DMR4:101962001	4	101962001	101963000	1000	1	7.47E-06	0.305	0.721593	4	0.4	BANK1;MTND5P5	Development
DMR4:103958001	4	103958001	103959000	1000	1	5.01E-05	0.449	0.5202654	5	0.5	LINC02503	
DMR4:131503001	4	131503001	131504000	1000	1	9.76E-05	0.49	0.5765546	16	1.6	LINC02377	
DMR4:156095001	4	156095001	156097000	2000	1	5.22E-05	0.455	0.5049367	24	1.2		
DMR4:157349001	4	157349001	157350000	1000	1	1.15E-06	0.252	0.6092699	5	0.5	GRIA2	Receptor
DMR4:167078001	4	167078001	167079000	1000	1	1.07E-05	0.325	0.8824477	4	0.4	SPOCK3	Signaling
DMR4:170255001	4	170255001	170256000	1000	1	1.44E-05	0.33	-0.5232598	8	0.8	LINC01612	
DMR5:1322001	5	1322001	1324000	2000	1	6.84E-05	0.467	-0.6052998	54	2.7	CLPTM1L	Unknown
DMR5:4825001	5	4825001	4826000	1000	1	9.74E-05	0.49	0.4012405	8	0.8	NA	
DMR5:8983001	5	8983001	8984000	1000	1	4.92E-05	0.444	0.5154234	10	1	NA	
DMR5:10591001	5	10591001	10592000	1000	1	6.00E-05	0.465	-0.57526	8	0.8	ANKRD33B	Transcription
DMR5:13961001	5	13961001	13962000	1000	1	9.20E-05	0.49	-0.4740341	15	1.5	DNAH5	Cytoskeleton
DMR5:19278001	5	19278001	19280000	2000	1	9.95E-05	0.49	0.6531139	11	0.55		
DMR5:24628001	5	24628001	24629000	1000	1	9.15E-05	0.49	0.4827595	5	0.5	CDH10	Extracellular Matrix
DMR5:27835001	5	27835001	27837000	2000	1	4.46E-10	0.001	0.5887458	42	2.1		
DMR5:50747001	5	50747001	50749000	2000	1	2.57E-05	0.404	0.5232029	22	1.1	PARP8	Unknown
DMR5:66663001	5	66663001	66664000	1000	1	5.46E-05	0.46	0.4803489	4	0.4	MAST4;MAST4-IT1	Cytoskeleton
DMR5:72925001	5	72925001	72927000	2000	1	8.83E-05	0.49	-0.5402532	57	2.85	TNPO1	Metabolism
DMR5:102518001	5	102518001	102519000	1000	1	1.12E-05	0.33	0.5931841	13	1.3		
DMR5:106377001	5	106377001	106378000	1000	1	1.98E-05	0.359	0.5327325	5	0.5		
DMR5:107497001	5	107497001	107498000	1000	1	3.62E-05	0.427	-0.5078895	12	1.2	EFNA5	Signaling
DMR5:122924001	5	122924001	122926000	2000	1	6.47E-05	0.466	0.4099698	12	0.6	SNX24	
DMR5:146915001	5	146915001	146916000	1000	1	2.65E-05	0.408	0.4426619	5	0.5	PPP2R2B;PPP2R2B-IT1	Signaling
DMR5:168831001	5	168831001	168832000	1000	1	5.60E-05	0.463	-0.4268654	17	1.7	SLIT3	Development
DMR5:177065001	5	177065001	177067000	2000	1	8.28E-05	0.49	-0.5165968	27	1.35	ZNF346	Transcription
DMR5:179779001	5	179779001	179781000	2000	1	9.90E-05	0.49	-0.694979	28	1.4	MAML1	Transcription
DMR6:20751001	6	20751001	20753000	2000	1	3.13E-05	0.424	0.4262769	15	0.75	CDKAL1;	Cell Cycle
DMR6:33744001	6	33744001	33746000	2000	1	4.83E-06	0.252	-0.5812982	31	1.55	IP6K3	Signaling
DMR6:34787001	6	34787001	34788000	1000	1	5.54E-05	0.463	0.4550965	8	0.8	UHRF1BP1	
DMR6:36969001	6	36969001	36972000	3000	1	9.09E-05	0.49	-0.7448019	57	1.9	PI16;MTCH1	Binding Protein
DMR6:43392001	6	43392001	43393000	1000	1	9.48E-05	0.49	-0.6298097	9	0.9	NA	
DMR6:57011001	6	57011001	57012000	1000	1	5.39E-05	0.458	0.5356632	9	0.9	BEND6;FTH1P15	
DMR6:79738001	6	79738001	79739000	1000	1	1.04E-05	0.323	0.5154463	10	1		
DMR6:80356001	6	80356001	80357000	1000	1	3.68E-05	0.427	0.6585701	6	0.6	BCKDHB;	Metabolism
DMR6:94043001	6	94043001	94044000	1000	1	9.01E-06	0.31	0.4588388	4	0.4		
DMR6:103058001	6	103058001	103059000	1000	1	9.64E-05	0.49	0.4358077	3	0.3		
DMR6:125239001	6	125239001	125240000	1000	1	3.68E-06	0.252	-0.6990496	6	0.6	TPD52L1;HDDC2	
DMR6:129292001	6	129292001	129293000	1000	1	1.85E-05	0.356	0.5333206	23	2.3	LAMA2	Extracellular Matrix
DMR6:158624001	6	158624001	158627000	3000	1	6.55E-05	0.466	-0.5267842	72	2.4	TMEM181;TATDN2P2;DYNLT1	Development
DMR7:46001	7	46001	47000	1000	1	7.32E-05	0.481	-0.5566658	14	1.4	NA	
DMR7:2834001	7	2834001	2835000	1000	1	2.11E-06	0.252	-0.7654053	20	2	GNA12;	Signaling
DMR7:11541001	7	11541001	11542000	1000	1	6.64E-06	0.305	0.5375895	11	1.1	THSD7A	Extracellular Matrix
DMR7:13472001	7	13472001	13473000	1000	1	5.79E-05	0.465	0.6015962	8	0.8	NA	
DMR7:19680001	7	19680001	19681000	1000	1	7.03E-05	0.472	0.6490453	4	0.4		
DMR7:33414001	7	33414001	33415000	1000	1	3.08E-06	0.252	0.5999412	3	0.3	BBS9	Development
DMR7:45082001	7	45082001	45083000	1000	1	3.17E-05	0.424	-0.5246614	21	2.1	CCM2;NACAD	Development;Transcription
DMR7:49664001	7	49664001	49665000	1000	1	6.77E-05	0.466	-0.7386902	4	0.4		
DMR7:52687001	7	52687001	52688000	1000	1	1.59E-05	0.343	0.5714602	10	1		
DMR7:54907001	7	54907001	54908000	1000	1	5.18E-05	0.454	0.4644917	13	1.3		
DMR7:101088001	7	101088001	101090000	2000	1	9.40E-05	0.49	-0.4524769	94	4.7	TRIM56	Metabolism
DMR7:107390001	7	107390001	107392000	2000	1	5.17E-05	0.454	0.6849712	27	1.35	COG5	Golgi

DMR7:128291001	7	128291001	128293000	2000	1	7.49E-05	0.488	-0.5085499	35	1.75	RBM28	Transcription
DMR7:132155001	7	132155001	132157000	2000	1	9.63E-05	0.49	-0.404515	29	1.45	PLXNA4	Receptor
DMR7:132328001	7	132328001	132329000	1000	1	3.38E-05	0.424	-0.5754199	19	1.9	PLXNA4	Receptor
DMR7:152266001	7	152266001	152268000	2000	1	6.64E-05	0.466	0.3616713	18	0.9	KMT2C	Transcription
DMR7:158534001	7	158534001	158536000	2000	1	1.36E-05	0.33	-0.5229854	41	2.05	PTPRN2;MIR595;	Signaling
DMR8:4645001	8	4645001	4646000	1000	1	5.78E-05	0.465	0.5159451	14	1.4	CSMD1;	Signaling
DMR8:16328001	8	16328001	16329000	1000	1	3.52E-05	0.425	0.5903423	8	0.8	MSR1	Receptor
DMR8:28651001	8	28651001	28652000	1000	1	1.26E-05	0.33	-0.5980499	10	1	EXTL3	Metabolism
DMR8:34995001	8	34995001	34996000	1000	1	2.47E-06	0.252	0.7259194	9	0.9		
DMR8:53610001	8	53610001	53612000	2000	1	3.93E-05	0.434	-0.5323158	24	1.2		
DMR8:73605001	8	73605001	73606000	1000	1	7.71E-06	0.305	0.6554974	9	0.9	STAU2	Transcription
DMR8:77947001	8	77947001	77948000	1000	1	1.34E-05	0.33	0.7489796	4	0.4		
DMR8:90648001	8	90648001	90649000	1000	1	1.62E-06	0.252	0.6802861	6	0.6	LINC00534;TMEM64;	
DMR8:100881001	8	100881001	100882000	1000	1	2.93E-05	0.424	-0.5538749	10	1		
DMR8:109325001	8	109325001	109326000	1000	1	9.67E-06	0.319	0.4401641	7	0.7	NUDCD1;;ENY2	
DMR8:127933001	8	127933001	127937000	4000	1	7.79E-06	0.305	-0.6113981	54	1.35	PVT1;TMEM75	
DMR8:130826001	8	130826001	130827000	1000	1	9.93E-05	0.49	0.5675709	7	0.7	ADCY8	Signaling
DMR8:142277001	8	142277001	142279000	2000	1	7.19E-06	0.305	-0.6854672	52	2.6	TSNARE1	Transport
DMR8:142473001	8	142473001	142475000	2000	1	5.63E-05	0.463	-0.6838394	55	2.75	ADGRB1	
DMR8:143884001	8	143884001	143885000	1000	1	1.88E-05	0.356	-0.5543847	31	3.1	EPPK1;	Unknown
DMR8:144097001	8	144097001	144098000	1000	1	2.31E-05	0.389	-0.9496952	11	1.1	CYC1;SHARPIN;MAF1;WDR97	Metabolism;Apoptosis
DMR9:4467001	9	4467001	4468000	1000	1	8.17E-06	0.305	-0.729959	19	1.9		
DMR9:8676001	9	8676001	8677000	1000	1	7.91E-05	0.49	0.4330407	18	1.8	PTPRD	Signaling
DMR9:33007001	9	33007001	33008000	1000	1	2.41E-05	0.398	0.466193	7	0.7	APTX	
DMR9:63460001	9	63460001	63462000	2000	1	1.84E-05	0.356	0.4558814	17	0.85		
DMR9:70103001	9	70103001	70104000	1000	1	3.08E-05	0.424	0.5516958	17	1.7	MAMDC2-AS1;MAMDC2	
DMR9:83554001	9	83554001	83555000	1000	1	1.49E-05	0.33	0.7436915	8	0.8		
DMR9:93088001	9	93088001	93089000	1000	1	8.60E-05	0.49	-0.4880882	15	1.5	SUSD3;;CARD19	Development
DMR9:98170001	9	98170001	98171000	1000	1	5.05E-05	0.45	-0.4523153	13	1.3	CORO2A	Cytoskeleton
DMR9:101153001	9	101153001	101154000	1000	1	9.12E-05	0.49	0.5075236	13	1.3	PLPPR1	
DMR9:110419001	9	110419001	110420000	1000	1	8.74E-05	0.49	0.4759732	7	0.7	SVEP1	Unknown
DMR9:117877001	9	117877001	117878000	1000	1	9.12E-05	0.49	0.3905077	8	0.8	NA	
DMR9:130203001	9	130203001	130205000	2000	1	1.97E-05	0.359	-0.513214	37	1.85	NCS1	Signaling
DMR9:134346001	9	134346001	134347000	1000	1	6.80E-05	0.467	-0.5090012	24	2.4	RXRA	Receptor
DMR9:136499001	9	136499001	136501000	2000	1	5.30E-05	0.458	-0.6293416	100	5	NOTCH1	Signaling
DMR9:136805001	9	136805001	136807000	2000	1	3.33E-05	0.424	-0.5234165	49	2.45	;CCDC183;CCDC183-AS1;RABL6;NCLP1	
DMR10:1444001	10	1444001	1448000	4000	1	2.56E-06	0.252	-0.7359738	35	0.875	ADARB2	Metabolism
DMR10:67927001	10	67927001	67928000	1000	1	1.21E-05	0.33	0.4985588	12	1.2	SIRT1;HERC4	Transcription;Metabolism
DMR10:79146001	10	79146001	79148000	2000	1	1.23E-05	0.33	-0.7569266	33	1.65	ZMIZ1	Metabolism
DMR10:80732001	10	80732001	80734000	2000	1	5.98E-05	0.465	-0.5565321	24	1.2		
DMR10:81559001	10	81559001	81560000	1000	1	3.31E-05	0.424	0.5773713	7	0.7		
DMR10:98739001	10	98739001	98740000	1000	1	3.80E-06	0.252	0.6466657	5	0.5	HPSE2	Metabolism
DMR10:106464001	10	106464001	106466000	2000	1	4.01E-05	0.434	0.5721697	9	0.45		
DMR10:117684001	10	117684001	117685000	1000	1	6.74E-05	0.466	-0.5109539	36	3.6		
DMR10:126928001	10	126928001	126929000	1000	1	7.15E-05	0.473	-0.5710495	21	2.1	DOCK1	Signaling
DMR10:131011001	10	131011001	131012000	1000	1	9.61E-05	0.49	-0.4625415	16	1.6		
DMR11:292001	11	292001	294000	2000	1	9.00E-05	0.49	-0.4805332	60	3	NLRP6;;PGGHG;IFITM5;IFITM2	Unknown
DMR11:1372001	11	1372001	1373000	1000	1	4.37E-05	0.434	-0.5562553	31	3.1	LINC02689	
DMR11:9885001	11	9885001	9886000	1000	1	4.10E-05	0.434	0.5288887	16	1.6	SBF2;	Epigenetic
DMR11:15529001	11	15529001	15530000	1000	1	9.64E-05	0.49	0.4165148	13	1.3		
DMR11:19529001	11	19529001	19530000	1000	1	9.01E-05	0.49	-0.5343288	13	1.3	NAV2;NAV2-AS4	Development
DMR11:30838001	11	30838001	30839000	1000	1	3.80E-05	0.432	0.6553675	11	1.1	;DCDC1	
DMR11:32991001	11	32991001	32992000	1000	1	7.58E-05	0.489	0.411216	11	1.1	QSER1	Development
DMR11:44999001	11	44999001	4.50E+07	1000	1	3.96E-05	0.434	-0.7255403	6	0.6	NA	
DMR11:53757001	11	53757001	53758000	1000	1	6.58E-05	0.466	-1.1979892	11	1.1		
DMR11:73573001	11	73573001	73574000	1000	1	4.31E-05	0.434	-0.4753193	12	1.2	FAM168A;HMGN2P38	
DMR11:74434001	11	74434001	74435000	1000	1	1.67E-05	0.354	0.6121904	31	3.1	NA	
DMR11:75556001	11	75556001	75557000	1000	1	8.35E-05	0.49	-0.5689503	11	1.1	SERPINH1	Development
DMR11:85403001	11	85403001	85404000	1000	1	6.24E-05	0.466	0.4383701	7	0.7	DLG2	
DMR11:113616001	11	113616001	113617000	1000	1	4.86E-05	0.443	0.5821733	4	0.4		
DMR11:114151001	11	114151001	114152000	1000	1	8.77E-05	0.49	-0.6885538	7	0.7	ZBTB16;	Transcription
DMR11:125254001	11	125254001	125256000	2000	1	9.60E-05	0.49	-0.6253659	27	1.35	PKNOX2;	Transcription
DMR12:2324001	12	2324001	2326000	2000	1	9.89E-06	0.32	-0.5779211	37	1.85	CACNA1C;	Metabolism
DMR12:12257001	12	12257001	12258000	1000	1	6.99E-05	0.472	0.3819639	23	2.3	LRP6;	Receptor
DMR12:41370001	12	41370001	41371000	1000	1	3.07E-06	0.252	0.7031864	5	0.5	PDZRN4	
DMR12:44695001	12	44695001	44696000	1000	1	4.49E-05	0.436	0.508272	4	0.4	NELL2	Signaling
DMR12:52552001	12	52552001	52553000	1000	1	2.42E-06	0.252	-0.6977243	19	1.9	KRT71	Cytoskeleton

DMR12:74144001	12	74144001	74145000	1000	1	4.13E-06	0.252	0.6500154	17	1.7	LINC02882	
DMR12:76922001	12	76922001	76923000	1000	1	1.77E-05	0.356	0.5780447	6	0.6		
DMR12:107378001	12	107378001	107379000	1000	1	2.99E-05	0.424	-0.7047451	8	0.8	BTBD11;	Signaling
DMR12:113159001	12	113159001	113161000	2000	1	6.28E-05	0.466	-0.4918423	34	1.7	CFAP73;Y_RNA;DDX54;MIR7106	Transcription
DMR12:114524001	12	114524001	114525000	1000	1	7.54E-05	0.489	-0.4561312	16	1.6		
DMR12:119174001	12	119174001	119176000	2000	1	8.18E-05	0.49	-0.6341767	23	1.15	HSPB8;	Signaling
DMR12:125711001	12	125711001	125712000	1000	1	7.03E-05	0.472	-0.5852154	6	0.6		
DMR13:22667001	13	22667001	22668000	1000	1	4.66E-06	0.252	0.6819355	10	1		
DMR13:53514001	13	53514001	53515000	1000	1	3.22E-06	0.252	0.4534399	10	1	NA	
DMR13:57219001	13	57219001	57220000	1000	1	2.25E-05	0.386	0.6521984	11	1.1		
DMR13:66071001	13	66071001	66072000	1000	1	3.65E-05	0.427	0.5026316	12	1.2		
DMR13:66552001	13	66552001	66553000	1000	1	2.11E-05	0.371	-0.6778826	9	0.9	PCDH9	Extracellular Matrix
DMR13:89023001	13	89023001	89024000	1000	1	1.36E-05	0.33	-0.6114195	6	0.6		
DMR13:100909001	13	100909001	100910000	1000	1	3.13E-05	0.424	0.4889688	5	0.5	NALCN-AS1	
DMR13:104893001	13	104893001	104894000	1000	1	6.20E-05	0.466	0.5458221	21	2.1		
DMR13:109824001	13	109824001	109825000	1000	1	2.23E-05	0.386	-0.7182686	5	0.5		
DMR14:22756001	14	22756001	22757000	1000	1	6.06E-05	0.466	0.4065444	14	1.4	;OXA1L	Transport
DMR14:28721001	14	28721001	28722000	1000	1	7.13E-05	0.473	0.4583643	7	0.7	FOXG1-AS1	
DMR14:52647001	14	52647001	52648000	1000	1	6.74E-05	0.466	0.4092472	13	1.3	GPR137C;ERO1A;	
DMR14:69068001	14	69068001	69069000	1000	1	7.73E-05	0.489	0.5936761	8	0.8	DCAF5	Signaling
DMR14:70017001	14	70017001	70018000	1000	1	7.94E-05	0.49	-0.561734	9	0.9	SMOC1	Receptor
DMR14:76378001	14	76378001	76379000	1000	1	5.91E-05	0.465	-0.6607036	11	1.1	ESRRB	
DMR14:93377001	14	93377001	93378000	1000	1	3.29E-05	0.424	0.535412	17	1.7	UNC79	
DMR14:94820001	14	94820001	94821000	1000	1	4.87E-06	0.252	-0.7680331	4	0.4		
DMR14:100535001	14	100535001	100536000	1000	1	4.72E-05	0.443	-0.7278778	17	1.7	WDR25;BEGAIN;	
DMR14:104288001	14	104288001	104289000	1000	1	6.66E-05	0.466	-0.5016346	23	2.3	LINC02691;	
DMR14:104587001	14	104587001	104592000	5000	1	3.87E-05	0.432	-0.5510328	169	3.38	TMEM179;C14orf180;	Unknown
DMR14:105556001	14	105556001	105558000	2000	1	4.20E-05	0.434	-0.4139408	65	3.25	ELK2BP	
DMR15:21020001	15	21020001	21021000	1000	1	4.89E-05	0.443	-0.825802	13	1.3	LINC01193;IGHD5OR15-5B;IGHD4OR15-4B;IGHD3OR15-3B;IGHD2OR15-2B;IGHD1OR15-1B;FAM30C	
DMR15:28604001	15	28604001	28607000	3000	1	1.93E-05	0.359	-0.5299286	27	0.9	HERC2P9	
DMR15:31159001	15	31159001	31161000	2000	1	9.54E-05	0.49	-0.6441002	37	1.85	TRPM1	Receptor
DMR15:63635001	15	63635001	63637000	2000	1	1.39E-05	0.33	-0.4571604	17	0.85	HERC1	Signaling
DMR15:67505001	15	67505001	67506000	1000	1	1.46E-05	0.33	0.4749365	9	0.9	IQCH;IQCH-AS1	
DMR15:71212001	15	71212001	71213000	1000	1	3.54E-05	0.425	-0.7833944	11	1.1	THSD4	Extracellular Matrix
DMR15:72064001	15	72064001	72065000	1000	1	1.39E-05	0.33	0.498003	9	0.9	MYO9A	Cytoskeleton
DMR15:73726001	15	73726001	73729000	3000	1	6.87E-06	0.305	-0.6744791	28	0.933	;INSYN1	
DMR15:85873001	15	85873001	85874000	1000	1	3.52E-05	0.425	-0.6243181	10	1		
DMR15:90880001	15	90880001	90881000	1000	1	9.46E-05	0.49	-0.5164857	40	4	FURIN;FES	Transcription;Signaling
DMR15:92439001	15	92439001	92443000	4000	1	8.71E-05	0.49	-0.7082545	51	1.275	ST8SIA2	Metabolism
DMR15:93149001	15	93149001	93151000	2000	1	1.18E-05	0.33	-0.512812	28	1.4		
DMR15:93978001	15	93978001	93979000	1000	1	3.37E-05	0.424	0.424356	7	0.7	LINC01579;LINC01580;LINC01581	
DMR15:97826001	15	97826001	97827000	1000	1	8.04E-05	0.49	0.5247666	4	0.4	LINC00923	
DMR15:99640001	15	99640001	99642000	2000	1	3.18E-07	0.164	0.5164142	40	2	MEF2A	Transcription
DMR16:1327001	16	1327001	1328000	1000	1	4.30E-06	0.252	-0.9116299	23	2.3	UBE2I;;RPS20P2;BAIAP3	Metabolism
DMR16:3962001	16	3962001	3963000	1000	1	8.69E-06	0.306	-0.6626307	17	1.7	ADCY9	Metabolism
DMR16:14765001	16	14765001	14766000	1000	1	3.55E-05	0.425	-0.4353565	14	1.4	NPIPA2;	
DMR16:16470001	16	16470001	16471000	1000	1	3.22E-05	0.424	0.5198837	6	0.6		
DMR16:34460001	16	34460001	34462000	2000	1	8.81E-05	0.49	0.4552193	10	0.5		
DMR16:51228001	16	51228001	51229000	1000	1	8.64E-05	0.49	0.5846113	8	0.8	NA	
DMR16:56287001	16	56287001	56289000	2000	1	1.85E-05	0.356	-0.8051568	27	1.35	GNAO1;	Signaling
DMR16:76935001	16	76935001	76936000	1000	1	2.03E-06	0.252	0.4804988	11	1.1	NA	
DMR17:11486001	17	11486001	11487000	1000	1	8.09E-06	0.305	-0.5411112	17	1.7	SHISA6	Development
DMR17:19051001	17	19051001	19053000	2000	1	5.15E-05	0.454	-0.5294399	33	1.65	GRAP;SNORD3B-1	Signaling
DMR17:29829001	17	29829001	29830000	1000	1	3.16E-05	0.424	-0.6031779	10	1	SSH2	Signaling
DMR17:41886001	17	41886001	41887000	1000	1	4.36E-05	0.434	-0.5764436	16	1.6	ACLY	Metabolism
DMR17:50168001	17	50168001	50170000	2000	1	4.56E-05	0.439	-0.617355	35	1.75	;SGCA;H1-9P	Signaling
DMR17:70291001	17	70291001	70292000	1000	1	1.69E-05	0.354	0.5402442	11	1.1		
DMR17:74102001	17	74102001	74103000	1000	1	9.93E-05	0.49	-0.4916762	16	1.6	LINC02074	
DMR17:74256001	17	74256001	74259000	3000	1	6.01E-05	0.465	-0.54418	48	1.6	TTYH2;	Transport
DMR17:76081001	17	76081001	76082000	1000	1	4.63E-05	0.44	-0.5493264	26	2.6	SRP68;ZACN;GALR2;EXOC7;	Transcription;Receptor;Transport
DMR17:76407001	17	76407001	76408000	1000	1	5.65E-05	0.463	-0.5635302	16	1.6	UBE2O	Proteolysis

DMR17:80452001	17	80452001	80453000	1000	1	3.16E-05	0.424	-0.6411466	20	2	NA	
DMR18:13673001	18	13673001	13676000	3000	1	8.32E-05	0.49	0.3295726	37	1.233	FAM210A	
DMR18:22066001	18	22066001	22069000	3000	1	7.38E-05	0.482	-0.4419002	29	0.967		
DMR18:26302001	18	26302001	26304000	2000	1	4.44E-06	0.252	0.6205914	9	0.45	TAF4B	Transcription
DMR18:29029001	18	29029001	29031000	2000	1	7.69E-05	0.489	0.6209582	16	0.8		
DMR18:35790001	18	35790001	35792000	2000	1	5.39E-05	0.458	-0.5291051	31	1.55		
DMR18:62829001	18	62829001	62830000	1000	1	2.56E-05	0.404	0.571388	14	1.4	PHLPP1	Signaling
DMR18:78500001	18	78500001	78504000	4000	1	3.37E-05	0.424	-0.6377108	165	4.125	NA	
DMR19:1796001	19	1796001	1797000	1000	1	4.69E-05	0.443	-0.9701297	72	7.2	ATP8B3	Metabolism
DMR19:8262001	19	8262001	8263000	1000	1	8.18E-05	0.49	-0.6457656	16	1.6	CERS4	
DMR19:10089001	19	10089001	10090000	1000	1	7.14E-05	0.473	-0.5034229	16	1.6	SHFL;;ANGPTL6	
DMR19:29292001	19	29292001	29294000	2000	1	1.54E-05	0.337	-0.8621611	28	1.4	;VSTM2B-DT	
DMR19:31563001	19	31563001	31564000	1000	1	2.66E-05	0.408	-0.6344226	25	2.5	LINC02841	
DMR19:31729001	19	31729001	31730000	1000	1	9.62E-05	0.49	-0.6605847	7	0.7		
DMR19:32108001	19	32108001	32109000	1000	1	4.48E-05	0.436	-0.5384374	10	1	LINC01782;	
DMR19:34808001	19	34808001	34809000	1000	1	6.38E-05	0.466	-0.779656	8	0.8	LINC01801;	
DMR19:44892001	19	44892001	44894000	2000	1	2.27E-05	0.386	-0.6023741	37	1.85	NECTIN2;;TOMM40	Metabolism
DMR19:47288001	19	47288001	47289000	1000	1	1.46E-05	0.33	-0.5307813	13	1.3	C5AR1	Receptor
DMR20:4900001	20	4900001	4903000	3000	1	6.00E-05	0.465	-0.6358696	42	1.4	SLC23A2	Transport
DMR20:11901001	20	11901001	11902000	1000	1	2.58E-05	0.404	-0.6988302	7	0.7	BTBD3;	Unknown
DMR20:14316001	20	14316001	14317000	1000	1	4.83E-05	0.443	0.5984049	3	0.3	MACROD2;FLRT3	Receptor
DMR20:24917001	20	24917001	24918000	1000	1	4.46E-07	0.173	-0.5047593	19	1.9		
DMR20:25309001	20	25309001	25310000	1000	1	2.53E-05	0.404	-0.7834703	18	1.8	ABHD12	
DMR20:42053001	20	42053001	42054000	1000	1	3.80E-05	0.432	-0.5893909	11	1.1	NA	
DMR20:46395001	20	46395001	46396000	1000	1	6.68E-05	0.466	-0.6385197	13	1.3	;ELMO2	Signaling
DMR20:61669001	20	61669001	61670000	1000	1	4.26E-06	0.252	-0.4953958	21	2.1	CDH4	Extracellular Matrix
DMR20:63217001	20	63217001	63218000	1000	1	7.62E-06	0.305	-0.634225	18	1.8	YTHDF1;	
DMR20:63465001	20	63465001	63466000	1000	1	7.72E-05	0.489	-0.4980048	52	5.2	KCNQ2	Metabolism
DMR21:13172001	21	13172001	13173000	1000	1	1.01E-06	0.252	0.5572377	9	0.9		
DMR21:19524001	21	19524001	19525000	1000	1	5.40E-05	0.458	0.4401356	8	0.8		
DMR21:23101001	21	23101001	23102000	1000	1	7.81E-05	0.49	0.5611094	25	2.5	;ZNF299P;MSANTD2P1	
DMR21:33413001	21	33413001	33414000	1000	1	3.81E-05	0.432	-0.5503782	14	1.4	IFNGR2	Receptor
DMR21:33599001	21	33599001	33600000	1000	1	4.24E-05	0.434	0.5610996	3	0.3	;CRYZL1	Metabolism
DMR22:17153001	22	17153001	17154000	1000	1	9.45E-05	0.49	-0.5285131	8	0.8	HDHD5;HDHD5-AS1	
DMR22:19767001	22	19767001	19768000	1000	1	1.82E-05	0.356	-0.7138155	57	5.7	TBX1	Epigenetic
DMR22:21094001	22	21094001	21095000	1000	1	8.66E-05	0.49	-0.5697769	14	1.4	BCRP2	
DMR22:23154001	22	23154001	23155000	1000	1	1.18E-05	0.33	-0.4409129	14	1.4	RSPH14;RAB36	Signaling
DMR22:24450001	22	24450001	24451000	1000	1	8.98E-05	0.49	-0.5971111	8	0.8	SPECC1L-ADORA2A;ADORA2A;ADORA2A-AS1	Receptor
DMR22:36828001	22	36828001	36829000	1000	1	3.56E-05	0.425	-0.7258953	14	1.4	PVALB;	Receptor
DMR22:44134001	22	44134001	44135000	1000	1	6.35E-05	0.466	-0.6609342	24	2.4	PARVB;	Cytoskeleton
DMR22:47284001	22	47284001	47285000	1000	1	2.01E-05	0.359	-0.516009	17	1.7		
DMRX:823001	X	823001	825000	2000	1	4.50E-05	0.436	0.6375235	22	1.1		
DMRX:1070001	X	1070001	1073000	3000	1	5.40E-05	0.458	0.395875	71	2.367		
DMRX:1167001	X	1167001	1169000	2000	1	3.29E-05	0.424	0.5079069	22	1.1		
DMRX:6391001	X	6391001	6393000	2000	1	3.12E-05	0.424	0.3812518	23	1.15	MIR4770	
DMRX:28676001	X	28676001	28677000	1000	1	1.81E-05	0.356	0.5730431	12	1.2	IL1RAPL1	Receptor
DMRX:34503001	X	34503001	34504000	1000	1	6.29E-05	0.466	0.4515925	7	0.7		
DMRX:40330001	X	40330001	40331000	1000	1	4.22E-05	0.434	0.4014892	13	1.3		
DMRX:41499001	X	41499001	41500000	1000	1	1.88E-05	0.356	-0.6249813	13	1.3		
DMRX:54926001	X	54926001	54927000	1000	1	3.53E-05	0.425	-0.7622447	9	0.9	TRO;SNORA11G;PFKFB1	Extracellular Matrix
DMRX:86184001	X	86184001	86185000	1000	1	2.00E-05	0.359	0.5720155	9	0.9	DACH2	Development
DMRX:132103001	X	132103001	132104000	1000	1	4.87E-05	0.443	0.5267478	8	0.8	FRMD7	
DMRX:153476001	X	153476001	153477000	1000	1	1.48E-05	0.33	-0.6095855	17	1.7	ECMXP;TREX2;HAUS7	Transcription
DMRY:56737001	Y	56737001	56738000	1000	1	4.33E-05	0.434	0.6092877	21	2.1		
