# Supplementary material for: Epigenome association study for DNA methylation biomarkers in buccal and monocyte cells for female rheumatoid arthritis
Source: Sci Rep. 2021 Dec 10;11:23789. doi: 10.1038/s41598-021-03170-6 (PMC8664902; doi:10.1038/s41598-021-03170-6)
Supplement: Supplementary file 7 — Supplementary Table S5. [file 41598_2021_3170_MOESM7_ESM.pdf]

Supplemental Table S5
DMR Associated RA Gene Cellular Process Correlations

Cell Process Name	Total # of Neighbors	Gene Set Seed	Overlap	Percent Overlap	Overlapping Entities	p-value	Jaccard similarity
Protein regulators of immune response	4387	immune response	60	1	NFATC1;NFATC2;MSRA;MST1;CLEC16A;ADAR;PRKN;NGF;NOS1;NOS2;MIR194-1;FSTL1;NPPC;AR;AREG;ATG16L1;AKT1;BCL2;PVT1;NLRP6;PRKDC;EIF2AK2;RELA;TNFSF8;S100B;CCL22;CX3CL1;STAT3;STAT4;SMARCA4;SUMO2;SOX5;CTSS;CYLD;CYP19A1;VWF;E2F2;UBE2I;H19;FOXO1;FLT1;GPX4;GSN;CD83;DNAJB1;IL34;HIF1A;IL1RAP;IL2RB;ITGA9;TNC;LAMA3;EBI3;TNFRSF13C;KCNMA1;TANK;KLRD1;MECP2;ATG7;MMP2	1.83665E-16	0.013614704
Protein regulators of inflammatory response	4100	inflammatory response	57	1	NFATC1;NFATC2;MSRA;MST1;ADAR;PRKN;NGF;NOS1;NOS2;MIR194-1;FSTL1;NPPC;AR;AREG;PDE4D;ATG16L1;AKT1;BCL2;PVT1;NLRP6;EIF2AK2;RELA;TNFSF8;S100B;CCL22;CX3CL1;STAT3;STAT4;SLIT3;SMARCA4;SUMO3;SUMO2;SOX5;CTSS;CYLD;CYP19A1;VWF;UBE2I;H19;FOXO1;FLT1;GPX4;GSN;CD83;IL34;HIF1A;IL1RAP;MIR573;TNC;EBI3;KCNMA1;TANK;KLRD1;MECP2;ATG7;NPC2;MMP2	1.78743E-15	0.013824885
Protein regulators of innate immune response	2657	innate immune response	43	1	VWF;NFATC1;CNTRL;MST1;ADAR;PRKN;FOXO1;NGF;NOS1;NOS2;FSTL1;NPPC;AR;AREG;ATG16L1;AKT1;BCL2;GSN;NLRP6;PRKDC;EIF2AK2;RPS6KA2;IL34;HIF1A;RELA;IL1RAP;TNC;TNFSF8;S100B;CCL22;CX3CL1;STAT3;STAT4;SMARCA4;KCNMA1;TANK;MECP2;ATG7;NPC2;EYA4;MMP2;CTSS;CYLD	1.04095E-12	0.015961396
Protein regulators of cellular immune response	1261	cellular immune response	30	2	CD83;DNAJB1;IL34;MSRA;MST1;HIF1A;RELA;IL1RAP;IL2RB;PRKN;FOXO1;NGF;TNC;S100B;NOS2;FSTL1;CCL22;CX3CL1;STAT3;STAT4;EBI3;AR;ATG16L1;AKT1;BCL2;GSN;CTSS;PRKDC;EIF2AK2;CYP19A1	1.37163E-12	0.022883295
Protein regulators of neuroprotection	1462	neuroprotection	32	2	E2F2;RPS6KA2;DNAJB1;UBE2I;MSRA;HIF1A;RELA;H19;PRKN;FOXO1;FLT1;NGF;S100B;NOS1;NOS2;MIR194-1;CX3CL1;STAT3;AR;TNFRSF13C;PDE4D;AKT1;KCNMA1;GPX4;ATG7;BCL2;GSN;MMP2;CYLD;PRKDC;EIF2AK2;CYP19A1	1.71694E-12	0.021192053
Protein regulators of endothelial cell proliferation	1559	endothelial cell proliferation	32	2	VWF;E2F2;NFATC1;H19;FGF8;FOXO1;FLT1;NGF;NOS1;NOS2;FSTL1;NPPC;AR;AREG;AKT1;BCL2;PTPRK;PVT1;PRKDC;RPS6KA2;IL34;HIF1A;RELA;ITSN1;ITGA9;TNC;CX3CL1;STAT3;SLIT3;KCNMA1;MMP2;TBX1	9.70244E-12	0.019912881
Protein regulators of cell mediated cytotoxicity	3407	cell mediated cytotoxicity	47	1	NFATC1;NFATC2;MSRA;MST1;ADAR;PRKN;NGF;NOS1;NOS2;FSTL1;NPPC;AR;AREG;AKT1;BCL2;PVT1;PRKDC;EIF2AK2;RAD51B;RELA;S100B;CCL22;CX3CL1;STAT3;STAT4;SMARCA4;SUMO2;TCN2;CTSS;CYLD;CYP19A1;VWF;FOXO1;FLT1;FOLR2;GPX4;GSN;CD83;DNAJB1;HIF1A;IL1RAP;IL2RB;TNC;TNFRSF13C;KLRD1;ATG7;MMP2	1.42458E-11	0.013662791
Protein regulators of immunological tolerance	818	immunological tolerance	23	2	VWF;E2F2;CD83;NFATC2;IL34;HIF1A;CLEC16A;RELA;IL2RB;ADAR;FOXO1;NGF;TNFSF8;NOS2;CCL22;STAT3;EBI3;TNFRSF13C;BANK1;SMARCA4;AKT1;BCL2;CYLD	4.56978E-11	0.026285714
Protein regulators of cell infiltration	1343	cell infiltration	28	2	CD83;CNTRL;HIF1A;RELA;PRKN;ITGA9;FLT1;NGF;TNC;NOS1;NOS2;FSTL1;NPPC;CCL22;CX3CL1;STAT3;STAT4;EBI3;AR;AREG;SMARCA4;AKT1;GPX4;ATG7;BCL2;MMP2;CTSS;CYLD	2.28793E-10	0.020071685
Protein regulators of adaptive immune response	1364	adaptive immune response	28	2	VWF;CD83;NFATC2;IL34;MST1;HIF1A;RELA;IL1RAP;PRKN;FOXO1;NGF;TNC;TNFSF8;S100B;NOS2;CCL22;CX3CL1;STAT3;STAT4;EBI3;AR;AREG;SMARCA4;ATG16L1;AKT1;NLRP6;CTSS;CYLD	3.27702E-10	0.019774011

Protein regulators of T-cell activation	1932	T-cell activation	33	1	VWF;NFATC1;NFATC2;UBE2I;FOXO1;FLT1;NGF;NOS1;NOS2;FOLR2;ATG16L1;AKT1;BCL2;EIF2AK2;CD83;HIF1A;RELA;IL2RB;TNC;TNFSF8;CCL22;STAT3;STAT4;EBI3;TNFRSF13C;SMARCA4;SUMO3;SUMO2;KLRD1;ATG7;MMP2;CTSS;CYLD	5.59043E-10	0.016675088
Protein regulators of macrophage function	932	macrophage function	23	2	IL34;MST1;HIF1A;ADAR;FOXO1;FLT1;NGF;TNC;S100B;NOS1;NOS2;CCL22;CX3CL1;STAT3;EBI3;AR;TNFRSF13C;AREG;ATG16L1;AKT1;MECP2;GSN;CTSS	6.06296E-10	0.023255814
Protein regulators of T-cell proliferation	1680	T-cell proliferation	30	1	E2F2;NFATC1;FOXO1;FLT1;NGF;NOS2;AR;AREG;PDE4D;ATG16L1;AKT1;BCL2;CD83;DNAJB1;HIF1A;RELA;IL1RAP;IL2RB;TNC;TNFSF8;CCL22;CX3CL1;STAT3;STAT4;EBI3;TNFRSF13C;SMARCA4;KLRD1;ATG7;CTSS	1.74744E-09	0.01734104
Protein regulators of immune system activation	920	immune system activation	22	2	VWF;NFATC1;HIF1A;CLEC16A;RELA;IL2RB;ADAR;PRKN;FOXO1;FLT1;NGF;NOS2;CCL22;CX3CL1;STAT3;STAT4;TNFRSF13C;SMARCA4;ATG16L1;AKT1;KLRD1;MECP2	2.83886E-09	0.022494888
Protein regulators of lymphangiogenesis	596	lymphangiogenesis	18	3	STAT3;AR;AREG;NFATC1;NFATC2;SMARCA4;AKT1;HIF1A;RELA;ATG7;BCL2;MMP2;FOXO1;ITGA9;FLT1;NGF;TBX1;NOS2	2.90181E-09	0.027355623
Protein regulators of chondrocyte proliferation	469	chondrocyte proliferation	16	3	STAT3;NFATC2;AKT1;HIF1A;SOX5;RELA;H19;ATG7;FOXO1;TNC;NOS1;NOS2;FSTL1;NPPC;CYP19A1;WISP3	4.52394E-09	0.030018762
Protein regulators of bone resorption	1174	bone resorption	24	2	NFATC1;NFATC2;IL34;MST1;HIF1A;RELA;FOXO1;NGF;NOS2;NPPC;CCL22;CX3CL1;STAT3;AR;SLIT3;AKT1;TANK;ATG7;BCL2;GSN;MMP2;CTSS;EIF2AK2;CYP19A1	9.95911E-09	0.019512195
Protein regulators of antigen processing and presentation	1089	antigen processing and presentation	23	2	CD83;HIF1A;CLEC16A;RELA;ITSN1;PRKN;FOXO1;NGF;TNFSF8;NOS1;NOS2;FSTL1;CCL22;CX3CL1;STAT3;STAT4;ATG16L1;AKT1;KLRD1;ATG7;NPC2;CTSS;EIF2AK2	1.20578E-08	0.020069808
Protein regulators of macrophage migration	714	macrophage migration	18	2	STAT3;AR;SMARCA4;AKT1;IL34;MST1;HIF1A;H19;ATG7;MMP2;FLT1;NGF;TNC;NOS2;CTSS;CCL22;CX3CL1;CYP19A1	4.76335E-08	0.023195876
Protein regulators of cartilage homeostasis	229	cartilage homeostasis	11	4	FGF8;PRKN;MMP2;FOXO1;NFATC1;NFATC2;NGF;FSTL1;NPPC;HIF1A;WISP3	4.87202E-08	0.036912752
Protein regulators of leukocyte activation	616	leukocyte activation	16	2	VWF;CD83;STAT3;NFATC1;NFATC2;PDE4D;AKT1;HIF1A;RELA;IL2RB;MMP2;FLT1;NOS2;NPPC;CCL22;CX3CL1	2.01287E-07	0.023529412
Protein regulators of vasculogenesis	623	vasculogenesis	16	2	VWF;STAT4;AR;SMARCA4;AKT1;HIF1A;RELA;MMP2;FOXO1;FLT1;NGF;TNC;NOS1;NOS2;CTSS;NPPC	2.34553E-07	0.023289665
Protein regulators of endothelial cell growth	499	endothelial cell growth	14	2	VWF;E2F2;RPS6KA2;AKT1;HIF1A;BCL2;MMP2;FOXO1;FLT1;NGF;TNC;NOS2;FSTL1;NPPC	5.16744E-07	0.024778761
Protein regulators of regulatory T-cell function	554	regulatory T-cell function	14	2	STAT3;AREG;NFATC1;NFATC2;SMARCA4;UBE2I;AKT1;HIF1A;RELA;BCL2;FOXO1;TNFSF8;NOS2;CYLD	1.79003E-06	0.022580645
Protein regulators of cytokine response	763	cytokine response	16	2	STAT3;STAT4;NFATC2;ATG16L1;AKT1;HIF1A;IL1RAP;NGF;S100B;NOS1;NOS2;CTSS;PRKDC;CCL22;CX3CL1;EIF2AK2	3.40679E-06	0.019347037
